# Heme/NADH Mimics Co‐Decorated Allosteric Capsules for Regulating Oxygen Activation and Upregulating Intracellular Methionine Depletion

**DOI:** 10.1002/advs.202507850

**Published:** 2025-06-27

**Authors:** Chunming Ma, Yihao Zhang, Junkai Cai, Baotong Ding, Yang Jiao, Chunying Duan

**Affiliations:** ^1^ State Key Laboratory of Fine Chemicals Dalian University of Technology Dalian 116024 P. R. China; ^2^ State Key Laboratory of Coordination Chemistry Nanjing University Nanjing 210023 P. R. China

**Keywords:** catalytic oxidation, metal‐organic capsule, methionine depletion, oxygen atom transfer, tumor treatment

## Abstract

Stimuli‐responsive behaviors in living bodies provide the fundamental principles for developing switchable architectures that apply to the direct function regulation. Herein, by incorporating both the *µ*‐oxo bridged Fe^III^‐porphyrins and NADH (nicotinamide adenine dinucleotide) mimics into an allosteric coordination capsule, the regulation of O_2_ activation and the upregulation of intracellular methionine depletion have been achieved, resulting in effective pH‐activated tumor immunotherapy. Upon light irradiation, the transition from the tensed bridged Fe^III^‐porphyrins to the relaxed unbridged Fe^IV^ = O/Fe^II^ porphyrin pair induces unique conformational switching. The relaxed state of the capsule facilitates the binding of substrates for the oxygen atom transfer reaction. The in situ formed Fe^II^‐porphyrin pair, following the substrate oxygenation, activates O_2_ with the intervention of NADH mimics to restore the tensed state of the capsule, thereby releasing the product for the next cycle. Regulated by the acid/base content in the system, this allostery‐triggered metabolic behavior enhances the efficacy of intracellular methionine depletion therapy through the accumulation of local methionine sulfoxide, thereby improving safety. The allosteric switching strategy offers an improved approach for precise tumor treatment via dual light‐and‐guest stimuli‐responsive regulation.

## Graphical abstract

Through modifying the switchable *µ*‐oxo bridged Fe^III^‐porphyrins moiety and NADH mimics into a metal‐organic capsule, multiple responsive manners for O_2_ activation, chemical oxygenation and metabolic transformation have been demonstrated for upregulating intracellular methionine depletion and accumulation of methionine sulfoxide to achieve effective tumor treatment with low cytotoxicity upon light irradiation.

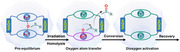

## 1 Introduction

Living allosteric organisms respond to diverse molecular‐scale stimuli through tunable conformational transitions and distinctive binding‐site communication for the transduction and processing of life information, permeating biological receptors and enzymes critical to energy‐driven intracellular metabolic pathways.^[^ ^1^, ^2^ ^]^ Such a unique allosteric responsive mechanism establishes the fundamental principles for developing switchable supramolecular architectures with tailored chemical and physical properties to achieve specific biomimetic functions in molecular machines, molecular devices, biomimetic drugs, and enzymatic catalysis.^[^ ^3^, ^4^ ^]^ So far, most regulation processes in abiotic systems are primarily accomplished by classical effectors in response to a single stimulus, such as pH or chemical species.^[^ ^5^, ^6^ ^]^ Multiple responsive mechanisms for chemical oxygenation and metabolic transformation remain rarely achievable, particularly for systems combining light and autonomous substrate oxidation,^[^ ^7^ ^]^ despite the intracellular light‐induced target oxygenation being essential for tumor photodynamic therapy.

Controlling allosteric properties in the active excited state for oxygen activation, while synergistically coupling multiple responsive behaviors in an orthogonal fashion to obtain the target product, presents significant challenges.^[^ ^8^, ^9^ ^]^ Synthetic coordination assemblies with customizable metal nodes and rigid cavities are widely used to simulate the enzyme‐catalyzed reactions in response to both effectors and light.^[^ ^10^, ^11^ ^]^ Incorporating bioinspired conformational switching motifs into coordination capsules would create cascading effects in chemical transformations, enabling the design of photoactive allosteric catalysts to precisely regulate the target reaction pathways for pharmaceutical intermediates production and tumor therapy.^[^ ^12^ ^]^

Among the biological components related to tumor metabolism, methionine, an essential sulfur‐containing amino acid for the rapid development of tumors, participates in the important intracellular methionine cycle and redox processes.^[^ ^13^, ^14^ ^]^ Recent advances in therapeutic methods that suppress tumor cell proliferation have been associated with secondary damage to normal cells, resulting from interference with the tumor methionine cycle and depletion of the intracellular methionine pools.^[^ ^15^, ^16^ ^]^ The low substrate fidelity and imbalanced intracellular methionine concentration always lead to uncontrollable methionine depletion, which limits the efficacy of clinical treatments.^[^ ^17^ ^]^ Inspired by the transitions between the tensed and relaxed states during oxygen binding in hemoglobin and its mimics,^[^ ^18^, ^19^ ^]^ and the ubiquitous *µ*‐oxo bridged motifs that enhance the intracellular metabolism in metalloenzymes,^[^ ^20^ ^]^ we envision that incorporating a light‐tunable conformational switch corresponding to the *µ*‐oxo bridge element into the allosteric capsule would reconfigure the active sites to facilitate methionine (Met) binding and methionine sulfoxide (MetO) release.

**Figure 1:**
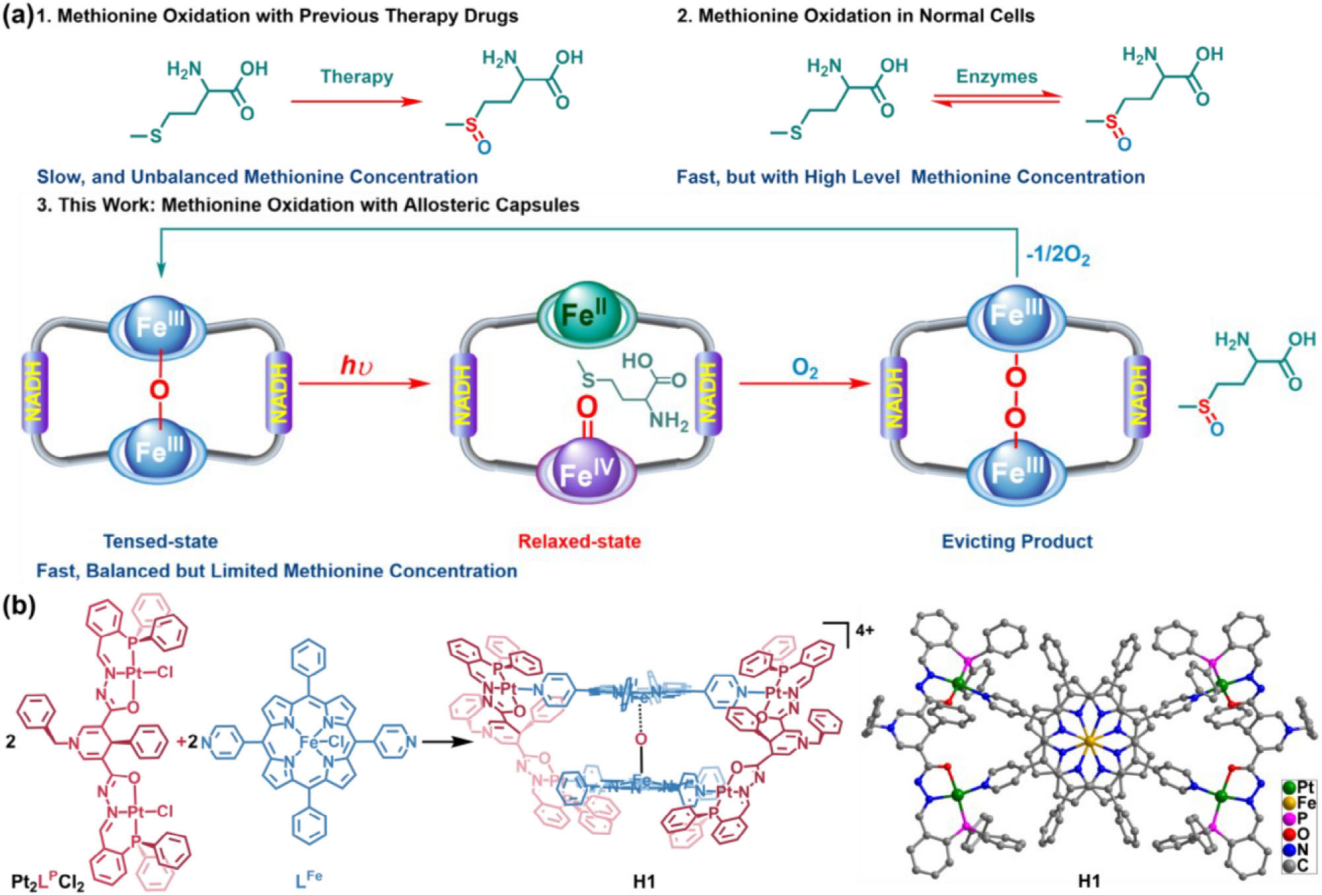
a) The comparison of methionine oxidation by different means. b) Schematic diagram of the self‐assembly of the metal‐organic capsule **H1** from Pt_2_ **L^P^** Cl_2_ and **L^Fe^**, and the top view of the crystal structure of **H1**. Pt green, Fe amber, N blue, O red, P pink, C grey.

Herein, a *µ*‐oxo bridged Fe^III^‐porphyrins and NADH mimics co‐modified Pt^II^ tetragonal capsule is created to upregulate the indicators of intracellular Met depletion and MetO accumulation for tumor suppression and treatment (**Figure** **1**). Upon light irradiation, the tensed *µ*‐oxo bridged Fe^III^‐porphyrins transition into the relaxed unbridged Fe^IV^ = O/Fe^II^ porphyrins, binding thioethers to enforce the spatial proximity, where the in situ formed Fe^IV^ = O fragments oxidize the substrates directly, thereby generating reduced relaxed capsules that recognize and activate O_2_ to restore the original *µ*‐oxo tensed state with the intervention of NADH mimics,^[^ ^21^ ^]^ followed by the release of products. The photo‐responsive allosteric system confines all reaction intermediates and active sites within one working module, thereby hindering the diffusion of active species and enabling high selectivity and a short reaction period with a well‐balanced Met concentration.^[^ ^22^ ^]^ Continuous Met oxidation leads to the accumulation of local MetO, which in turn enhances the immune response, resulting in the polarization of macrophages for tumor immunotherapy.^[^ ^23^, ^24^ ^]^ The allosteric conformational switching enables effective tumor treatment sequences at the molecular level with low cytotoxicity, shifting the paradigm of photo‐responsive allosteric supramolecular assemblies for the regulation of reactive species activation in a multi‐stimuli responsive manner.

## 2 Results and Discussion

**Figure 2:**
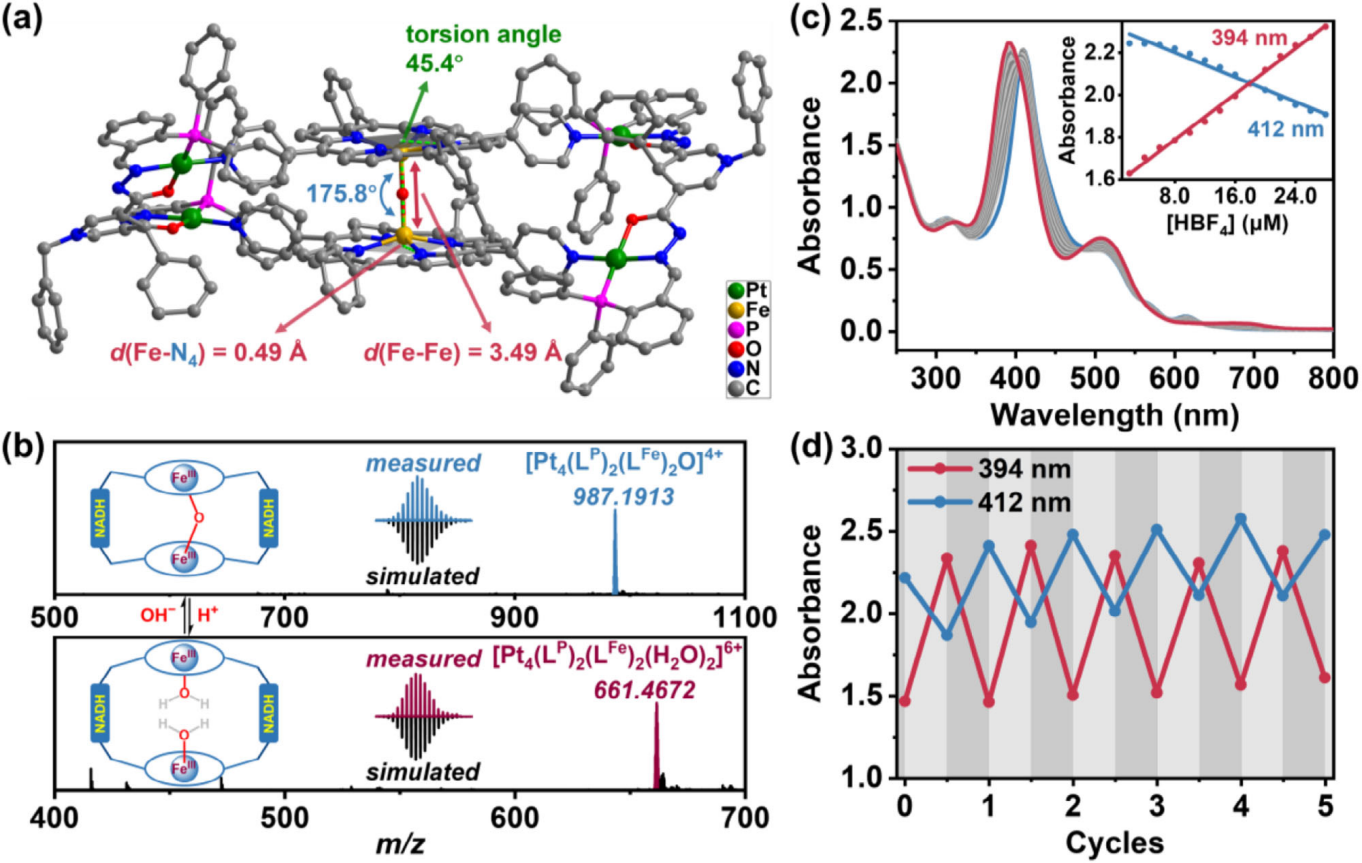
a) Crystal structure of **H1**, showing the angle and length of the Fe^III^–O–Fe^III^ fragment. Pt green, Fe amber, N blue, O red, P pink, C grey. b) ESI‐MS spectra of **H1** (0.5 mM) showed a peak at *m/z* = 987.1913 in the CH_3_CN solution, corresponding to [Pt_4_(**L^P^**)_2_(**L^Fe^**)_2_O]^4+^ (top), and a peak at *m/z* = 661.4672 after adding HBF_4_, corresponding to [Pt_4_(**L^P^**)_2_(**L^Fe^**)_2_(H_2_O)_2_]^6+^ (bottom). c) UV–vis absorption spectra of **H1** (10.0 µM) in MeOH upon the addition of HBF_4_ (total 30.0 µM). Inset: The absorbance of **H1** (10.0 µM) at 394 and 412 nm changed as the concentration of HBF_4_ (considered as [HBF_4_]) increased. d) The absorbance at 394 and 412 nm was monitored during five cycles of switching between the *µ*‐oxo unit and molecular H_2_O on **H1**.

The platinum‐based coordination capsule **H1** was assembled through the reaction of the Pt^II^‐based precursor, Pt_2_ **L^P^** Cl_2_, which was synthesized by reacting K_2_PtCl_4_ and the NADH mimics modified ligand H_2_ **L^P^**,^[^ ^25^, ^26^ ^]^ with the di‐monodentate Fe^III^‐porphyrin ligand **L^Fe^** using a 3:1 complementary denticity directional coordination strategy (Figure 1b).^[^ ^27^, ^28^ ^]^ Single crystal X‐ray structure analysis of **H1** reveals that two Fe^III^‐porphyrin monodentate ligands and two NOP modified tridentate ligands are connected by four Pt^II^ ions in a planar square geometry, creating a tetranuclear macrocyclic capsule (**Figure** **2**a; Figure S6, Supporting Information).^[^ ^29^ ^]^ Notably, two parallel Fe^III^‐porphyrin planes approach each other along the axis of the Fe centers, facilitating the formation of linear *µ*‐oxo bridged Fe^III^–O–Fe^III^ bonds (175.8°) via dechlorination hydration reaction. Each Fe^III^ ion is displaced above the porphyrin plane by 0.49 Å toward the inner space of the capsule, forming a tensed state of **H1** (Figure 2a).^[^ ^30^ ^]^ In this arrangement, two Fe^III^‐porphyrin molecules adopt a cross conformation (with a twist angle of 45.4°) rather than an overlapping mode, thus allowing the two Pt^II^ ions coordinated within a single ligand **L^P^** to bind with different Fe^III^‐porphyrin units (Figure 2a). It maintains interatomic distances of 3.5 Å between two Fe^III^ ions in the Fe^III^–O–Fe^III^ fragment, 8.4 Å between two Pt^II^ ions in a single ligand **L^P^**, and 9.8 Å between the Fe^III^ and Pt^II^ ions in a single ligand **L^Fe^**.

ESI‐MS (electrospray ionization mass spectrometry) spectra of **H1** exhibited intense peaks at *m/z* = 987.1913 and 1365.9046 (Figure 2b; Figure S13, Supporting Information), related to [Pt_4_(**L^P^**)_2_(**L^Fe^**)_2_O]^4+^ and [Pt_4_(**L^P^**)_2_(**L^Fe^**)_2_O(CF_3_SO_3_^−^)]^3+^, respectively, indicating the stability of **H1** in solution. The exclusive presence of the capsule **H1** was confirmed by the diffusion‐ordered NMR spectroscopy (DOSY), which showed a single diffusion coefficient for all resonances (Figure S60, Supporting Information).^[^ ^31^ ^]^ **H1** showed a strong Soret band centered at 412 nm, attributed to the *µ*‐oxo bridged Fe^III^‐porphyrins in the UV‐vis absorption spectrum (Figure S19, Supporting Information). The addition of HBF_4_ (total 30.0 µM) to the **H1** solution resulted in a blue shift of the center of the Soret band from 412 to 394 nm, accompanied by the emergence of several isosbestic points (Figure 2c).^[^ ^32^ ^]^ There was likely an additional step properly related to the protonation of the *µ*‐oxo unit on the capsule **H1**, and a node HBF_4_ concentration that was three times the **H1** concentration led to the formation of a molecular H_2_O binding capsule.^[^ ^33^, ^34^ ^]^ We also recognized that the addition of HBF_4_ to the **H1** solution triggered the appearance of a new peak at *m/z* = 661.4672, corresponding to [Pt_4_(**L^P^**)_2_(**L^Fe^**)_2_ (H_2_O)_2_]^6+^ (Figure 2b; Figure S17, Supporting Information), indicating that the labile Fe^III^–O–Fe^III^ bond in the tensed state of **H1** dissociated into two Fe–H_2_O bonds to form the relaxed state of the capsule by increasing the acidity of the **H1** solution.

After the addition of NaOH, the reversibility of the conformational switching was confirmed by subsequent measurements of UV‐vis absorption spectra showing the preserved characteristic band of the capsule **H1** and ESI‐MS spectra showing peaks at *m/z* = 798.7630 and 998.7075, assigned to [Pt_4_(**L^P^**)(**L^P^_Ox_**)(**L^Fe^**)_2_O(EtOH)]^5+^ and [Pt_4_(**L^P^**)_2_(**L^Fe^**)_2_O(EtOH)]^4+^, (**L^P^_Ox_**: the NAD^+^ mimic decorated ligand **L^P^**), respectively (Figures S41 and S18, Supporting Information). It was noted that the addition of HBF_4_ induced a relaxed state in the capsule, while the subsequent addition of NaOH restored the tensed state of **H1**, and that the switching between the tensed and relaxed states was reversibly cycled five times without a significant decrease in the absorbance (Figure 2d), which was reminiscent of the allosteric regulation in enzymes, and established a solid foundation for efficient and persistent catalytic oxidation of thioether substrates by the capsule **H1**.

**Figure 3:**
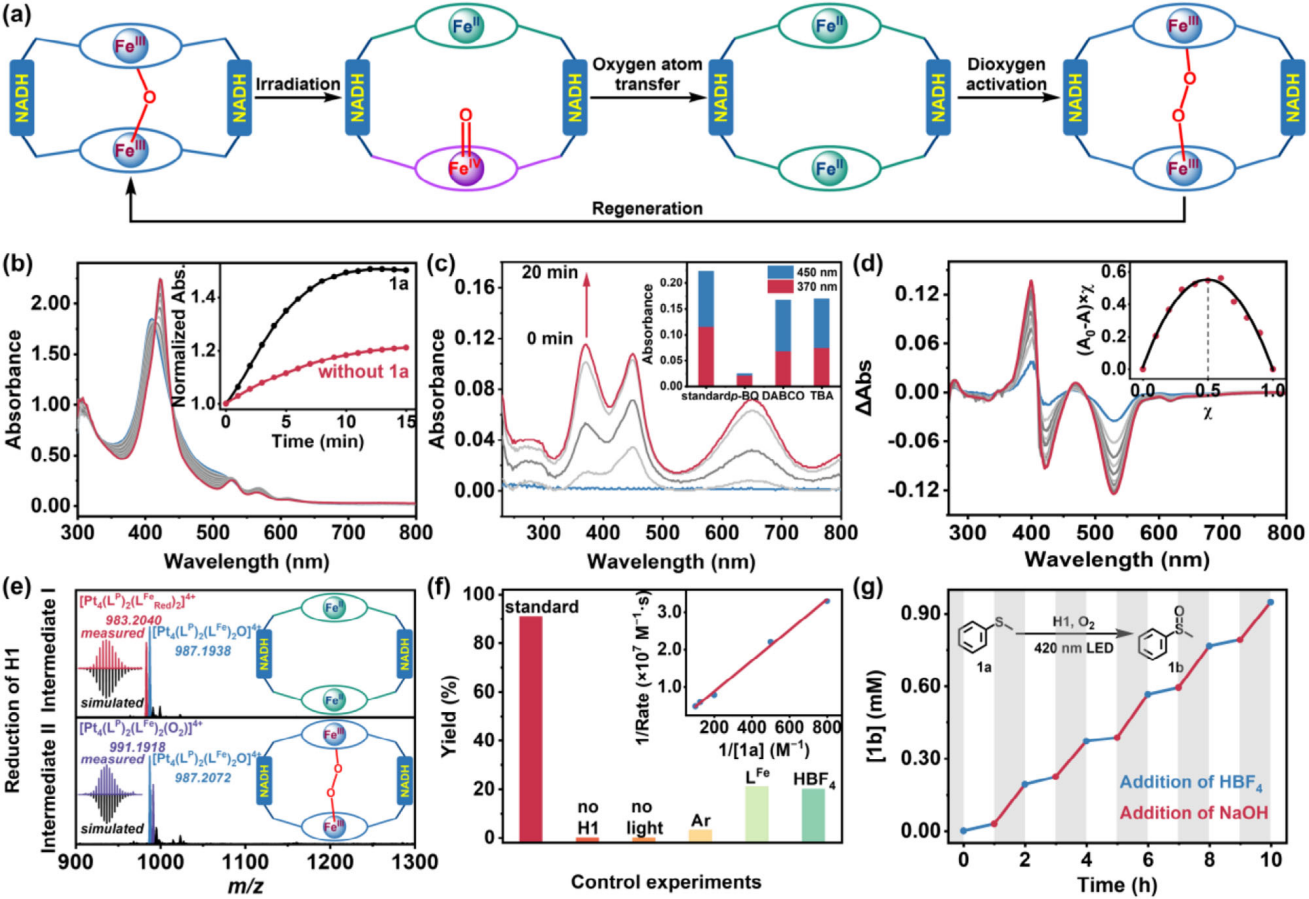
a) Schematic diagram of the conformational transitions of **H1** upon light irradiation in the presence of substrates and O_2_. b) UV–vis absorption spectra of **H1** (10.0 µM) and **1a** (25.0 mM) in anaerobic pyridine upon 420 nm LED irradiation for different times. Inset: The absorbance at 424 nm changed as a function of irradiation time under conditions with or without **1a**. c) UV–vis absorption spectra of TMB in the presence of **H1** in CH_3_COOH/CH_3_COONa (0.1 M/0.1 M) solution upon 420 nm LED irradiation for different times. Inset: The absorbance of the oxidized TMB at 370 and 450 nm in the absence or presence of different scavengers (1.0 equiv.). d) UV–vis absorption spectra of **H1** (10.0 µM) in the MeOH/H_2_O (1:1, v/v) solution upon the addition of **1a** (total 10.0 µM). Inset: Job's plot of **H1** with **1a**. e) ESI‐MS spectra of **H1** (0.5 mM) after the reaction with Na_2_S_2_O_4_ for hours in CH_3_CN. Inset: Schematic diagram of the intermediates. f) Control experiments of the oxidation of **1a** (2.0 mM) upon 420 nm LED irradiation for 12 h. Inset: The Lineweaver‐Burk reciprocal plot of the initial rates of **1a** oxidation by **H1** versus **1a** concentrations under optimized conditions. g) The photo‐induced oxidation of **1a** (2.0 mM) by **H1** (0.1 mM) was performed under optimized conditions through the sequential addition of HBF_4_ (0.2 mM) and NaOH (0.2 mM) for a total of five cycles.

After confirming the contribution of the efficient acid‐base responsive behaviors of **H1** in the controllable conformational transitions, we further investigated the activation state of **H1** in the catalytic processes by employing thioanisole (**1a**) as a sulfide substrate. Given the stability of the *µ*‐oxo bridged Fe^III^‐porphyrins in a slightly alkaline environment, pyridine was used to maintain the tensed state of **H1** in solution. Notably, light stimulation of the **H1** pyridine solution produced several isosbestic points in the absorption spectra, where the center of the Soret band shifted from 410 nm to 424 nm (assigned to Fe^II^‐porphyrin) with an increased intensity, indicating that the tensed *µ*‐oxo bridged Fe^III^‐porphyrins cleaved to form a geminal Fe^IV^ = O/Fe^II^ porphyrin pair upon light irradiation (**Figure** **3**a,b; Figure S21, Supporting Information).^[^ ^35^, ^36^ ^]^ The UV–vis absorption variation intensified in the presence of the oxygen atom transfer reagent **1a** (Figure 3b; Figure S20, Supporting Information), demonstrating that **1a** was able to reduce the Fe^IV^ = O unit to a Fe^II^‐porphyrin moiety (**L^Fe^_Red_**), generating [Pt_4_(**L^P^**)_2_(**L^Fe^_Red_**)_2_]^4+^ species (denoted as reduced host **H_Red_**).^[^ ^37^ ^]^

When 3,3′,5,5′‐tetramethylbenzidine (TMB), an indicator for the formation of reactive oxygen species (ROS),^[^ ^38^ ^]^ was introduced to the **H1** solution, light irradiation resulted in the enhanced absorbance of oxidized TMB at 370, 450, and 652 nm (Figure 3c; Figures S22 and S23, Supporting Information). Subsequently, when divergent scavengers 1,4‐benzoquinone (*p*‐BQ, for superoxide species R‐O_2_^•−^),1,4‐diazabicyclo[2.2.2] octane (DABCO, for singlet oxygen ^1^O_2_), and *tert*‐butanol (TBA, for hydroxyl radical ^•^OH) were added to the solution, respectively, only *p*‐BQ exhibited a significant quenching effect on the oxidation of TMB, whereas other inhibitors showed negligible influence on the reaction (Figure 3c). We deduced that **H1** functioned as a light‐responsive allosteric capsule for efficient oxygen activation and substrate oxygenation, with superoxide as the primary ROS during oxidation.^[^ ^39^ ^]^ In the presence of the reducing agent Na_2_S_2_O_4_ for **H1** in the solution, an intense peak in the HR‐MS spectrum at *m/z* = 983.2040, corresponding to [Pt_4_(**L^P^**)_2_(**L^Fe^_Red_**)_2_]^4+^, was found and assigned to the reduced capsule **H_Red_** in the relaxed state (Figure 3e; Figure S15, Supporting Information).^[^ ^40^ ^]^ Notably, distinct peaks at *m/z* = 1380.5634 assigned to [Pt_4_(**L^P^**)_2_(**L^Fe^**)_2_(O_2_)_2_(PF_6_^−^)]^3+^, where two O_2_^•−^ coordinated to two Fe^III^ centers each, and at *m/z* = 991.1918 assigned to [Pt_4_(**L^P^**)_2_(**L^Fe^**)_2_(O_2_)]^4+^, where one O_2_^2−^ bridged the two Fe^III^ centers, were detected upon air exposure (Figure 3e; Figures S15 and S16, Supporting Information). These results suggested that the relaxed reduced state of **H1** served as a promising model for O_2_ activation, producing Fe^III^–O_2_^•−^ species and Fe^III^–O–O–Fe^III^ intermediates through different pathways (Figure S43, Supporting Information). A signal at *m/z* = 789.5541, assigned to [Pt_4_(**L^P^**)(**L^P^_Ox_**)(**L^Fe^**)_2_O]^5+^, was also observed (Figure S15, Supporting Information). We thought that the NADH mimics modulated ligand **L^P^** might play an auxiliary role in the reductive activation of the O─O bond contained in the Fe^III^−O_2_^•−^ and Fe^III^–O–O–Fe^III^ intermediates during the regeneration of the initial *µ*‐oxo bridged Fe^III^‐porphyrins, when it reacted with the reduced state species, i.e., Fe^II^, it would recover for another cycle of the reaction (Figure S43, Supporting Information).

In summary, light irradiation initially cleaved the Fe^III^–O–Fe^III^ bond in the tensed state of the capsule **H1**, yielding an unbridged Fe^IV^ = O/Fe^II^ porphyrin pair through allosteric conformational transition. Subsequently, the interaction of substrates with Fe^IV^ = O porphyrins produced the reduced form of the capsule **H1** with two isolated Fe^II^‐porphyrins capable of activating O_2_, followed by the formation of O_2_^•−^–Fe^III^‐porphyrin or Fe^III^–O–O–Fe^III^ intermediates, wherein the cleavage of the activated O–O bond generated isolated Fe^IV^ = O porphyrins. With the assistance of the NADH mimics, the geminal Fe^IV^ = O/Fe^II^ porphyrin pair was regenerated or restored to the tensed state of the capsule **H1** with the *µ*‐oxo bridged Fe^III^‐porphyrins for subsequent catalytic cycles.^[^ ^41^ ^]^ The photo‐responsive conformational transition of the capsule **H1** demonstrated efficient oxygen atom transfer behavior toward substrates using oxygen atoms in the Fe^IV^ = O fragments, enabling effective catalytic oxidation of thioether substrates.

Our catalytic experiment started with the oxidation of sulfide **1a**, a simple model of methionine depletion. Upon the addition of **1a**, the UV‐vis absorption spectra of **H1** revealed several isosbestic points, and the Job's plot analysis based on the absorption variation at 412 nm suggested a 1:1 complexation behavior between **H1** and **1a** (Figure 3d).^[^ ^42^, ^43^ ^]^ Under optimized conditions, irradiating a solution containing **1a** (2.0 mM) and **H1** (0.1 mM) with 420 nm light under an O_2_ atmosphere at room temperature effectively produced methyl phenyl sulfoxide (**1b**) in a 91% yield with negligible overoxidation to sulfone over 12 h (Figure 3f), while little product was generated in the absence of **H1**, light, or O_2_, respectively. The high selectivity for sulfoxide formation was possible owing to the lower redox potential of thioether compared to that of sulfoxide.^[^ ^44^ ^]^

Kinetic analysis demonstrated saturation behavior toward the substrates, which was linearized by plotting the double reciprocal of initial rates and substrate concentrations (ranging from 1.25 to 10.0 mM), yielding the *K_m_* of 0.072 M and the *k_cat_* of 0.018 s^−1^, respectively. These findings aligned with the typical Michaelis‐Menten mechanism,^[^ ^45^, ^46^ ^]^ involving the formation of a pre‐equilibrated substrate‐involved clathrate, followed by an oxygen atom transfer reaction driven by the in situ formed Fe^IV^ = O fragment from the photo‐induced cleavage of the Fe^III^–O–Fe^III^ bond and generated the oxidation product **1b**. The reduced form of the capsule, **H_Red_**, was restored to the tensed state with *µ*‐oxo bridged Fe^III^‐porphyrins, releasing products for subsequent catalytic cycles with the intervention of O_2_ and NADH mimics.

Significantly, the addition of HBF_4_ (0.2 mM) quenched the oxygen atom transfer reaction, while subsequent addition of NaOH neutralized the acid and restored the oxygen atom transfer reaction directly. Additionally, the light‐induced catalysis with acid/base switching over several cycles exhibited no significant decomposition of the capsule (Figure 3g). UV–vis titration of **1a** into the **H1** solution containing HBF_4_ (30.0 µM) showed larger spectral changes compared to those observed with free **H1**. Hill plot fitting of titration profiles at 400 nm yielded a larger association constant (1.9 × 10^5^ M^−1^) for the relaxed state of the capsule with **1a**. Further titration of the **H1** solution upon the addition of sulfoxide product indicated that the association constant for **1b** was lower than that for **1a**, regardless of HBF_4_ presence. These results suggested that the relaxed state of the capsule upon irradiation enhanced substrate recognition, while the tensed state of **H1** after oxidation ensured the release of the product. Given the excellent conversion efficiency of sulfide **1a** in the oxygen atom transfer reaction catalyzed by the allosteric capsule, methionine, containing the same methyl sulfide structure, might be selectively and efficiently oxidized by **H1**, thereby promoting Met depletion and MetO accumulation in vivo.^[^ ^47^, ^48^ ^]^

**Figure 4:**
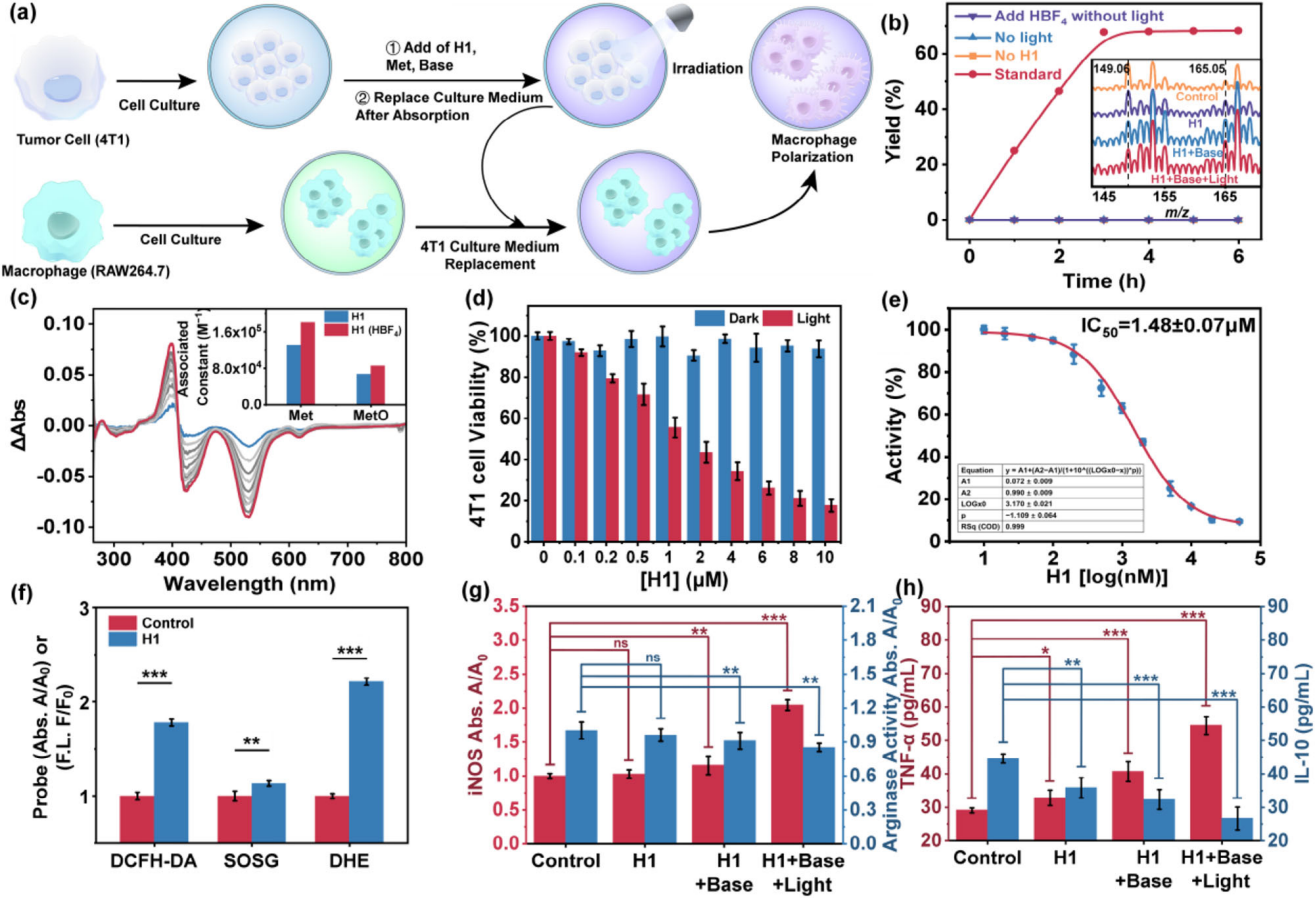
a) Characterization of capsule **H1**’s oxidative ability toward Met and induction of macrophage polarization in vitro. b) The yield of MetO over time under different conditions. Inset: Mass spectra obtained from the supernatant of 4T1 cell culture media under different conditions. c) UV–vis absorption spectra of **H1** (10.0 µM) in the MeOH/H_2_O (1:1, v/v) solution upon the addition of Met (total 10.0 µM). Inset: Association constants obtained from Hill plot fitting of the titration of Met/MetO and **H1**, regardless of HBF_4_ presence. d) 4T1 cell activity under varying concentrations of **H1**, in the presence and absence of light, respectively. e) The dose‐response analysis of **H1** on 4T1 tumor cells under irradiation. f) The ROS production ability of **H1** in 4T1 cells under different environmental conditions upon irradiation. g) The nitric oxide synthase and arginase activity of RAW264.7 cells incubated with 4T1 cell culture media obtained under different conditions. h) Cytokines TNF‐α and IL‐10 levels of macrophages cultured in 4T1‐cell‐conditioned media under different experimental conditions. Data are presented as mean ± SD (n = 6). Differences were analyzed by Student's t‐test or two‐way ANOVA, with significance defined as *p < 0.05, **p < 0.01, and ***p < 0.001 (ns: not significant).

Under optimized conditions, a 5% loading of **H1** achieved 67% conversion of Met to MetO (**Figure** **4**b), an oxidative metabolite that participates in the regulation of signal transduction pathways and is related to the redox status and cell apoptosis,^[^ ^49^ ^]^ after 6 h of irradiation. UV–vis titration of **H1** in the MeOH/H_2_O (1:1, v/v) solution with the addition of Met revealed several isosbestic points. A Job's plot analysis based on the absorption variations at 412 nm suggested a 1:1 complexation behavior between **H1** and Met (Figure 4c). Hill plot fitting of titration profiles indicated an association constant of 1.3 × 10^5^ M^−1^ between Met and the tensed state of the capsule **H1** with *µ*‐oxo bridged Fe^III^‐porphyrins. Upon the addition of HBF_4_ (30.0 µM), **H1** underwent an allosteric transition from the tense state to a relaxed state, where the *µ*‐oxo bridging between Fe^III^‐porphyrin moieties was absent, resulting in more pronounced spectral variations with the addition of Met. Subsequent titration of the **H1** solution by adding the oxidation product MetO demonstrated that the association constant for MetO was lower than that for Met, independent of HBF_4_ presence. The findings suggested that the relaxed state of the capsule upon irradiation or acidification of the **H1** solution enhanced Met recognition, while the tensed state with *µ*‐oxo bridging after oxidation facilitated the release of the product. This provides the possibility for the specific oxidation of Met by the intracellular allosteric capsule and the facilitation of MetO enrichment.

As biocompatibility and effective uptake are fundamental requirements for cellular applications of **H1**, we assessed the uptake and stability of **H1** in physiological environments, and its cytotoxicity toward tumor cells (e.g., 4T1 cells) and immune cells (e.g., macrophages) to determine the feasibility of delivering **H1** capsules. The results of ICP‐MS (inductively coupled plasma mass spectrometry) confirmed that 4T1 cells co‐cultured with **H1** exhibited uptake of ≈846.7 ng (platinum content) at 12 h, indicating that **H1** could be effectively internalized by the cells. The UV–vis absorption changes of **H1** in PBS (phosphate buffered saline) containing 10% FBS (fetal bovine serum) confirmed its stable existence for 24 h under serum conditions (Figure S47, Supporting Information), while no significant hemolysis was observed within the safe concentration range (Figure S46, Supporting Information), thereby confirming the excellent serum stability and biocompatibility of **H1**.

As determined by the MTT (3‐(4,5‐dimethylthiazol‐2‐yl)‐2,5‐diphenyltetrazolium bromide) assay, **H1** exhibited minimal dark toxicity toward 4T1 cells (cell viability >90% at 10 µM) and significant phototoxicity upon light irradiation, with an IC_50_ of 1.48 ± 0.07 µM (Figure 4d,e).^[^ ^50^ ^]^ The results demonstrated the potential of **H1** for tumor therapy via light‐activated mechanisms. Under controlled conditions, 4T1 cells were incubated with various ROS indicators, including DCFH‐DA (2′,7′‐dichlorodihydrofluorescein diacetate, for total ROS), SOSG (singlet oxygen sensor green, for singlet oxygen ^1^O_2_), and DHE (dihydroethidium, for superoxide species R‐O_2_^•−^), respectively,^[^ ^51^, ^52^ ^]^ indicating that **H1** in cells could generate O_2_^•−^ as the dominant ROS, which possessed the potential to oxidize and kill cancer cells and participated in the **H1**‐regulated catalytic oxidation cycle (Figure 4f). Furthermore, the incubation of RAW264.7 cells with **H1** under different culture conditions showed no significant effect on their viability, even at a concentration of 10.0 µM **H1** (Figures S48 and S49, Supporting Information).

Following the determination of the feasibility of **H1**’s role in cells, the catalytic oxidation conversion of Met by **H1** in 4T1 cells was evaluated. As shown in the mass spectra, **H1** led to a more pronounced MetO signal in the presence of light irradiation and alkaline conditions (Figure 4b), while HPLC (high‐performance liquid chromatography) results were simultaneously observed that the MetO signal peak became more significant with the provision of light, pH increase, and time increase, corresponding to the weakening of the Met signal peak. This finding provided substantial evidence for the role of **H1** in light, weak alkaline conditions, and time‐dependent MetO production (Figure S44, Supporting Information), thereby validating the effective catalytic capacity of the allosteric capsule **H1** for Met oxidation in tumor cells.

In the **H1**‐mediated tumor therapy, **H1** not only rapidly oxidized and transformed biomolecules (e.g., Met), thereby disrupting the tumor Met cycle and depleting the Met pool, but also generated MetO (the monooxygenation product of Met), which promoted the polarization of RAW264.7 macrophage cells into diverse subtypes that interacted with tumor cells.^[^ ^53^ ^]^ The strategy of promoting macrophage polarization to the M1 subtype to enhance anti‐tumor immunity has been shown to have great therapeutic potential,^[^ ^54^ ^]^ which was expected to synergize with other treatment options and play an extremely important role in tumor therapy. The subsequent cellular response triggered by Met consumption and MetO accumulation by **H1** in 4T1 cells was investigated (Figure 4a). We characterized the polarization behavior of RAW264.7 cells cultured in Met‐containing media under various conditions using enzyme‐linked immunosorbent assay (ELISA),^[^ ^55^ ^]^ enzyme activity assay, and macrophage flow cytometry staining methodologies (Figure 4g,h; Figure S50, Supporting Information).^[^ ^56^ ^]^ In comparison with the blank group, when both light and bases simultaneously existed, the inducible nitric oxide synthase (iNOS) activity, TNF‐α released, and the expression of CD86 in RAW264.7 cells were increased, indicating the activation of M1 macrophages, whereas the arginase (Arg) activity, released IL‐10, and the expression of CD206 were decreased, confirming the inhibition of M2 macrophages (Figure 4g,h; Figure S50, Supporting Information). These results provided further evidence that supported the efficient oxidative catalytic performance of **H1** with light and bases as stimuli, which could promote the polarization of macrophages to a proinflammatory state.

**Figure 5:**
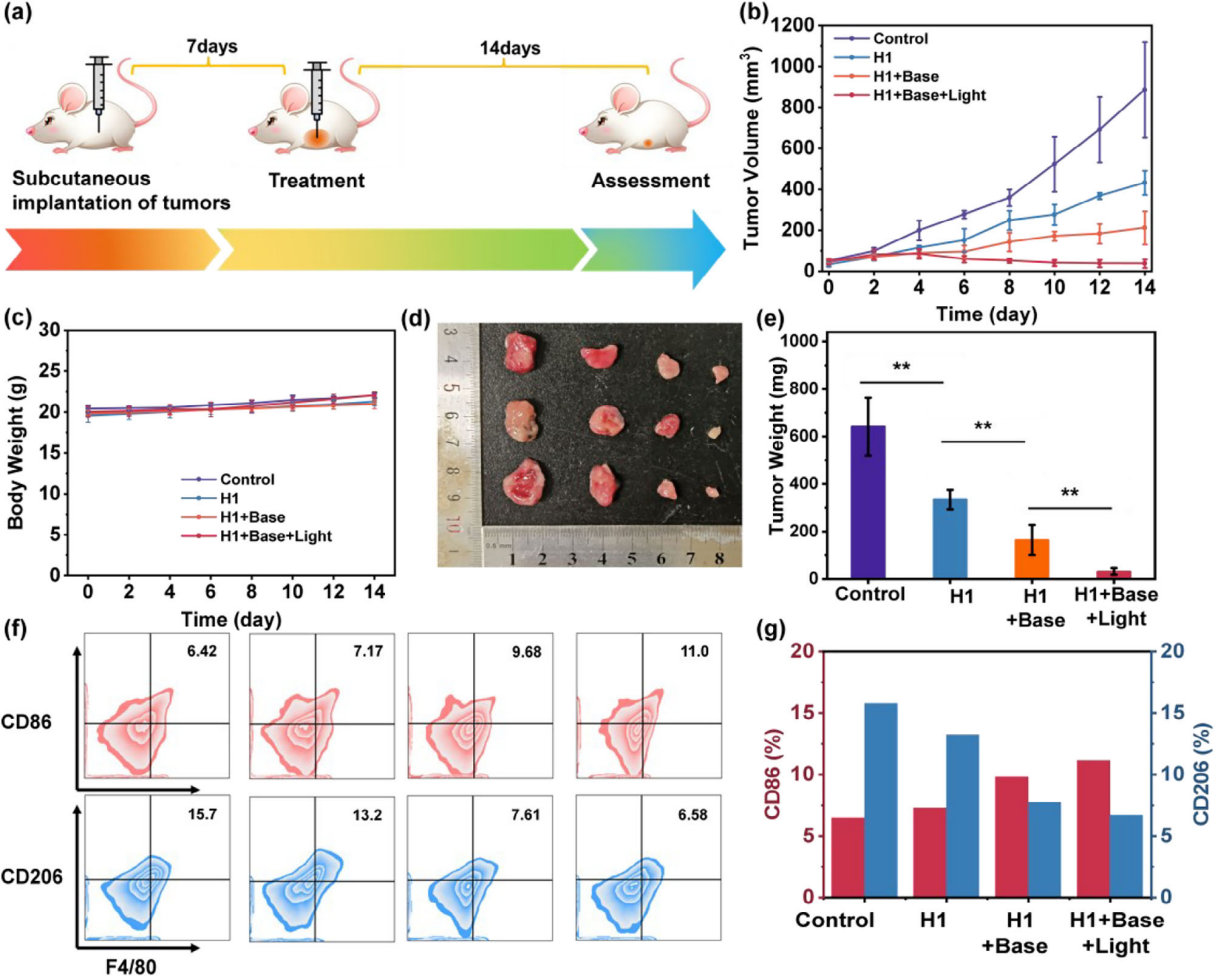
a) Therapeutic procedures for solid tumors in vivo. b) The growth of tumor volume under different conditions during the observation period. c) Changes in the body weight of the mice under various conditions during the observation period. d) Images of tumor tissues from different groups of tumor‐bearing mice (from left to right: Control, **H1**, **H1**&base, **H1**&base&light). e) The average tumor weight of different groups of tumor‐bearing mice. f) Flow cytometry experiment for the detection of macrophage polarization and g) visualized comparison of the corresponding polarization situation. All flow cytometry experiments were repeated three times independently with similar results. Data were presented as mean ± SD (n = 3). Differences were analyzed by Student's t‐test or two‐way ANOVA, with significance defined as *p < 0.05, **p < 0.01, and ***p < 0.001 (ns: not significant).

The anti‐tumor efficacy of **H1** in vivo was validated through the treatment of subcutaneous tumor models of 4T1 breast cancer cells in BALB/c mice (**Figure** **5**a),^[^ ^57^ ^]^ during which body weight increased slightly in all four groups of mice, suggesting negligible systemic toxicity during administration and treatment across all treatment conditions, including **H1** (Figure 5c). Compared to the control group, the co‐injection of **H1** and NaHCO_3_ significantly enhanced the catalytic activity of **H1** under light irradiation, resulting in marked tumor growth inhibition in corresponding treated mice, while due to the inherent cytotoxicity of platinum and the disruption of the acidity of tumor microenvironment,^[^ ^58^, ^59^ ^]^ the tumor growth of mice treated with **H1** and NaHCO_3_ was inhibited to a certain extent without light irradiation (Figure 5b). To evaluate the anti‐cancer effects, mice were euthanized on day 14 and tumor tissues were excised for size and weight comparisons, which revealed significant variations in treatment efficacy (Figure 5d,e). Furthermore, effective immune activation during the in vivo treatment of **H1** under alkaline conditions was investigated to validate the excellent therapeutic efficacy of **H1**, where the increased CD86 expression and the decreased CD206 expression revealed that the Met oxidation was promoted by **H1** in the presence of light and bases, and demonstrated that M1 macrophages were activated while M2 macrophages were inhibited (Figure 5f,g),^[^ ^60^ ^]^ suggesting that the in vivo therapy of **H1** exhibits sustained and multifaceted effects on tumor treatment by effectively inducing macrophage polarization with light and bases as dual stimuli, which provides a new approach for tumor treatment based on Met depletion therapy.

## 3 Conclusion

In summary, a NADH mimics and *µ*‐oxo Fe^III^‐porphyrins modified heteroleptic tetragonal capsule was constructed for light and pH responsive catalytic O_2_ activation and efficient intracellular methionine metabolic transformation. The multiple stimuli derived from light and chemical signals regulated the conformational allostery of the coordination capsule for the divergent catalytic sequences extended to biomedical application, offering new insights to develop multi‐stimuli‐responsive artificial systems for intracellular conversions in complex reaction networks.

## 4 Experimental Section

### Materials and Instrumentation

*General Conditions*: Unless otherwise specified, all chemicals and solvents were of reagent grade quality obtained from commercial sources and used without further purification. The anhydrous acetonitrile was obtained from distillation under reflux and dehydrated by refluxing over CaH_2_. The reaction processes were monitored by thin layer chromatography (TLC), and the products were purified by flash column chromatography on 200–300 mesh silica gel (SiO_2_) or 200–300 mesh neutral aluminum oxide (Al_2_O_3_).

*Nuclear Magnetic Resonance*: ^1^H NMR and ^31^P NMR spectra were recorded on a Bruker 400 M spectrometer at 298 K with chemical shifts reported as ppm (in CDCl_3_, DMSO‐*d*_6_, CD_3_CN‐*d*_3_, or D_2_O). The peak frequencies were referenced versus an internal standard (TMS (tetramethylsilane)) shifts at 0.0 ppm. Coupling constants were recorded in Hertz (Hz). The following abbreviations were used to identify signal multiplicities: s, singlet; d, doublet; dd, doublet of doublets; t, triplet; m, multiplet or overlapping peaks. The ^1^H DOSY spectra were recorded on a Bruker 600 M spectrometer at 298 K (in CD_3_CN‐*d*_3_).

*Electrospray Ionization Mass Spectrometry*: ESI mass spectra were carried out on a Thermo Fisher Q Exactive mass spectrometer using acetonitrile as the mobile phase.

*Ultraviolet–Vis Absorption Spectroscopy*: UV–vis spectra were measured on a Shimadzu UV3600 spectrometer.

*Gas Chromatograph Mass Spectra*: GC‐MS analyses were performed on an Agilent 5977C GC/MSD. The samples were extracted with ethyl acetate (EA) and filtered through 0.22 µm microporous membrane filters to perform the process.

*High‐Performance Liquid Chromatography*: HPLC analyses were performed on Shimadzu LC‐20 AD liquid chromatography with a PDA detector and a ZORBAX SB‐C18 column. The samples were diluted with water and filtered through 0.22 µm microporous membrane filters to perform the process.

*Conditions for the Photocatalytic Reaction*: The light sources were 420 nm LEDs, purchased from Beijing China Education Au‐light (CEAuLight) Technology Co., Ltd. The photon power density was measured by the FZ‐A photo‐radiometer from Beijing Normal University Optoelectronic Instrument Factory.

*Biological Materials*: Penicillin streptomycin solution (dual antibody) (100 x), Trypsin‐EDTA (0.25%), and phosphate buffered saline (PBS) were purchased from Procell Life Science & Technology Co., Ltd. Sheep blood samples were purchased from Shanxi Junma Biotechnology Co., Ltd. Dulbecco's Modified Eagle Medium (DMEM) was purchased from Thermo Fisher Scientific Inc. Fetal bovine serum (FBS) was purchased from PAN Biotech GmbH. 4T1 cells and RAW264.7 cells were purchased from the National Collection of Authenticated Cell Cultures. TNF‐α (E‐EL‐M3063), IL‐10 (E‐EL‐M0046), Elisa kit, PE Anti‐Mouse CD206/MMR Antibody [C068C2] (E‐AB‐F1135D), FITC Anti‐Mouse F4/80 Antibody [CI: A3‐1] (E‐AB‐F0995C), and APC Anti‐Mouse CD86 Antibody [GL‐1] (E‐AB‐F0994E) were purchased from Elabscience Biotechnology Co., Ltd. Four‐week‐old female BALB/c mice were purchased from Liaoning Changsheng Biotechnology Co., Ltd. All animal experiments were approved by the board of the Animal Ethics Committee of Dalian University of Technology (Certificate No./Ethics approval: DUTSC241213‐01).

### Calculation Formula

*τ_4_, a four‐coordinate geometry index, used for the structural analysis*: Inspired by Addison and Reedijk's five‐coordinate *τ*_5_ index, Houser et al proposed a new four‐coordinate metrical parameter *τ*_4_, which could be used to measure the geometries of four‐coordinate transition metal complexes and main group compounds.^[^ ^61^ ^]^

(1) $$\tau_{4}=\frac{360^{\circ }-\left(\alpha +\beta \right)}{141^{\circ }}$$

Of which, *α* and *β* are the two largest angles in the four‐coordinate species. The values of *τ*_4_ will vary from 0 to 1.0, which correspond to perfect square planar geometry (*α* = *β* = 180, *τ*_4_ = 0) and tetrahedral geometry (*α* = *β* = 109.5, *τ*_4_ = 1.0), respectively. Therefore, the simple formula has been used to evaluate the geometries of the Pt atoms. In the crystal structure of **H1**, *α* and *β* are 170.2(18) and 178.8(4) for Pt1, while they are170.1(4) and 170.6(4) for Pt2, from which the value of *τ*_4_ can be calculated to be 0.08 and 0.14 for Pt1 and Pt2, respectively, reflecting that the four‐coordinate geometries around the Pt atoms were close to square planar (Figure S6, Supporting Information).

*Stokes‐Einstein Equation*: DOSY was a nuclear magnetic resonance technique that utilizes pulsed gradient field nuclear magnetic resonance to virtually separate components with different diffusion coefficients (*D*) in a solution.^[^ ^31^ ^]^ The basic principle was the Stokes‐Einstein formula:

(2) $$D=\frac{k_{b}T}{6\pi \eta r_{s}}$$

Of which, *k_b_*, the Boltzmann constant (1.38 × 10^−23^ J K^−1^); *T*, the absolute temperature (298 K); *η*, the viscosity of the solvent; and *r_s_*, the hydrodynamic radius. When the diffusion coefficient of a specific molecular species was obtained, the effective component information can be obtained under controlled conditions, while the hydrodynamic radius of the molecule can be calculated from the formula below:

(3) $$r_{s}=\frac{k_{b}T}{6\pi \eta D}$$

As shown in Figure S60 (Supporting Information), **H1** (2.0 mM) was in CD_3_CN‐*d*_3_ solution (the viscosity *η* of CH_3_CN is 0.37 mN s m^−2^), and the self‐diffusion coefficient (*D*) was 9.55 × 10^−10^ m^2^ s^−1^. The value of *r_s_* obtained by calculation is 0.62 nm for **H1**, which is consistent with the results of the crystal structure of **H1**.

*Michaelis‐Menten Equation*: The Michaelis‐Menten equation was used to verify the mimic enzyme catalytic system that simulates the activation of an enzyme.^[^ ^45^ ^]^

(4) $$V=V_{max}\frac{\left[S\right]}{K_{m}+\left[S\right]}$$

Of which, *V*, the initial reaction rate of the enzymatic reactions; *V*_max_, the maximum reaction rate of the enzymatic reactions; *K_m_*, the Michaelis‐Menten constant; [*S*], the concentration of the substrates. The equation could also transform into another form, like the double‐reciprocal plot shown below:

(5) $$\frac{1}{V}=\frac{K_{m}}{V_{max}}\frac{1}{\left[S\right]}+\frac{1}{V_{max}}$$

### H1‐Induced Photooxidation Reaction

*General Procedure for TMB Oxidation*: Typically, 5.0 mg of TMB was suspended in 3.0 mL of CH_3_COOH/CH_3_COONa (0.1 M/0.1 M) buffer solution. A total of 2.3 mg **H1** was then added to the mixture solution with O_2_ bubbling upon 420 nm LED (30 W) irradiation. The samples were taken at 5 min intervals for UV–vis spectroscopic measurements. In order to verify specific ROS, various scavengers were added to the TMB solution before light irradiation: *p*‐BQ (2.0 mg), DABCO (2.0 mg), and TBA (2.0 µL), respectively (Figure S23, Supporting Information).

*General Procedure for Photocatalytic Oxidation of Thioethers*: 0.1 mM **H1** and 2.0 mM **1a**/Met were dissolved in a 2.0 mL MeOH/H_2_O (1:1, v/v) solution in a quartz reaction tube equipped with circulating cooling water, and the temperature was maintained at 25 °C during the reaction. The reaction mixture was degassed by bubbling O_2_ into the tube for 5 min, after which the tube was capped and equipped with an O_2_‐filled balloon. The photoreaction was performed upon 420 nm LED (30 W) irradiation for 12 h (6 h for Met). The sample solution (50.0 µL) was extracted with EA (0.5 mL), filtered through a 0.22 µm microporous membrane filter, and analyzed by GC‐MS to give the yield of **1b**, while a 50.0 µL sample solution was diluted in Milli‐Q water (0.5 mL), filtered through a 0.22 µm microporous membrane filter, and analyzed by HPLC to give the yield of MetO.

*General Procedure for Kinetics Experiments of the Oxidation*: The reaction system contained catalyst **H1** (0.1 mM) and substrate **1a**/Met (1.25 to 10.0 mM) in a 2.0 mL MeOH/H_2_O (1:1, v/v) solution in the quartz reaction tube with a stirring bar. The reaction mixture was degassed by bubbling O_2_ into the tube for 5 min, after which the tube was capped and equipped with an O_2_‐filled balloon. The photoreaction was performed upon 420 nm LED (30 W) irradiation for 6 h (3 h for Met). During the reaction process, the tracking of the reaction process was carried out by extracting 50.0 µL reaction mixture every 2 h (1 h for Met) with a long needle and followed by GC‐MS (HPLC for Met) analyses after the extraction through EA (dilution in H_2_O for Met) and filtration through 0.22 µm microporous membrane filters. The reaction rates in the first 6 h (3 h for Met) were regarded as the initial rates of the reactions, which were further used in the reciprocal (Lineweaver‐Burk) plot fitting (Figures S35 and S36, Supporting Information).

*General Procedure for Single Photon Oxidation*: In these reactions, **H1** (0.1 mM), **1a** (2.0 mM), MeOH/H_2_O (1:1, v/v, 2.0 mL), and O_2_ as oxidant were charged to a quartz reaction tube and irradiated with 420 nm LED at 25 °C for 6 h. The reaction mixture was degassed by bubbling O_2_ into the quartz reaction tube for 5 min, after which the tube was capped and equipped with an O_2_‐filled balloon. The photon power densities were measured by the FZ‐A photo‐radiometer and recorded before the reaction. During the reaction process, the tracking of the reaction process was carried out by extracting 50.0 µL reaction mixture every 2 h with a long needle and followed by GC‐MS analyses after the extraction through EA (0.5 mL) and filtration through 0.22 µm microporous membrane filters. The reaction rates in the first 6 h were regarded as the initial rates of the reactions, which were further used in the linear plot fitting (Figures S37 and S38, Supporting Information).

*General Procedure for the Determination of the Yield of MetO*: The yield of MetO was analyzed by HPLC using a Shimadzu LC‐20 AD liquid chromatography system with a PDA (photodiode array) detector and a ZORBAX SB‐C18 column. The mobile phase used was water/acetonitrile (95:5, v/v), with a flow rate of 0.5 mL min^−1^ and an injection volume of 30 µL, while the temperature of the column was kept at 25 °C and the wavelength chosen to analyze the results was 210 nm. The sample solution was diluted in water, filtrated through a 0.22 µm microporous membrane filter, and analyzed by HPLC to give the yield of MetO with the peak observed at 4.62 min (Met was at 6.01 min), before which the product was analyzed by ^1^H NMR to confirm the formation of MetO (Figures S39 and S62, Supporting Information).

### Conformational Transition of H1

*General Procedure for the Reduction of* ***H1*** *by Na_2_S_2_O_4_*: In this section, **H1** (0.5 mM) was reacted with an excessive amount of Na_2_S_2_O_4_ in acetonitrile solution under an argon atmosphere for several hours. During the process, 600 µL of the solution was taken out and immediately used as a sample for ESI‐MS analysis after the filtration through a 0.22 µm microporous membrane filter. These results were obtained after multiple samplings. The generation of diverse intermediates might be attributed to the inevitable introduction of air during the ESI‐MS analysis process. **H_Red_** was detected by ESI‐MS, of which the result exhibited an obvious peak at *m/z* = 983.2040, corresponding to [Pt_4_(**L^P^**)_2_(**L^Fe^_Red_**)_2_]^4+^, a capsule containing two iron(II) porphyrin molecules (Figure S15, Supporting Information). Meanwhile, the clear peak at *m/z* = 1380.5634 might be corresponded to [Pt_4_(**L^P^**)_2_(**L^Fe^**)_2_(O_2_)_2_(PF_6_^−^)]^3+^ (intermediate I∙PF_6_), which contained two porphyrin iron(III) superoxide anion radicals (Figure S15, Supporting Information). Besides, in another sampling, the two reduced iron(II) porphyrin molecules in **H_Red_** were transformed into the peroxo‐bridged diiron(III) porphyrins, which were observed by ESI‐MS. The result showed intense signals at *m/z* = 991.1918 and 1369.9092, corresponding to [Pt_4_(**L^P^**)_2_(**L^Fe^**)_2_(O_2_)]^4+^ (intermediate II) and [Pt_4_(**L^P^**)_2_(**L^Fe^**)_2_(O_2_)(PF_6_^−^)]^3+^ (intermediate II∙PF_6_), respectively (Figure S16, Supporting Information).

*General Procedure for the Transformation between µ‐O Bridged and Separated Iron(III) Porphyrins on* ***H1***: Generally, HBF_4_ (0.2 mmol) and NaOH (0.2 mmol) were dissolved in methanol and deionized water to obtain their 2.0 mM solutions, respectively. The UV–vis absorption spectra of **H1** (10.0 µM) in methanol solution (3.0 mL) were collected in a 10 mm quartz colorimetric dish, and 3.0 µL of the above HBF_4_ solution was added at a time. The experiment was carried out until there were no significant changes in the absorbance of the mixture (total 30.0 µM HBF_4_), indicating the formation of the separated iron(III) porphyrins on the capsule (Figure S40, Supporting Information). 3.0 µL of the above NaOH solution was then added to the mixture solution each time, while the UV–vis absorption spectra were recorded. The experiment was performed until there were no significant changes in the absorbance of the mixture (total 30.0 µM NaOH), indicating the regeneration of the *µ*‐O bridged iron(III) porphyrins on the capsule (Figure S41, Supporting Information).

### In Vitro and In Vivo Experiments

*Cell Culture*: 4T1 cells and RAW264.7 cells were cultured in DMEM supplemented with 10% heat‐inactivated FBS and 1% penicillin‐streptomycin (100 U mL^−1^ penicillin and 100 µg mL^−1^ streptomycin) at 37 °C under 5% CO_2_ in normoxic conditions (20% O_2_).

*Cellular Uptake*: The total intracellular accumulation of Pt in 4T1 cells was measured using ICP‐MS (inductively coupled plasma‐mass spectrometry) after treatment with 2.0 µM **H1** for 12 h. The 4T1 cells were inoculated in 75‐cm^2^ culture dishes at an initial density of 5 × 10^6^ cells and subsequently allowed to grow for 24 h. Thereafter, the cells were incubated with 2.0 µM **H1** for 12 h. Subsequently, the cells were harvested by trypsin digestion, washed, and collected using a centrifuge, after which they were dissolved in 2 mL of concentrated nitric acid and heated for 2 h, followed by dilution of the solution to ultrapure water (7 mL), before sending the sample for ICP‐MS detection of Pt. The calculation of the concentrations of Pt can be performed easily by utilizing the standard curve and the CPS values of the samples.

*Serum Stability of* ***H1***: The stability of **H1** in serum was determined by UV‐vis absorption spectroscopy. Generally, several sets of solutions containing 2.0 µM **H1** were prepared in a PBS solution containing 10% FBS, which was freshly prepared. Samples are obtained at 0, 6, 12, 18, and 24 h following preparation to measure UV–vis absorption spectra for comparison.

*Hemolysis Test*: The blood samples from healthy sheep were centrifuged at 2000 rpm for 10 min to collect red blood cells from the serum. The red blood cells were washed repeatedly several times with 10 mL of physiological saline until the supernatant became transparent, before they were diluted to 20 mL of physiological saline. Subsequently, different concentrations of **H1** were added to the red blood cell suspension, which were cultured at 37 °C for 4 h. The hemolysis phenomenon was observed and recorded, while the specific 540 nm spectrophotometric absorption of hemoglobin was monitored. Red blood cells incubated with deionized water were used as positive controls, and all experiments were repeated three times.

In Vitro *Cytotoxicity of* ***H1***: The cytotoxicity of **H1** on 4T1 and RAW264.7 cells was determined by MTT assay in a 96‐well cell culture plate, where 4T1 and RAW264.7 cells were inoculated (5 × 10^3^ cells per well) and incubated in the dark at 37 °C. After 24 h of incubation, the culture media were replaced with 100 µL DMEM containing different concentrations of **H1** for a further incubation of 12 h in the dark at 37 °C, the cells were then washed three times with phosphate‐buffered saline (PBS), while fresh culture media were added, and the cells were exposed to LED light (400 to 830 nm) irradiation for 10 min before a further incubation of 12 h in the dark at 37 °C. Subsequently, 20 µL MTT solution (5.0 mg mL^−1^) was added to each well for further 4 h incubation in the dark at 37 °C, after which the culture media were removed, 100 µL DMSO was added to each well to dissolve the formazan crystals, and the absorbances at 490 nm were read in a microplate reader.

*Measurement of Intracellular ROS Production*: 4T1 cells cultured in a 96‐well plate for 24 h were incubated with 2.0 µM **H1** in the dark at 37 °C for 2 h, and they were then washed with PBS three times and incubated for 30 min with DMEM containing 20.0 µM DCFH‐DA/SOSG/DHE in the dark at 37 °C. Afterwards, the DMEM was removed, while the remaining DCFH‐DA was washed three times with PBS buffer before the cells were subjected to the photosensitization experiment upon LED irradiation (400 to 830 nm) for 10 min, during which the absorbance of DCF and SOSG at 488 nm (the luminescent intensity of DCF and SOSG at 520 nm) and the luminescent intensity of DHE at 580 nm were recorded by a microplate reader.

*Characterization of Met Conversion Behavior in Culture Medium*: 4T1 cells were inoculated into six‐well plates and cultured in DMEM media containing 1.0 mM Met in the dark at 37 °C for 24 h, after which different conditions (blank control, 2.0 µM **H1**, 2.0 µM **H1**&0.35% NaHCO_3_, 2.0 µM **H1**&0.35% NaHCO_3_&light) were then applied to different well plates for 3 h incubation and an equal amount of fresh culture medium was replaced subsequently. Wherein, the light group was irradiated with LED light (400 to 830 nm), while the culture medium of each group was collected for mass spectrometric determination, respectively.

In addition, the catalytic behavior of intracellular Met by **H1** was characterized using HPLC. Generally, 4T1 cells were seeded into a culture dish and cultured at 37 °C for 24 h in DMEM medium containing 4.0 mL of 3.0 mM Met. Then, 2.0 µM **H1** was added and incubated for 12 h, before the medium was discarded, while 1.0 mL of fresh medium was added. The alkalization group was incubated with 0.35% NaHCO_3_ for 1 h to create an alkaline environment. The light group was irradiated with LED light (400 to 830 nm). After completion, the culture dish was frozen overnight, and the system was collected in a centrifuge tube and sonicated for 1 h. The resultant suspension was introduced into a volume of acetonitrile twice its own to yield substantial precipitation of the salts and non‐targeted detection substances, followed by the filtration of the mixture through a 0.22 µm membrane filter, thereby obtaining the sample for the subsequent HPLC analysis (mobile phase: water/methanol (99:1, v/v); flow rate: 0.3 mL min^−1^).

*Nitrite Assays*: The measurement of nitrite concentrations was performed by the Griess reaction. Briefly, 200 µL of the tested cell medium was mixed with 100 µL of 1% sulfanilamide (in 5% phosphoric acid) and 100 µL of 0.3% *N*‐1‐naphthylethylenediamine dihydrochloride (in distilled water) and incubated in the dark at room temperature for 5 min, before nitrites were quantified by spectrophotometry at 540 nm using sodium nitrite as standard.

*Arginase Assays*: The conversion of L‐arginine to L‐ornithine and urea was used for the evaluation of Arginase activity, for which the cells were lysed with 40.0 µL of 0.1% Triton X‐100 for 30 min. Later, 30.0 µL of 25.0 mM Tris‐HCl (pH 7.4) and 10.0 µL of 10.0 mM MnCl_2_ were added and heated at 56 °C for 10 min to activate the enzyme. Similar amounts of samples and 0.5 M L‐arginine were mixed and incubated in the dark at 37 °C for 1 h, and the reaction was stopped by the addition of 400 µL mixture of H_2_SO_4_ (96%), H_3_PO_4_ (85%), and H_2_O (1:3:7, v/v/v). The concentration of urea was measured at 540 nm by spectrophotometry after adding 8.0 µL 6% 2‐isonitrosopropiophenone and heating at 95 °C for 30 min.

*Cytokine Release Assays*: TNF‐α and IL‐10 secreted by macrophages cultured in the conditioned media of 4T1 cells were quantified by ELISA, where the mouse recombinant TNF‐α and IL‐10 were used as standards, and the results were expressed as picogram cytokine per milliliter (pg·mL^−1^).

*Flow Cytometry Experiment for the Detection of Macrophage Polarization*: The cell surfaces were stained with phycoerythrin (PE)‐conjugated anti‐mouse F4/80 antibodies (for the identification of macrophages), FITC‐conjugated anti‐mouse CD86 antibodies (for the identification of M1 macrophages), and APC‐conjugated anti‐mouse CD206 antibodies (for the identification of M2 macrophages). Specifically, the cells were incubated in 5% FBS for 30 min, and the cell surfaces were stained with F4/80 and CD86 antibodies (4 °C, 30 min), after which the cells were washed three times with PBS and fixed in the dark at room temperature for 30 min with the addition of fixation buffer. After removing the fixative by centrifugation (300 × g, 5 min), the cells were resuspended in diluted Permeabilization Wash Buffer and centrifuged at 150 × g for 5 min; the supernatant was then discarded. The washing step was repeated three times. Subsequently, the cells were resuspended in Permeabilization Wash Buffer, and CD206 antibodies were then added and incubated in the dark at room temperature for 30 min, after which the cells were washed three times with Permeabilization Wash Buffer and finally resuspended in 500 µL PBS buffer for detection.

*Mice Treatment*: All mice were randomly divided into four treatment groups and received PBS buffer (control), **H1**, **H1**&NaHCO_3_, and **H1**&NaHCO_3_&weak irradiation treatments, respectively, when the volume of the primary tumor reached ≈50 mm^3^. Distinct groups of **H1** were subcutaneously injected into the tumor area of mice, and the mice were irradiated with LED light (400 to 830 nm) for 30 min at 2 h post‐injection (2 mg kg^−1^). The body weight and tumor volume were recorded over 14 days. The mice were euthanized by inhalation of carbon dioxide on day 14, and the tumor tissues were removed, of which the size and weight were measured and compared with the control group to evaluate the anti‐cancer effect.

### Statistical Analysis

Some experiments in the text were conducted on three or more independent replicates from individual experiments. The number of repetitions of execution was indicated in the corresponding legend. The results were expressed as mean ± standard deviation (SD). All statistical data were processed in Origin 2018. Compare two groups using a one‐sided student t‐test and compare three or more groups using one‐way or two‐way ANOVA according to the experimental design. A *p*‐value less than 0.05 was considered statistically significant. The significance level was expressed as follows: ns (not significant), *p < 0.05, **p < 0.01, ***p < 0.001.

## Conflict of Interest

The authors declare no conflict of interest.

## Supporting information



Supporting Information

## Data Availability

The data that support the findings of this study are available in the supplementary material of this article.
