# Supplementary material for: Heme/NADH Mimics Co‐Decorated Allosteric Capsules for Regulating Oxygen Activation and Upregulating Intracellular Methionine Depletion
Source: Adv Sci (Weinh). 2025 Jun 27;12(36):e07850. doi: 10.1002/advs.202507850 (PMC12463126; doi:10.1002/advs.202507850)
Supplement: Supplementary file 1 — Supporting Information [file ADVS-12-e07850-s001.pdf]

ADVANCED SCIENCE
Open Access

Supporting Information

for *Adv. Sci.*, DOI 10.1002/advs.202507850

Heme/NADH Mimics Co-Decorated Allosteric Capsules for Regulating Oxygen Activation and Upregulating Intracellular Methionine Depletion

Chunming Ma, Yihao Zhang, Junkai Cai, Baotong Ding, Yang Jiao* and Chunying Duan**

Supporting Information for

Heme/NADH Mimics Co-Decorated Allosteric Capsules for Regulating Oxygen Activation and Upregulating Intracellular Methionine Depletion

Chunming Ma,[a] Yihao Zhang,[a] Junkai Cai,*[b] Baotong Ding,[b] Yang Jiao,*[a] and Chunying Duan*[a],[b]

[a]State Key Laboratory of Fine Chemicals, Dalian University of Technology, Dalian 116024, People's Republic of China.

[b]State Key Laboratory of Coordination Chemistry, Nanjing University, Nanjing 210023, People's Republic of China.

*Corresponding Authors. Email: jkcai@nju.edu.cn; jiaoyang@dlut.edu.cn; cyduan@dlut.edu.cn.

Table of Contents

1. Preparation and Characterizations 1

2. Single-crystal X-ray crystallography 6

3. General information for ESI-MS spectra 19

4. UV-vis and kinetics experiments data of the oxidation of substrates by **H1** 27

5. UV-vis titration experiments data of the transformation between *μ*-O bridged and separated iron(III) porphyrins on **H1** 49

6. Proposed mechanism of the oxidation reactions 52

7. In vitro and in vivo experiments 53

8. NMR spectra data 61

9. References 73

1. Preparation and Characterizations

General procedure for the synthesis of ligands and metal-organic compounds

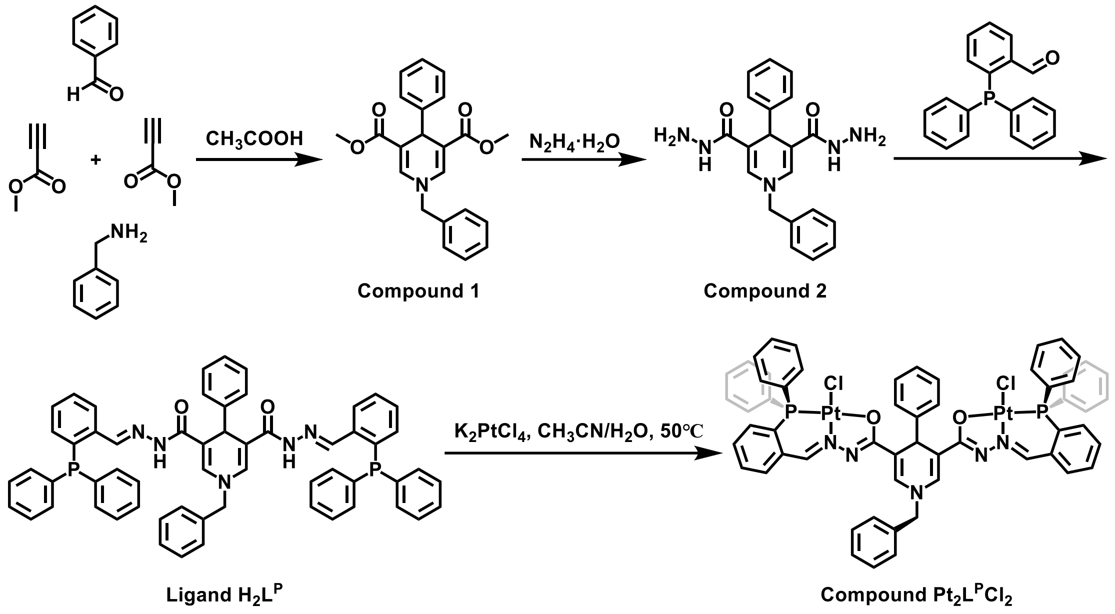

Figure S1. The synthetic route of compound $Pt_2\mathbf{L}^PCl_2$.

Synthesis of compound 1

Glacial acetic acid (2 mL) was added to a dry 100 mL single-necked flask equipped with a magnetic stir bar. Benzaldehyde (1.06 g, 10.0 mmol) and methyl propiolate (1.68 g, 20.0 mmol) were added in succession while stirring. Subsequently, a dropwise addition of benzylamine (1.07 g, 10.0 mmol) to the mixture was performed, followed by heating the mixture at 80ºC for 30 min.[1] Thereafter, the mixture was cooled to room temperature and poured into deionized water (20 mL) with vigorous stirring. After a one-hour stirring period at room temperature, the solid product was filtered and washed with ethyl ether (10 mL × 3) to yield a pure product, which was further recrystallized with ethanol. Compound **1** was collected as yellow crystals through filtration. Yield: 2.20 g, 60.1%. ^{1}H NMR (400 MHz, $CDCl_3$, ppm, 298 K): δ 7.50-7.32 (m, 4H), 7.30-7.24 (m, 5H), 7.24-7.17 (m, 2H), 7.16-7.08 (m, 1H), 4.92 (d, J = 3.1 Hz, 1H), 4.55 (s, 2H), 3.58 (d, J = 3.4 Hz, 6H).

Synthesis of compound 2

A mixture solution of 80% hydrazine hydrate (50 mL) and compound **1** (3.63 g, 10.0 mmol) was stirred at 85°C for 12 h.[1] The precipitate was formed and collected by filtration. The resulting solid was washed with ethanol and dried under vacuum to afford the pale-yellow compound **2**. Yield: 2.15 g, 59.5%. ^{1}H NMR (400 MHz, DMSO-d_6, ppm, 298 K): δ 8.69 (s, 2H), 7.39 (d, J = 6.6 Hz, 2H), 7.34 (d, J = 6.8 Hz, 3H), 7.21 (s, 2H), 7.16 (s, 4H), 7.08 (d, J = 4.3 Hz, 1H), 5.01 (s, 1H), 4.60 (s, 2H), 4.13 (s, 4H).

Synthesis of ligand H_2L^P

Compound **2** (0.36 g, 1.0 mmol) was added to an ethanol solution (20 mL) containing 2-(diphenylphosphino) benzaldehyde (0.58 g, 2.0 mmol). After 3 drops of acetic acid were added, the mixture was heated at 85°C for 12 h.[1] The yellow solid was obtained as ligand H_2**L**^{P} through the process of filtration, subsequent washing with methanol, and final drying under vacuum conditions. Yield: 0.73 g, 80.4%. ^{1}H NMR (400 MHz, DMSO-d_6, ppm, 298 K): δ 11.28 (s, 2H), 8.88 (s, 2H), 7.93 (s, 2H), 7.47-7.27 (m, 24H), 7.17 (s, 12H), 6.79 (dd, J = 7.2, 4.6 Hz, 2H), 5.26 (s, 1H), 4.71 (s, 2H).

Synthesis of compound Pt_2L^PCl_2

The synthesis of compound Pt_2**L**^{P}Cl_2 was improved from the literature method.[2] Ligand H_2**L**^{P} (90.8 mg, 0.1 mmol) and K_2PtCl_4 (83.0 mg, 0.2 mmol) were dissolved in acetonitrile (5 mL) and deionized water (5 mL), respectively. They were both heated to 50°C, followed by the addition of aqueous K_2PtCl_4 solution to the solution containing ligand H_2**L**^{P}, and the mixture was stirred overnight at 50°C. After cooling to room temperature, the mixture was extracted with dichloromethane and deionized water. The organic layer was then dried with anhydrous Na_2SO_4 before the solvent was removed under vacuum, followed by the purification process through column chromatography on neutral Al_2O_3. The pure compound Pt_2**L**^{P}Cl_2 was obtained as an orange solid. Yield: 96.0 mg, 70.2%. ^{1}H NMR (400 MHz, DMSO-d_6, ppm, 298 K): δ 8.83

(s, 2H), 8.15-7.99 (m, 2H), 7.77 (t, J = 7.3 Hz, 2H), 7.69 (t, J = 7.4 Hz, 2H), 7.65-7.30 (m, 31H), 7.16 (t, J = 7.5 Hz, 2H), 7.06 (t, J = 7.2 Hz, 1H), 5.30 (s, 1H), 4.90 (s, 2H). ^{31}P NMR (162 MHz, DMSO-d_6, ppm, 298 K): δ 1.39 (^{195}Pt satellites, $^{1}J_{Pt-P}$ = 1840.3 Hz).

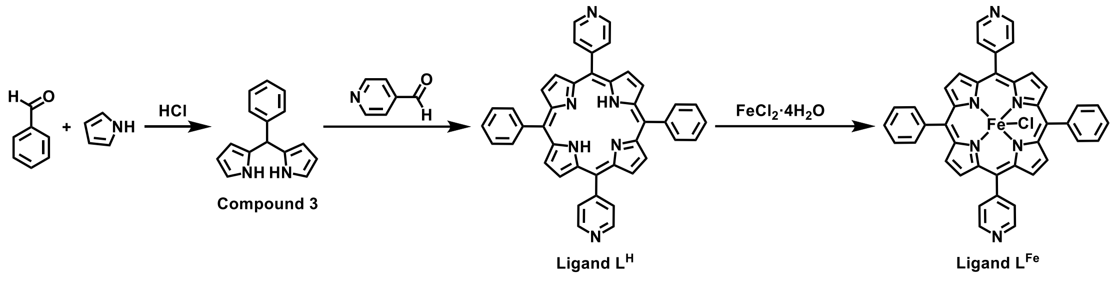

Figure S2. The synthetic route of porphyrin ligands.

Synthesis of compound 3

Concentrated HCl (1.5 mL, 37 %) was added to deionized water (98.5 mL) and the solution was stirred under an argon atmosphere for 10 min. Distilled pyrrole (4.15 mL, 60.0 mmol) was added to this aqueous HCl solution, and the mixture was stirred until a clear solution was obtained. Benzaldehyde (2.0 mL, 20.0 mmol) was added in several portions over 10 min. The reaction mixture was stirred for 6 h at room temperature.[3] The precipitate was then isolated by filtration and thoroughly washed with water. The obtained solid was dried under vacuum. The crude product was purified by column chromatography on silica gel (petroleum ether: dichloromethane = 1:1, v/v) to afford compound **3** as a pale-yellow solid. Yield: 2.29 g, 51.5%. ^{1}H NMR (400 MHz, $CDCl_3$, ppm, 298 K): δ 7.87 (s, 2H), 7.32-7.28 (m, 2H), 7.26-7.18 (m, 3H), 6.67 (d, J = 1.2 Hz, 2H), 6.15 (d, J = 2.5 Hz, 2H), 5.91 (s, 2H), 5.45 (s, 1H).

Synthesis of ligand L^H

Meso-Phenyldipyrromethane (1.0 g, 4.4 mmol) and 4-pyridinecarboxaldehyde (0.42 mL, 4.4 mmol) were dissolved in propionic acid (38 mL) in a 100 mL round-bottom flask equipped with a magnetic stir bar and a water-cooled reflux condenser. The mixture was then allowed to reflux

for 2 h under air. After cooling to room temperature, the reaction mixture was evaporated until dry using a rotary evaporator to yield a dark residue, which was dissolved in dichloromethane subsequently. The mixture was then washed with saturated K_2CO_3 (0.1 M) and water (50 mL), dried over anhydrous Na_2SO_4, and concentrated until dry using a rotary evaporator. The resulting residue was purified by silica gel column chromatography (dichloromethane: methanol = 200:1, v/v). After recrystallization from dichloromethane and methanol, a pure product $\mathbf{L^H}$, was obtained as a purple solid. Yield: 0.11 g, 8.2%. ^{1}H NMR (400 MHz, $CDCl_3$, ppm, 298 K): δ 9.04 (d, J = 5.8 Hz, 4H), 8.95-8.77 (m, 8H), 8.25-8.14 (m, 8H), 7.82-7.73 (m, 6H), −2.85 (s, 2H); ESI-MS ($C_{42}H_{28}N_6$): m/z = 617.24 $[\mathbf{L^H}+H^+]^+$.

Synthesis of ligand $\mathbf{L^{Fe}}$

Ligand $\mathbf{L^H}$ (0.62 g, 1.0 mmol) and $FeCl_2{\cdot}4H_2O$ (1.99 g, 10.0 mmol) were added to 100 mL of a mixed solution of $CHCl_3$/MeOH (7:3, v/v), and the mixture was heated to reflux for 12 h. After cooling down to room temperature, the solvent was removed under vacuum.[1] The obtained solid was washed with deionized water until the filtrate became colorless and dried under vacuum. Ligand $\mathbf{L^{Fe}}$ was obtained as a dark-green solid. Yield: 0.60 g, 85.8%. ESI-MS($C_{42}H_{26}FeN_6$): m/z = 670.15 $[\mathbf{L^{Fe}}-Cl^-]^+$.

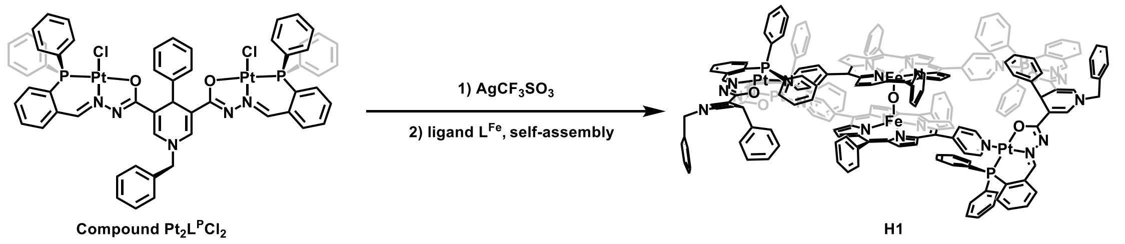

Figure S3. The synthetic route of **H1**.

Synthesis of H1

Compound $Pt_2\mathbf{L^P}Cl_2$ (137 mg, 0.1 mmol) was dissolved in dichloromethane (50 mL) in a 100 mL round-bottom flask, and silver trifluoromethanesulfonate ($AgCF_3SO_3$, 257 mg, 1.0 mmol)

was added to the mixture, which was stirred at room temperature under dark conditions for one day. After the reaction was completed, the insoluble substances were removed by filtration, and the filtrate was concentrated under vacuum until dry. Ligand $\mathbf{L}^{\mathbf{Fe}}$ (106 mg, 0.15 mmol) was then added to the above residue, and 50 mL of fresh anhydrous acetonitrile was added to the mixture, which was stirred at 70°C overnight. After cooling to room temperature, the mixture was filtered to remove the extra ligand $\mathbf{L}^{\mathbf{Fe}}$ and concentrated under vacuum, to which ethyl ether was added for precipitation to obtain the product. The brownish red solid **H1** was collected by filtration and washed with additional ethyl ether. Yield: 198 mg, 86.1%. ^{1}H NMR (400 MHz, CD_3CN-d_3, ppm, 298 K): δ 13.49 (m, 16H), 8.99 (d, J = 15.2 Hz, 4H), 8.46 (d, J = 5.2 Hz, 6H), 8.21 (s, 2H), 8.07 (s, 8H), 8.00-7.67 (m, 64H), 7.61-7.52 (m, 14H), 7.50-7.41 (m, 22H), 5.41 (s, 2H), 4.88 (s, 4H). Capsule **H1** with $CF_3SO_3^-$ as anions for ESI-MS. ESI-MS: ($C_{200}H_{142}Fe_2N_{22}O_5P_4Pt_4$): m/z = 987.1913 $[Pt_4(\mathbf{L}^{\mathbf{P}})_2(\mathbf{L}^{\mathbf{Fe}})_2O]^{4+}$; ($C_{201}H_{142}F_3Fe_2N_{22}O_8P_4Pt_4S$): m/z = 1365.9046 $[Pt_4(\mathbf{L}^{\mathbf{P}})_2(\mathbf{L}^{\mathbf{Fe}})_2O(CF_3SO_3^-)]^{3+}$; ($C_{202}H_{142}F_6Fe_2N_{22}O_{11}P_4Pt_4S_2$): m/z = 2123.3302 $[Pt_4(\mathbf{L}^{\mathbf{P}})_2(\mathbf{L}^{\mathbf{Fe}})_2O(CF_3SO_3^-)_2]^{2+}$.

2. Single-crystal X-ray crystallography

First, the anions of **H1** were exchanged into PF_6^- in advance by adding an excess of NH_4PF_6 to the acetonitrile solution of **H1**, followed by stirring for 5 min. A large amount of distilled water was then added to remove the extra NH_4PF_6, while the solid **H1** was obtained through filtration and washed with ethyl ether. The single crystals of **H1** suitable for X-ray diffraction analysis were obtained by vapor diffusion of ethyl ether into acetonitrile/toluene (1:1, v/v) solution for two weeks. The mass spectrometry of **H1** was performed before the growth of single crystals (Figure S14).

Single-crystal X-ray diffraction data collection for capsule **H1** was performed using a Bruker Smart Apex CCD diffractometer at 193 K. The data were measured using φ and ω scans of 0.9° per frame for 15 s with CuKα (λ = 1.54178 Å) radiation and acquired using the SMART and SAINT programs.[4,5] The structure was solved by direct methods and refined by full matrix least-squares methods by the program SHELXL-2019.[6] The SQUEEZE subroutine in PLATON was used.[7] In the structural refinement of **H1**, anisotropic displacement parameters were used to refine all non-hydrogen atoms. The hydrogen atoms within the ligand backbones were fixed geometrically at calculated distances and allowed to ride on the parent non-hydrogen atoms. The H atoms of the water molecules in **H1** were first located from the Fourier maps. All other H atoms were placed in the geometrically idealized positions and constrained to ride on their parent atoms.

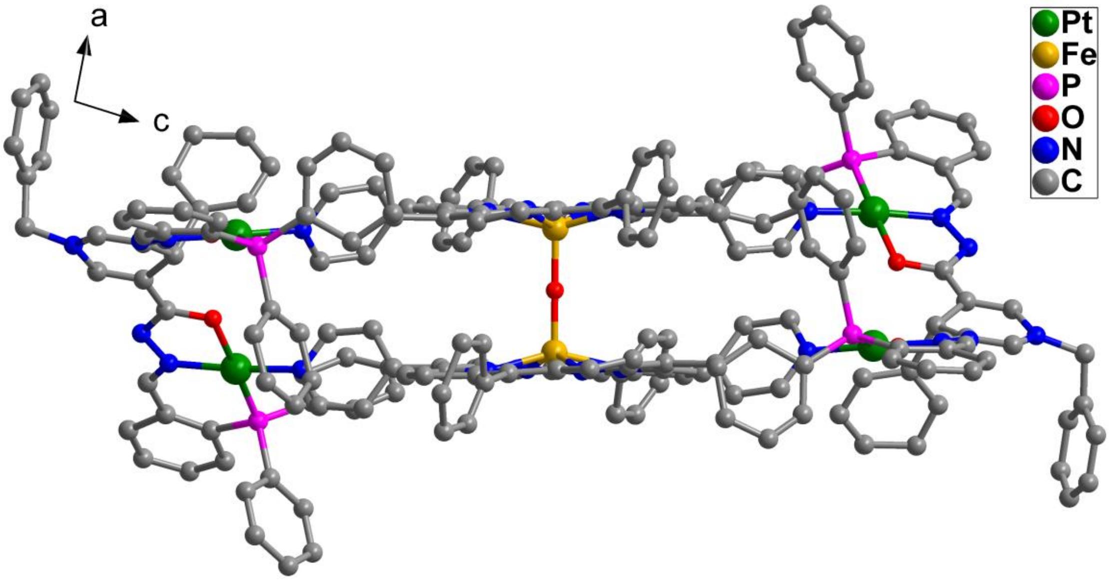

Figure S4. The side view of the crystal structure of metal-organic capsule **H1**. Crystal structure of capsule **H1** viewed from the *b*-axis, showing the Fe−O−Fe fragment with an angle of 175.8°. Hydrogen atoms, counterions, and solvent molecules were omitted for clarity. Symmetry code: [1]1−X, +Y, 1/2−Z.

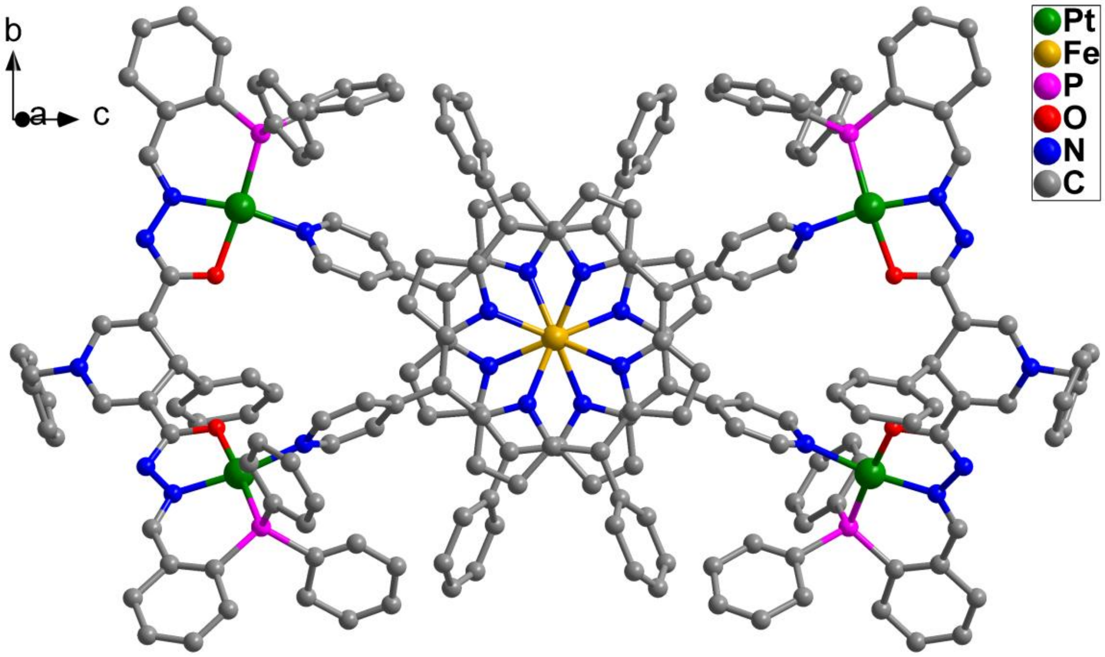

Figure S5. The top view of the crystal structure of metal-organic capsule **H1**. Crystal structure of capsule **H1** viewed from special axis (−1, 0, 0.06), showing the cross conformation of the two iron(III)-porphyrin molecules (twist angle = 45.4°). Hydrogen atoms, counterions, and solvent molecules were omitted for clarity. Symmetry code: [1]1−X, +Y, 1/2−Z.

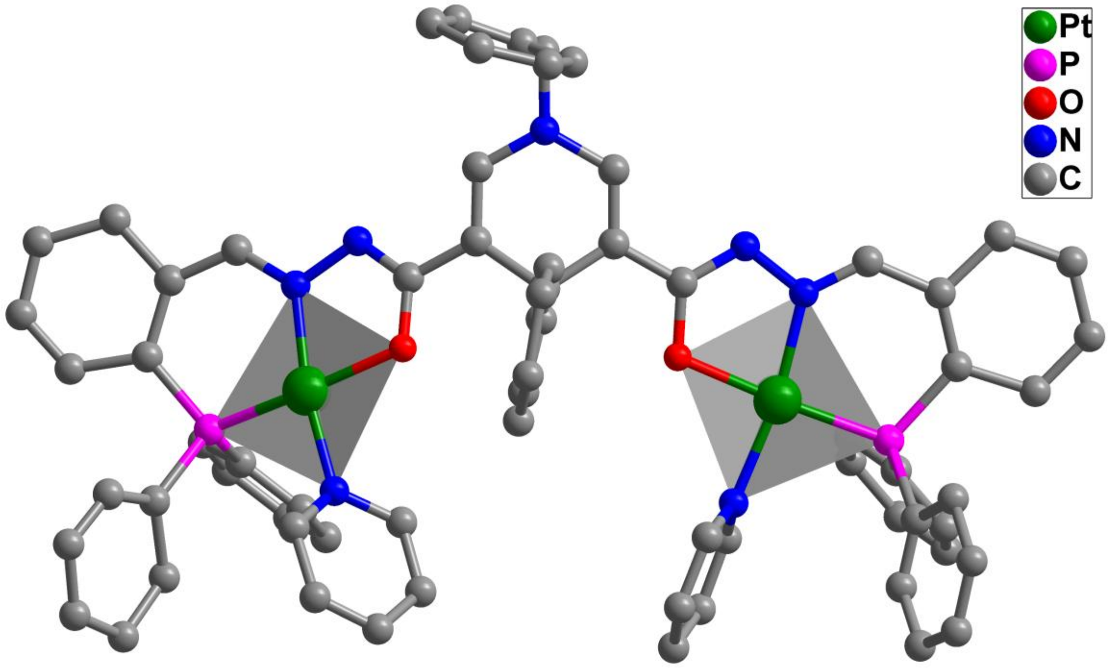

Figure S6. Coordination environments of Pt atoms in **H1** are close to planar square geometries.

Symmetry code: [1]1−X, +Y, 1/2−Z.

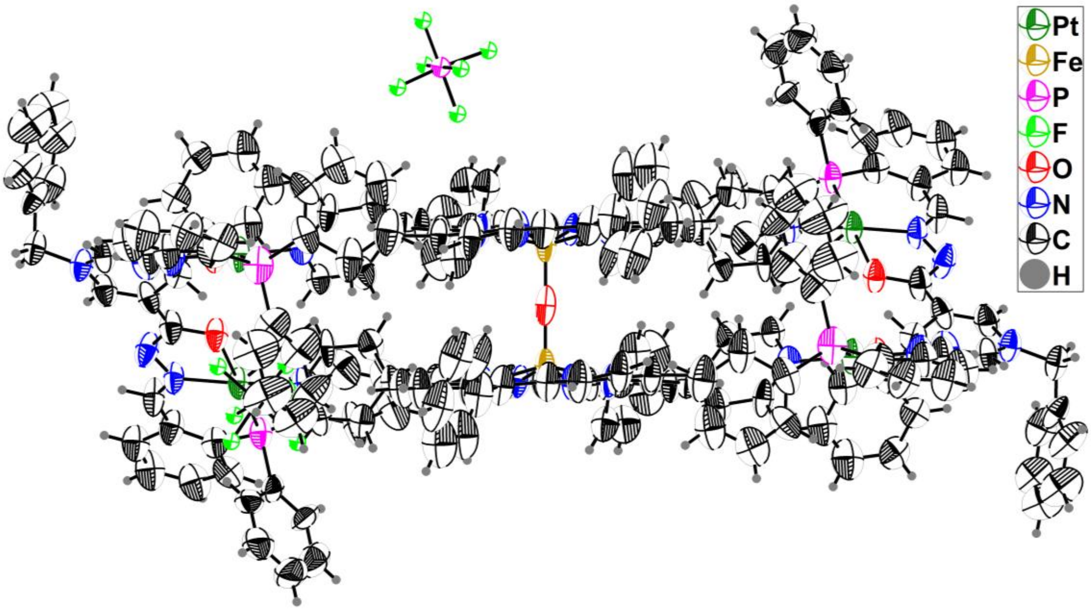

Figure S7. An ORTEP plot of the molecular capsule **H1**, showing 30% probability displacement ellipsoids of non-hydrogen atoms. Symmetry code: ^{1}1−X, +Y, 1/2−Z.

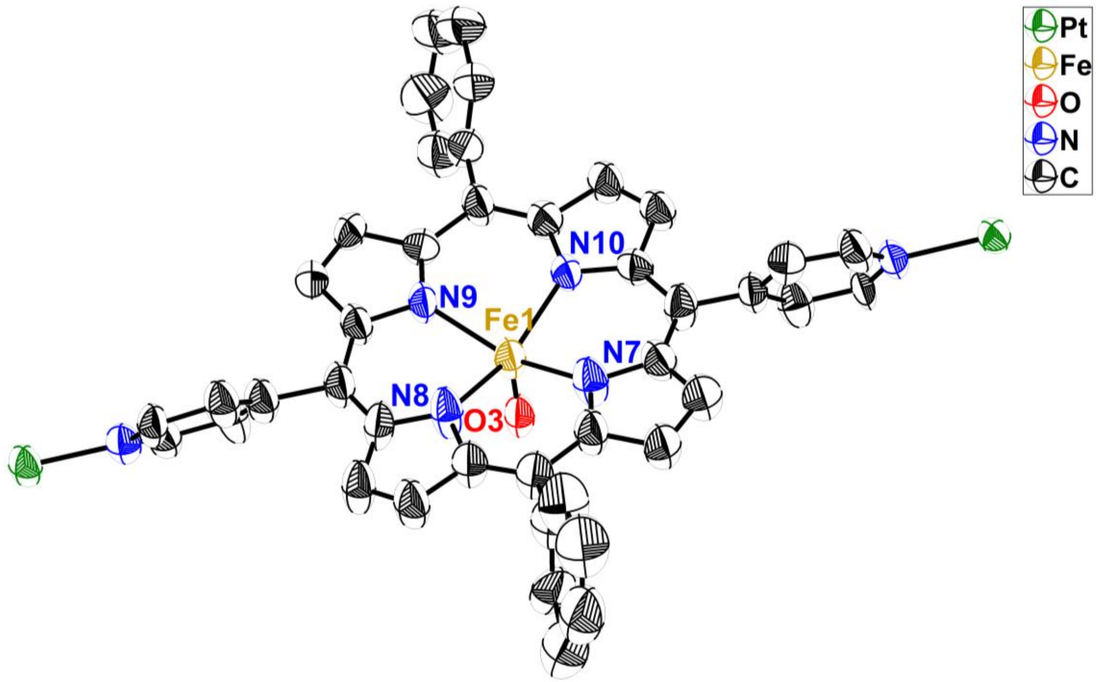

Figure S8. The coordination configuration of Fe1, showing 30% probability displacement ellipsoids of atoms. Selected bond distances (Å) and angles (°): Fe1-O3 1.747(3), Fe1-N7 2.085(6), Fe1-N8 2.087(9), Fe1-N9 2.081(10), Fe1-N10 2.084(7), O3-Fe1-N7 102.8(3), O3-Fe1-N8 105.2(5), O3-Fe1-N9 104.6(4), O3-Fe1-N10 102.2(5). Symmetry code: [1]1−X, +Y, 1/2−Z.

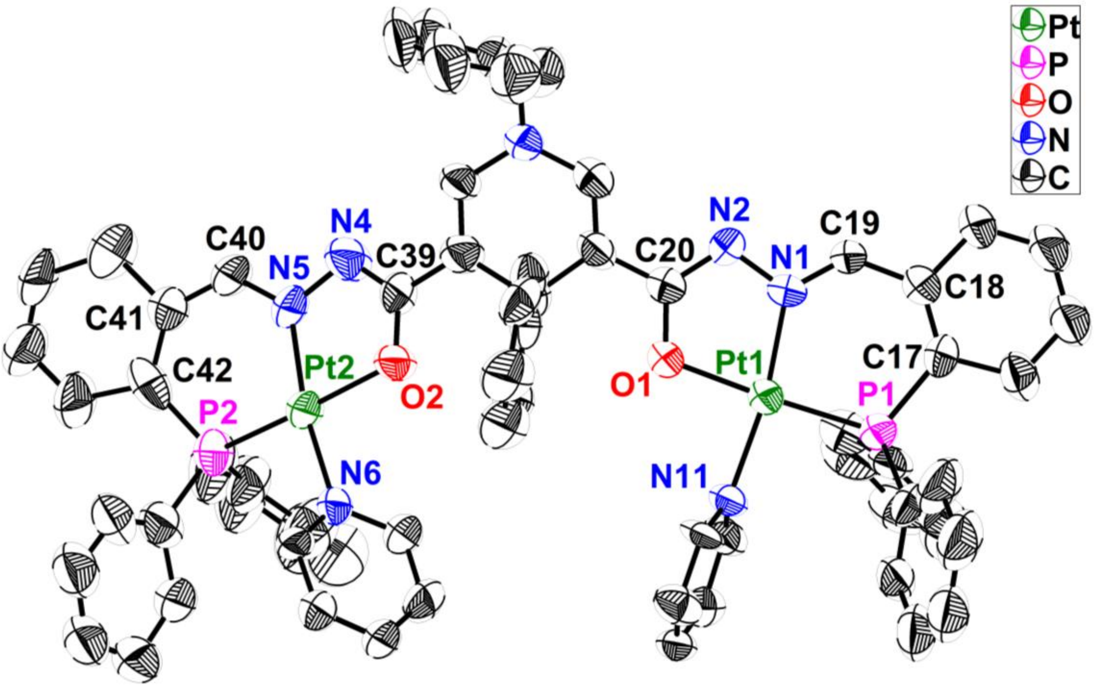

Figure S9. The coordination configuration of the Pt1 and Pt2, showing 30% probability displacement ellipsoids of atoms. Selected bond distances (Å) and angles (°): Pt2-O2 2.025(8), Pt2-N6 1.984(6), Pt2-P2 2.229(5), Pt2-N5 1.970(11), Pt1-O1 1.994(10), Pt1-N11 2.062(8), Pt1-P1 2.209(6), Pt1-N1 1.942(11), N6-Pt2-O2 90.8(4), N6-Pt2-P2 95.4(3), N5-Pt2-P2 94.0(4), N5-Pt2-O2 79.9(4), O2-Pt2-P2 170.1(4), N6-Pt2-N5 170.6(4), N11-Pt1-O1 91(2), N11-Pt1-P1 90(2), N1-Pt1-P1 98.1(4), N1-Pt1-O1 80.8(4), O1-Pt1-P1, 178.8(4), N1-Pt1-N11, 170.2(18). Symmetry code: ^{1}1−X, +Y, 1/2−Z.

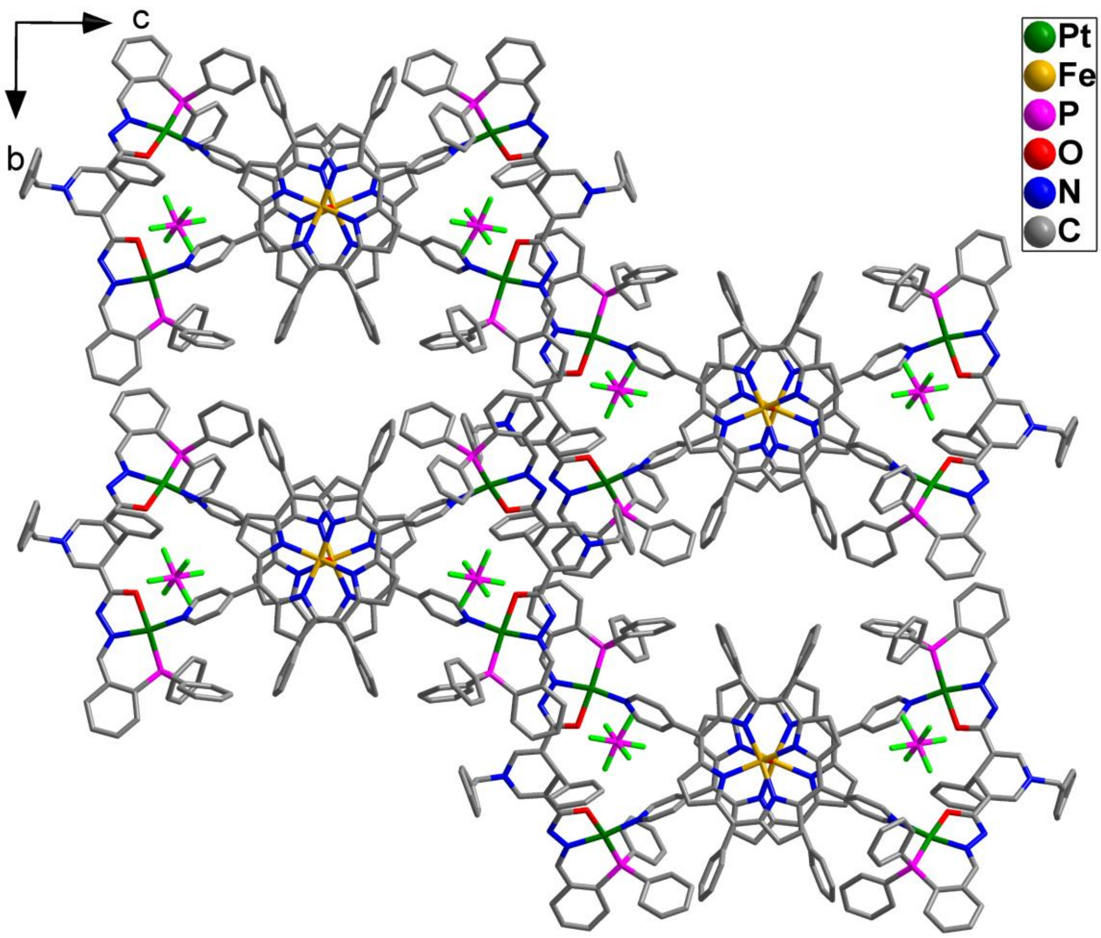

Figure S10. Crystal packing model of capsule **H1** with counterions viewed from the *a*-axis. Hydrogen atoms and solvent molecules were omitted for clarity.

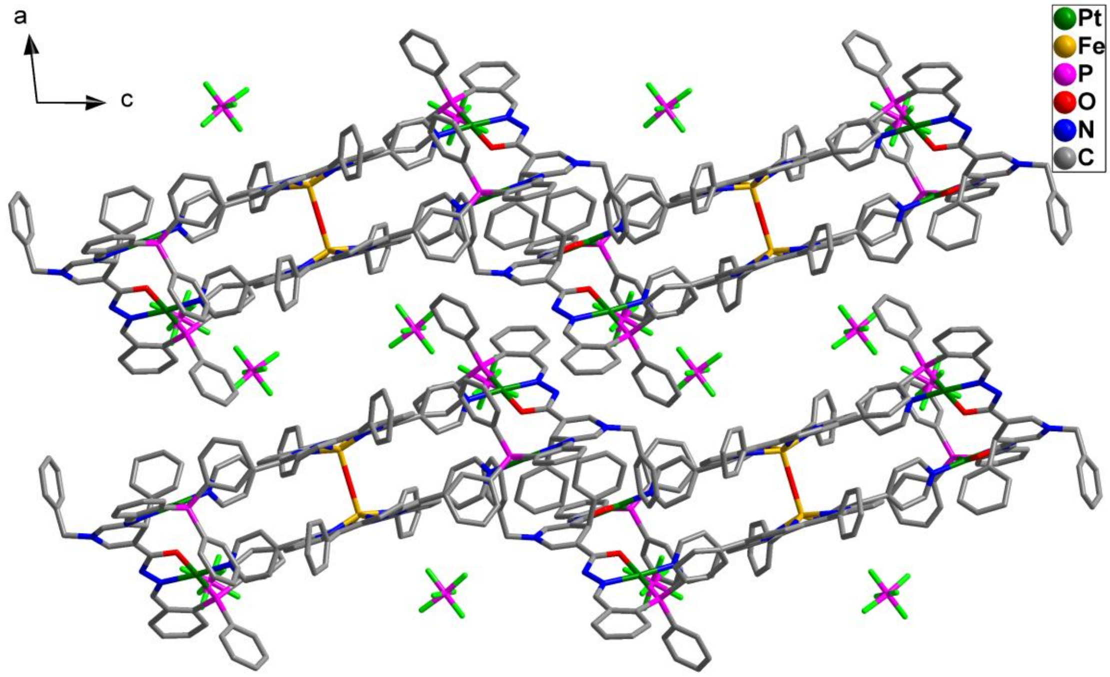

Figure S11. Crystal packing model of capsule **H1** with counterions viewed from the *b*-axis. Hydrogen atoms and solvent molecules were omitted for clarity.

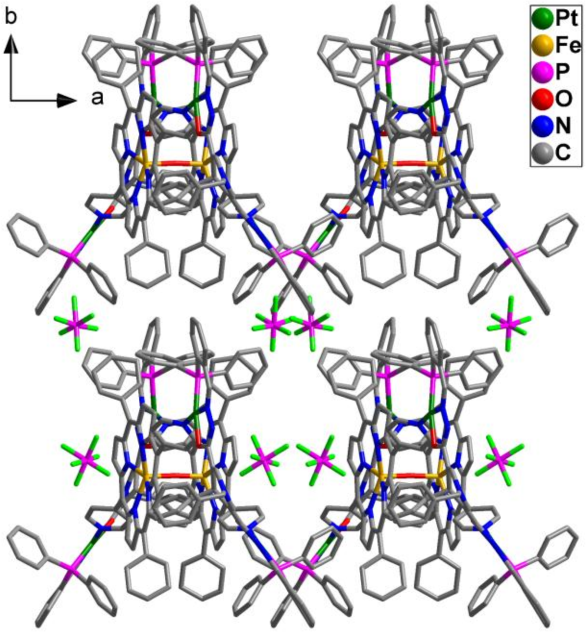

Figure S12. Crystal packing model of capsule **H1** with counterions viewed from the *c*-axis. Hydrogen atoms and solvent molecules were omitted for clarity.

Table S1. Crystallographic data and structure refinement for **H1**.

Compound	**H1**
CCDC number	2369376
Empirical formula	$C_{200}H_{142}F_{24}Fe_2N_{22}O_5P_8Pt_4$
Formula weight	4529.17
Temperature / K	193.0
Crystal system	Monoclinic
Space group	P2/c
a / Å	13.765(4)
b / Å	18.142(3)
c / Å	45.839(9)
α /°	90
β /°	96.636(17)
γ /°	90
Volume /$Å^3$	11371(4)
Z	2
ρ_{calc} g/cm^3	1.323
μ /mm^{-1}	6.613
F(000)	4472.0
Crystal size / mm^3	0.33 × 0.31 × 0.3
Radiation	CuKα (λ = 1.54178)
2θ range for data collection /°	5.244 to 133.188
Index ranges	-16 ≤ h ≤ 16, -21 ≤ k ≤ 21, -54 ≤ l ≤ 52
Reflections collected	62222
Independent reflections	19897 [R_{int} = 0.0887, R_{sigma} = 0.0817]
Data/restraints/parameters	19897/778/1068
Goodness-of-fit on F^2	1.014
Final *R* indexes [I>=2σ(I)]	R_1 = 0.0873, wR_2 = 0.2329
Final *R* indexes [all data]	R_1 = 0.1462, wR_2 = 0.2659
Largest diff. peak/hole /$eÅ^{-3}$	0.94/-0.65

$R_1 = \sum||F_o|-|F_c|\sum/||F_o|$. $wR_2 = [\sum w(F_o^2-F_c^2)^2/\sum w(F_o^2)^2]^{1/2}$

Table S2. Selected bond lengths [Å] for **H1**.

Bond	Length [Å]	Bond	Length [Å]
Pt2-P2	2.229(5)	N5-C40	1.333(14)
Pt2-O2	2.025(8)	N2-C20	1.329(15)
Pt2-N5	1.970(11)	N9-C86	1.407(15)
Pt2-N6	1.984(6)	N9-C89	1.374(16)
Fe1-O3	1.747(3)	N4-C39	1.282(14)
Fe1-N8	2.087(9)	C24-N3	1.3900
Fe1-N9	2.081(10)	N3-C25	1.3900
Fe1-N7	2.085(6)	N3-C26	1.497(14)
Fe1-N10	2.084(7)	C83-N11	1.3900
P1-C17	1.827(7)	N11-C84	1.3900
P1-C4	1.803(8)	C100-N10	1.4200
P1-C7	1.773(10)	N10-C97	1.4200
P1-Pt1	2.209(6)	C63-N6	1.3900
P2-C42	1.820(9)	N6-C59	1.3900
P2-C53	1.775(11)	C22-C33	1.637(11)
P2-C47	1.862(14)	C23-C39	1.487(13)
P3-F1	1.611(14)	C77-C76	1.442(17)
P3-F2	1.626(13)	C77-C78	1.298(18)
P3-F3	1.606(14)	C69-C76	1.400(17)
P3-F4	1.576(16)	C69-C70	1.449(14)
P3-F5	1.648(16)	C26-C27	1.44(2)
P3-F6	1.539(11)	C80-C81	1.485(13)
O1-C20	1.381(15)	C80-C86	1.415(17)
O1-Pt1	1.994(10)	C80-C79	1.416(16)
O2-C39	1.298(14)	C64-C100	1.358(14)
N1-N2	1.371(15)	P4-F12	1.478(16)
N1-C19	1.311(15)	P4-F11	1.473(16)
N1-Pt1	1.942(11)	P4-F10	1.538(18)
N8-C76	1.400(15)	P4-F8	1.508(17)
N8-C79	1.359(14)	P4-F7	1.672(19)
N5-N4	1.440(15)	P4-F13	1.551(17)

Symmetry code: ^{1}1−X, +Y, 1/2−Z.

Table S3. Selected bond angles [°] for **H1**.

Bond	Bond Angles [°]	Bond	Bond Angles [°]
O2-Pt2-P2	170.1(4)	C19-N1-N2	115.0(11)
N5-Pt2-P2	94.0(4)	C19-N1-Pt1	128.9(10)
N5-Pt2-O2	79.9(4)	C76-N8-Fe1	127.2(7)
N5-Pt2-N6	170.6(4)	C79-N8-Fe1	124.8(8)
N6-Pt2-P2	95.4(3)	C79-N8-C76	105.2(9)
N6-Pt2-O2	90.8(4)	N4-N5-Pt2	112.7(7)
O3-Fe1-N8	105.2(5)	C20-N2-N1	113.1(11)
O3-Fe1-N9	104.6(4)	C86-N9-Fe1	127.1(8)
O3-Fe1-N7	102.8(3)	C89-N9-Fe1	125.5(9)
O3-Fe1-N10	102.2(5)	C65-N7-Fe1	124.3(4)
N9-Fe1-N8	86.6(4)	C68-N7-Fe1	126.8(4)
N9-Fe1-N7	152.6(5)	O1-Pt1-P1	178.8(4)
N9-Fe1-N10	87.1(3)	O1-Pt1-N11^{1}	91(2)
N7-Fe1-N8	86.4(3)	N1-Pt1-P1	98.1(4)
N10-Fe1-N8	152.6(5)	N1-Pt1-O1	80.8(4)
N10-Fe1-N7	87.1(3)	N1-Pt1-N11^{1}	170.2(18)
C17-P1-Pt1	110.6(4)	N11^{1}-Pt1-P1	90(2)
C4-P1-C17	107.1(5)	F1-P3-F2	176.4(10)
C4-P1-Pt1	112.0(4)	F1-P3-F5	88.2(10)
C7-P1-C17	104.3(5)	F2-P3-F5	88.6(9)
C7-P1-C4	107.7(6)	F3-P3-F1	89.4(8)
C7-P1-Pt1	114.7(4)	F3-P3-F2	92.1(9)
C42-P2-Pt2	111.6(4)	F3-P3-F5	85.7(10)
C42-P2-C47	107.3(7)	F4-P3-F1	95.5(11)
C53-P2-Pt2	118.1(4)	F4-P3-F2	87.8(10)
C53-P2-C42	105.3(6)	F4-P3-F3	89.8(9)
C53-P2-C47	105.3(8)	F4-P3-F5	174.1(10)
C47-P2-Pt2	108.7(5)	F6-P3-F1	89.5(7)
Fe1^{1}-O3-Fe1	175.8(6)	F6-P3-F2	89.0(8)
C20-O1-Pt1	110.4(7)	F6-P3-F3	178.4(9)
C39-O2-Pt2	111.0(7)	F6-P3-F4	91.4(8)
N2-N1-Pt1	116.0(7)	F6-P3-F5	93.2(7)

Symmetry code: ^{1}1−X, +Y, 1/2−Z.

3. General information for ESI-MS spectra

3.1 ESI-MS spectra of H1 with $CF_3SO_3^-$/PF_6^- as anion

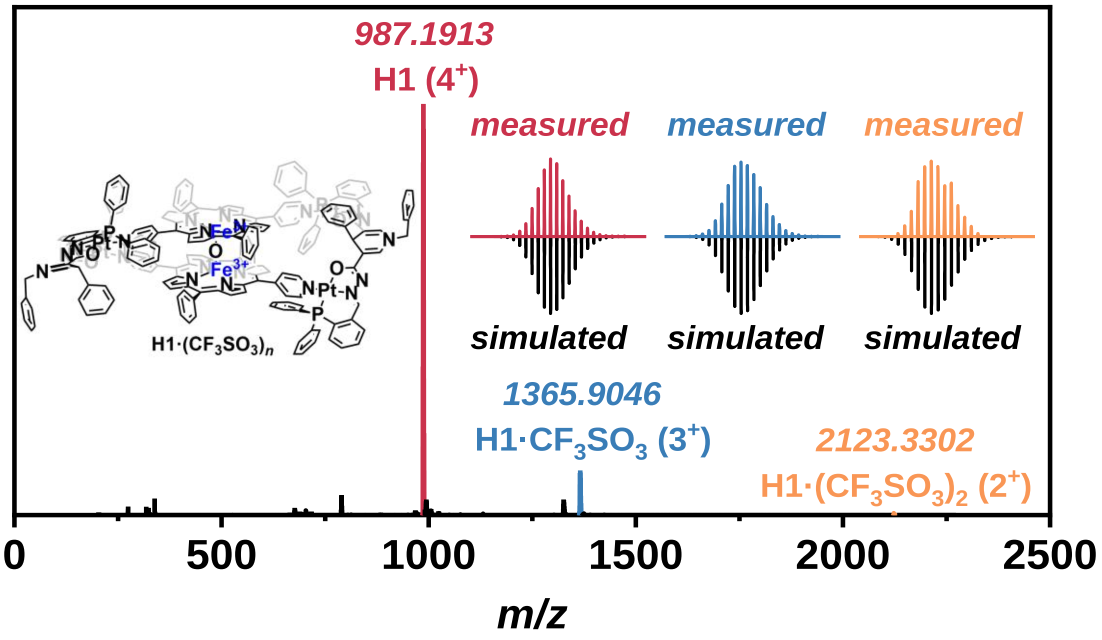

Peak	Value of *m/z*	Specie assigned
1	987.1913	$[Pt_4(\mathbf{L}^P)_2(\mathbf{L}^{Fe})_2O]^{4+}$
2	1365.9046	$[Pt_4(\mathbf{L}^P)_2(\mathbf{L}^{Fe})_2O(CF_3SO_3^-)]^{3+}$
3	2123.3302	$[Pt_4(\mathbf{L}^P)_2(\mathbf{L}^{Fe})_2O(CF_3SO_3^-)_2]^{2+}$

Figure S13. ESI-MS spectrum of **H1** (0.5 mM) in acetonitrile solution. The inset shows the measured and simulated isotopic patterns at *m/z* = 987.1913 (red), 1365.9046 (blue), and 2123.3302 (orange), corresponding to $[Pt_4(\mathbf{L}^P)_2(\mathbf{L}^{Fe})_2O(CF_3SO_3^-)_n]^{(4-n)+}$ (n = 0, 1, and 2), respectively.

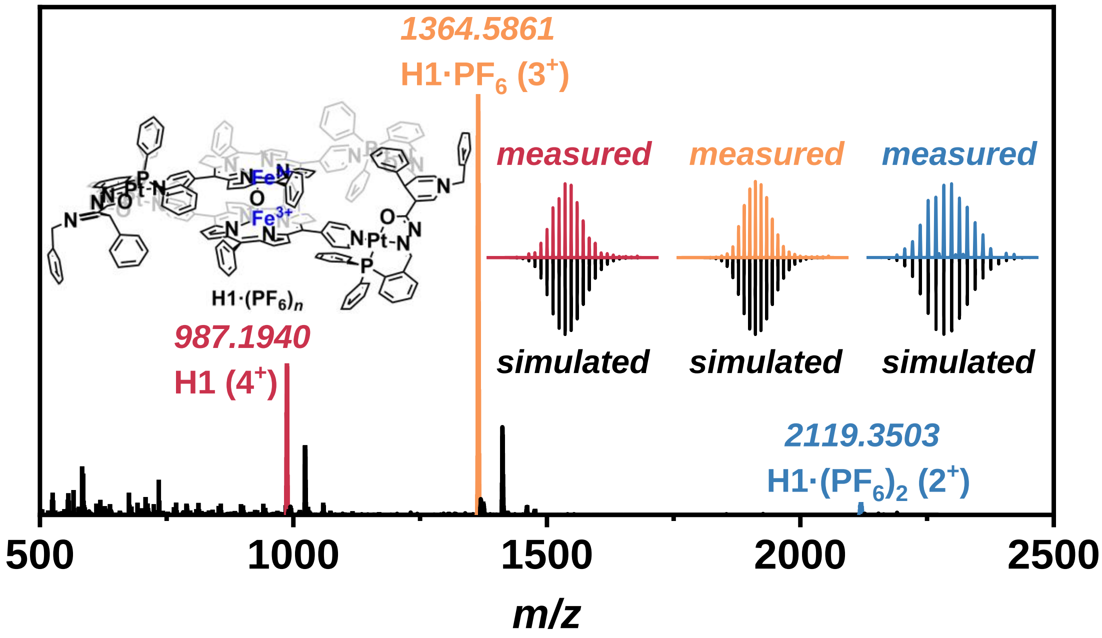

Peak	Value of *m/z*	Specie assigned
1	987.1940	$[Pt_4(\mathbf{L}^{P})_2(\mathbf{L}^{Fe})_2O]^{4+}$
2	1364.5861	$[Pt_4(\mathbf{L}^{P})_2(\mathbf{L}^{Fe})_2O(PF_6^{-})]^{3+}$
3	2119.3503	$[Pt_4(\mathbf{L}^{P})_2(\mathbf{L}^{Fe})_2O(PF_6^{-})_2]^{2+}$

Figure S14. ESI-MS spectrum of **H1** (0.5 mM) in acetonitrile solution after the exchange of anions to PF_6^{-}. The inset shows the measured and simulated isotopic patterns at *m/z* = 987.1940 (red), 1364.5861 (orange), and 2119.3503 (blue), corresponding to $[Pt_4(\mathbf{L}^{P})_2(\mathbf{L}^{Fe})_2O(PF_6^{-})_n]^{(4-n)+}$ (n = 0, 1, and 2), respectively.

3.2 ESI-MS spectra of the reduced capsule H_{Red} and the intermediates during the oxidation process

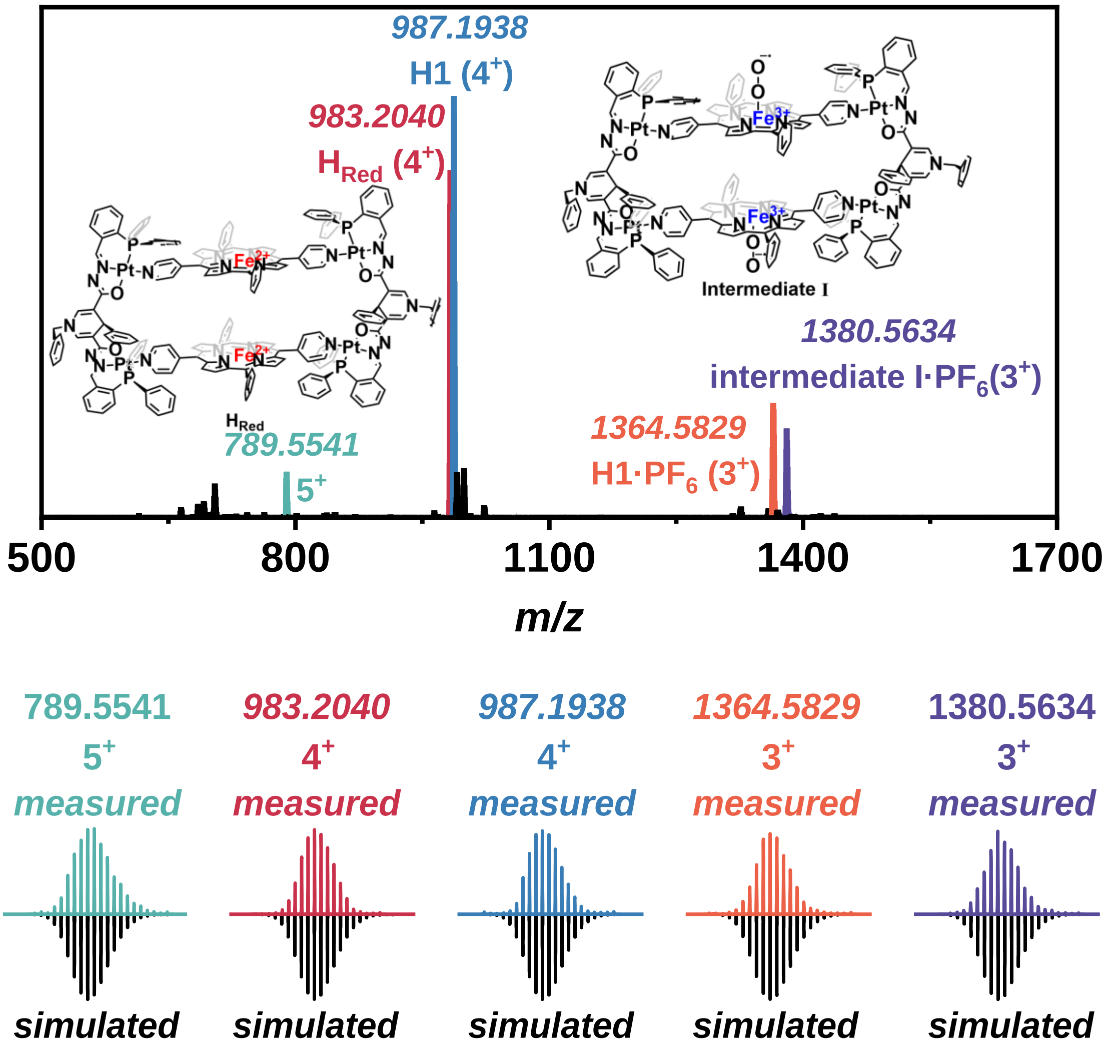

Figure S15. ESI-MS spectrum of **H1** (0.5 mM) after the reaction with $Na_2S_2O_4$ in acetonitrile solution. The inset shows the structures of $\mathbf{H_{Red}}$ and intermediate I, respectively. The measured and simulated isotopic patterns at *m/z* = 789.5541 (cyan), 983.2040 (red), 987.1938 (blue), 1364.5829 (orange), and 1380.5634 (purple) are shown below, respectively. The generation of $[Pt_4(\mathbf{L}^P)_2(\mathbf{L}^{Fe})_2(O_2)_2(PF_6^-)]^{3+}$ (intermediate I·PF_6) might be attributed to the introduction of a small amount of air during the preparation of the sample for ESI-MS.

Table S4. Corresponding species at *m/z* = 789.5541 (cyan), 983.2040 (red), 987.1938 (blue), 1364.5829 (orange), and 1380.5634 (purple), respectively ($\mathbf{L^{P}_{Ox}}$: oxidized $\mathbf{L^{P}}$).

Peak	Value of *m/z*	Specie assigned
1	789.5541	$[Pt_4(\mathbf{L^{P}})(\mathbf{L^{P}_{Ox}})(\mathbf{L^{Fe}})_2O]^{5+}$
2	983.2040	$[Pt_4(\mathbf{L^{P}})_2(\mathbf{L^{Fe}_{Red}})_2]^{4+}$
3	987.1938	$[Pt_4(\mathbf{L^{P}})_2(\mathbf{L^{Fe}})_2O]^{4+}$
4	1364.5829	$[Pt_4(\mathbf{L^{P}})_2(\mathbf{L^{Fe}})_2O(PF_6^{-})]^{3+}$
5	1380.5634	$[Pt_4(\mathbf{L^{P}})_2(\mathbf{L^{Fe}})_2(O_2)_2(PF_6^{-})]^{3+}$

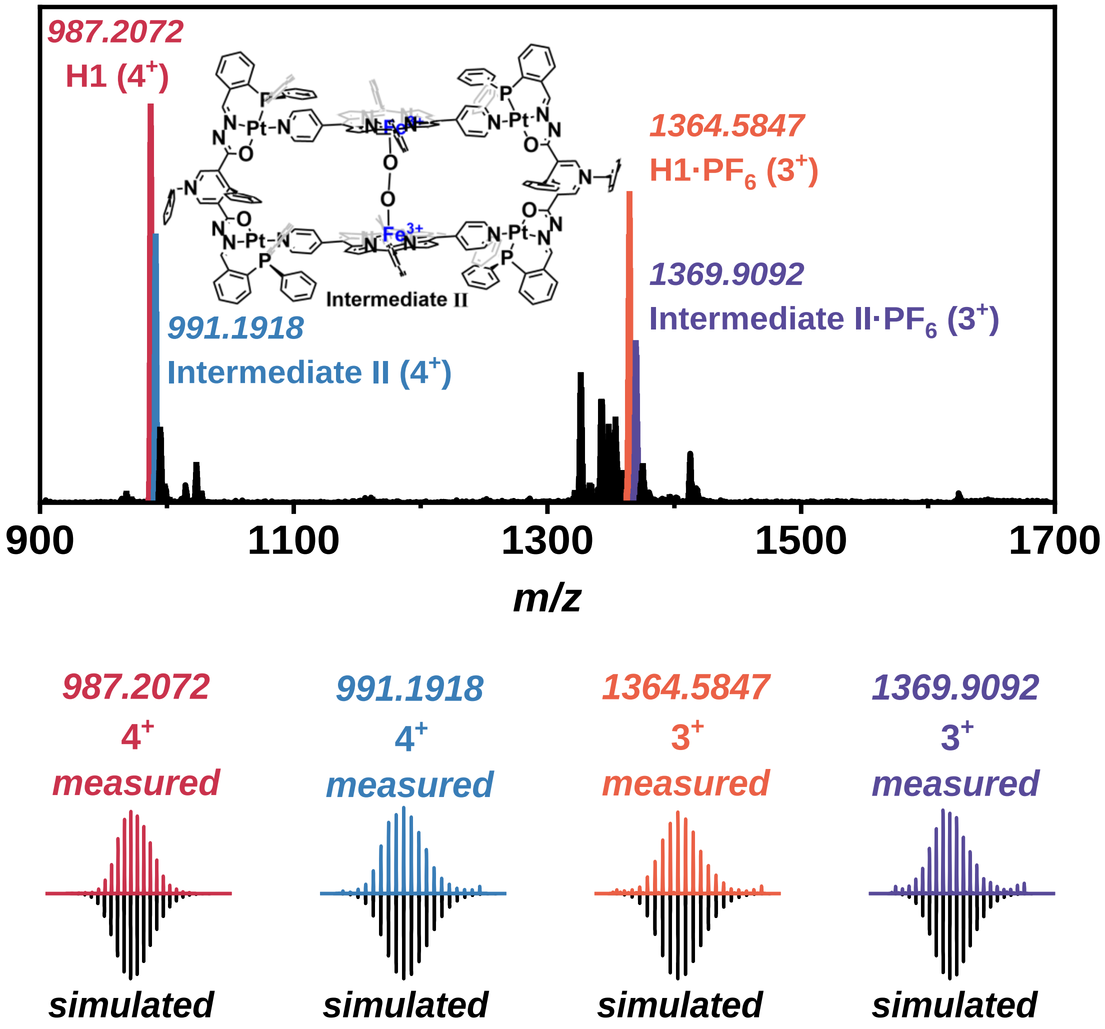

Figure S16. ESI-MS spectrum of **H1** (0.5 mM) after the reaction with $Na_2S_2O_4$ in acetonitrile solution in another sampling. The inset shows the structure of intermediate II. The measured and simulated isotopic patterns at *m/z* = 987.2072 (red), 991.1918 (blue), 1364.5847 (orange), and 1369.9092 (purple) are shown below, respectively. The production of $[Pt_4(\mathbf{L^P})_2(\mathbf{L^{Fe}})_2(O_2)]^{4+}$ (intermediate II) might be attributed to the inevitable introduction of trace amounts of air during the preparation of the ESI-MS sample.

Table S5. Corresponding species at *m/z* = 987.2072 (red), 991.1918 (blue), 1364.5847 (orange), and 1369.9092 (purple), respectively.

Peak	Value of *m/z*	Specie assigned
1	987.2072	$[Pt_4(\mathbf{L}^{\mathbf{P}})_2(\mathbf{L}^{\mathbf{Fe}})_2O]^{4+}$
2	991.1918	$[Pt_4(\mathbf{L}^{\mathbf{P}})_2(\mathbf{L}^{\mathbf{Fe}})_2(O_2)]^{4+}$
3	1364.5847	$[Pt_4(\mathbf{L}^{\mathbf{P}})_2(\mathbf{L}^{\mathbf{Fe}})_2O(PF_6^{-})]^{3+}$
4	1369.9092	$[Pt_4(\mathbf{L}^{\mathbf{P}})_2(\mathbf{L}^{\mathbf{Fe}})_2(O_2)(PF_6^{-})]^{3+}$

3.3 ESI-MS spectra of H1 with μ-O bridged or separated iron(III) porphyrins

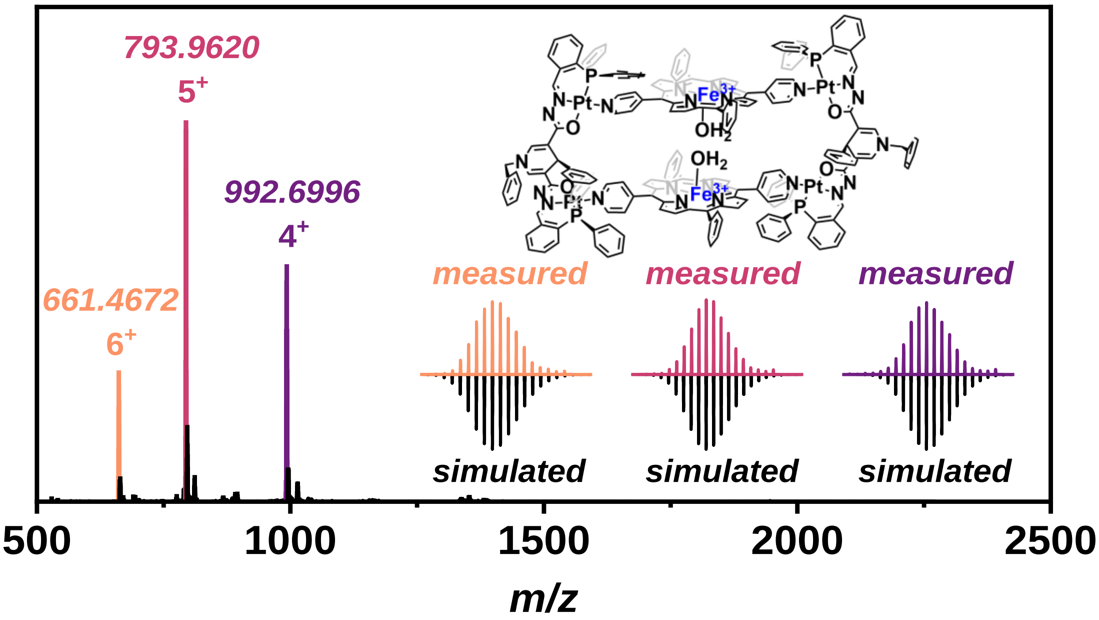

Peak	Value of *m/z*	Specie assigned
1	661.4672	$[Pt_4(\mathbf{L}^P)_2(\mathbf{L}^{Fe})_2(H_2O)_2]^{6+}$
2	793.9620	$[Pt_4(\mathbf{L}^P)_2(\mathbf{L}^{Fe})_2(H_2O)(F^-)]^{5+}$
3	992.6996	$[Pt_4(\mathbf{L}^P)_2(\mathbf{L}^{Fe})_2(F^-)_2]^{4+}$

Figure S17. ESI-MS spectrum of **H1** (0.5 mM) with the addition of HBF_4 (5.0 mM) in acetonitrile solution. The inset shows the measured and simulated isotopic patterns at *m/z* = 661.4672 (orange), 793.9620 (red), and 992.6996 (purple), corresponding to $[Pt_4(\mathbf{L}^P)_2(\mathbf{L}^{Fe})_2(H_2O)_n(F^-)_{(2-n)}]^{(4+n)+}$ (n = 2, 1, and 0), respectively.[8,9]

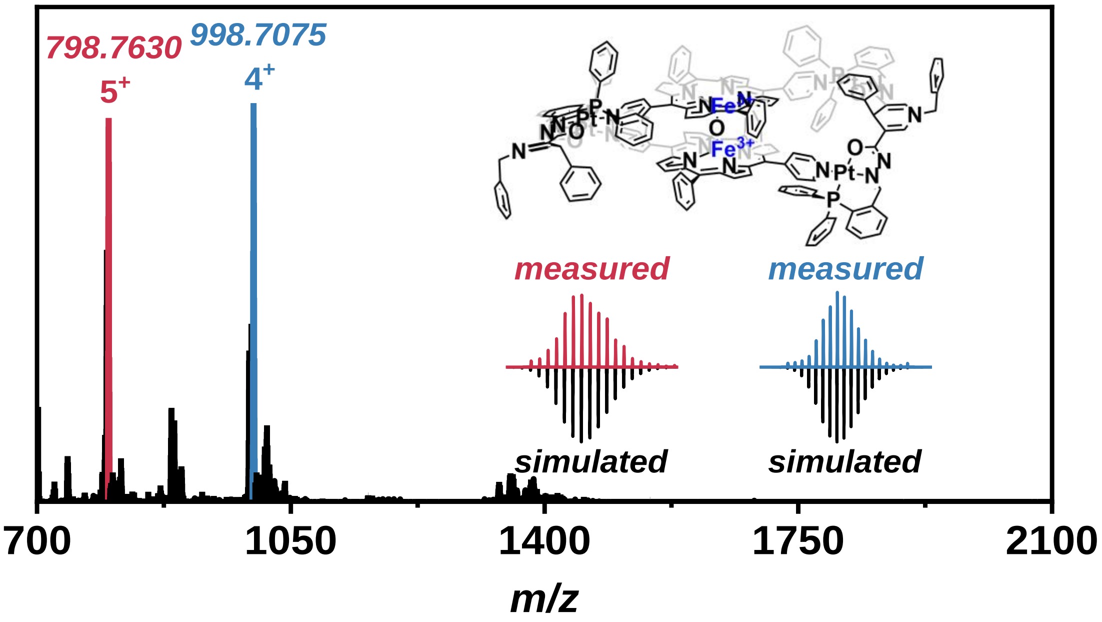

Peak	Value of *m/z*	Specie assigned
1	798.7630	$[Pt_4(\mathbf{L^P})(\mathbf{L^P}_{Ox})(\mathbf{L^{Fe}})_2O(EtOH)]^{5+}$
2	998.7075	$[Pt_4(\mathbf{L^P})_2(\mathbf{L^{Fe}})_2O(EtOH)]^{4+}$

Figure S18. ESI-MS spectrum of **H1** (0.5 mM) with the addition of NaOH (5.0 mM) after the reaction with HBF_4 (5.0 mM) in acetonitrile solution. The inset shows the measured and simulated isotopic patterns at *m/z* = 798.7630 (red), and 998.7075 (blue), corresponding to $[Pt_4(\mathbf{L^P})(\mathbf{L^P}_{Ox})(\mathbf{L^{Fe}})_2O(EtOH)]^{5+}$ and $[Pt_4(\mathbf{L^P})_2(\mathbf{L^{Fe}})_2O(EtOH)]^{4+}$, respectively.

4. UV-vis and kinetics experiments data of the oxidation of substrates by H1

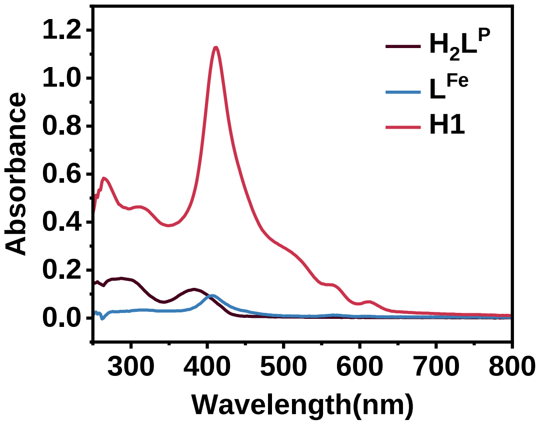

Figure S19. UV-vis absorption spectra of ligand $H_2\mathbf{L}^{\mathbf{P}}$ (5.0 μM), ligand $\mathbf{L}^{\mathbf{Fe}}$ (5.0 μM), and **H1** (5.0 μM) in DMF solution.

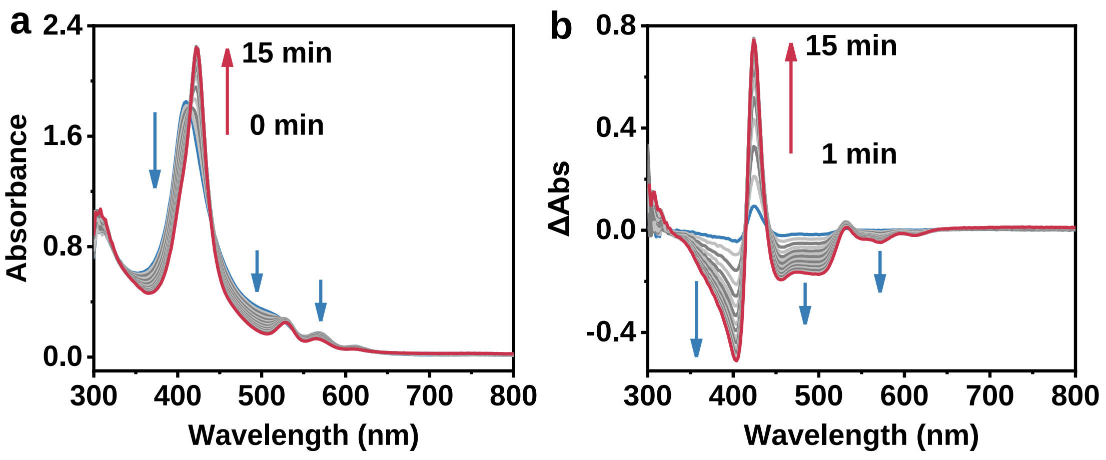

Figure S20. (a) Family of UV-vis absorption spectra of **H1** (10.0 μM) and **1a** (0.025 M) in anaerobic pyridine upon 420 nm LED irradiation for different times. (b) The differential spectra as a function of time. Spectra were recorded over 15 min at 1 min intervals.

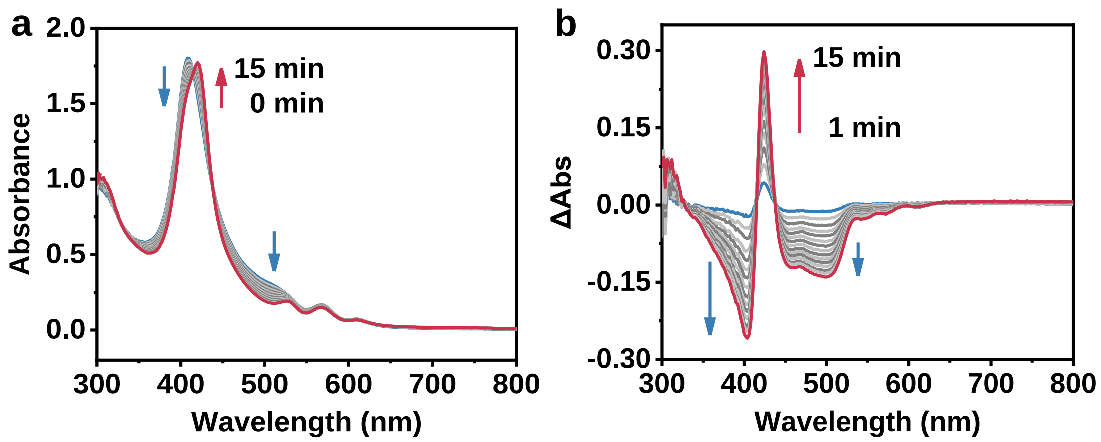

Figure S21. (a) Family of UV-vis absorption spectra of **H1** (10.0 μM) in anaerobic pyridine upon 420 nm LED irradiation for different times. (b) The differential spectra as a function of time. Spectra were recorded over 15 min at 1 min intervals.

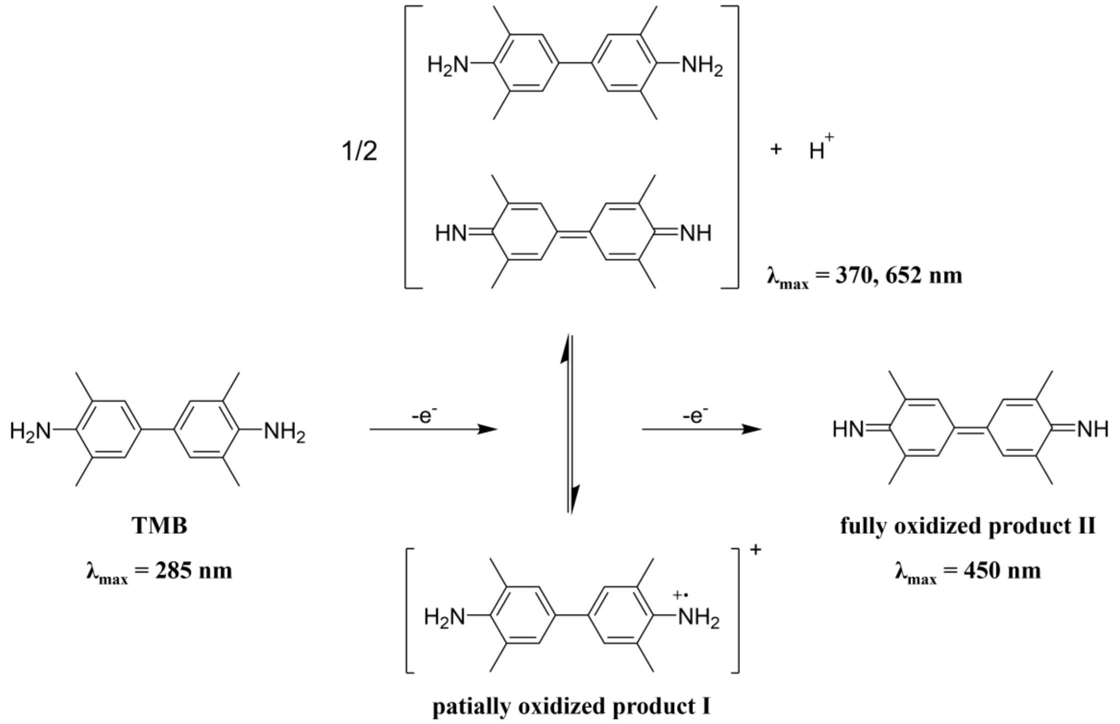

Figure S22. The oxidation products of TMB, through one-electron and two-electron oxidation processes, respectively.

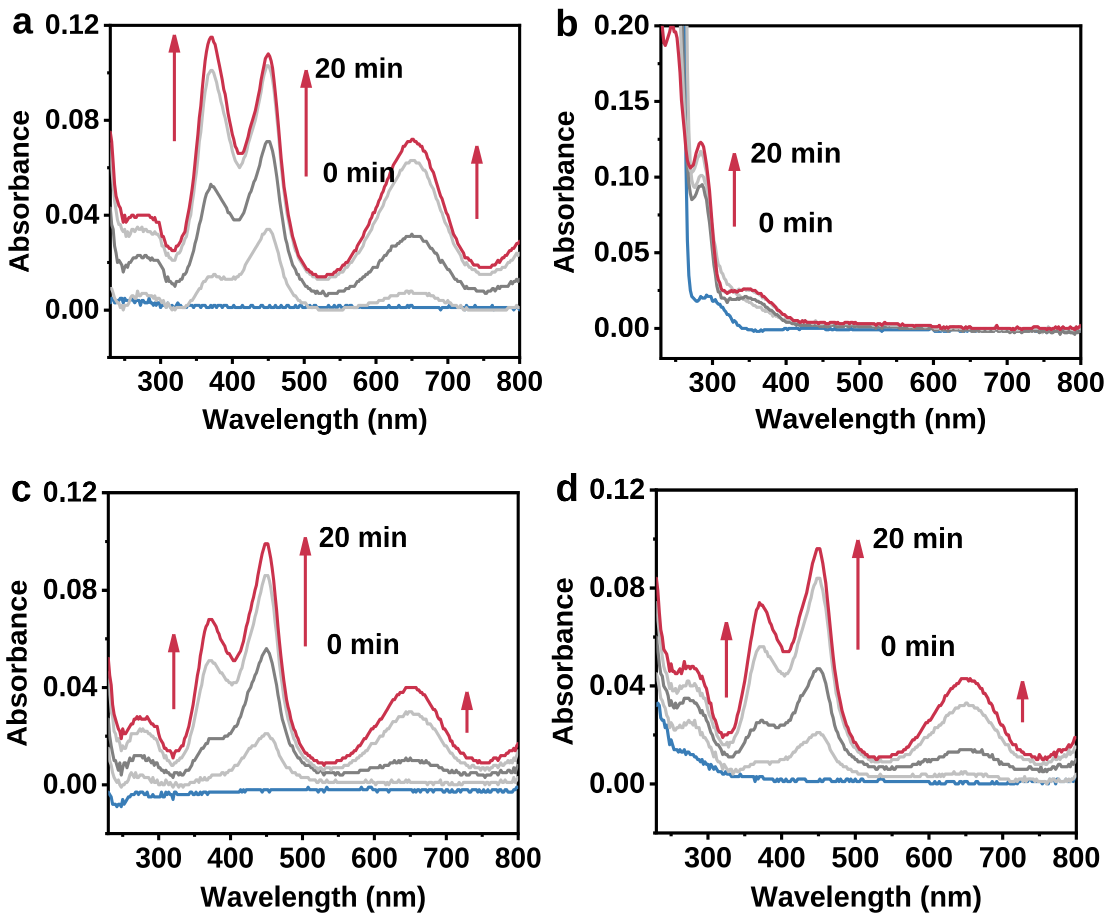

Figure S23. UV-vis absorption spectra of TMB oxidized by **H1** under the standard condition (a) and in the presence of 1,4-*p*-benzoquinone (b), DABCO (c), and tert-butanol (d) upon 420 nm LED irradiation for different times, respectively. Spectra were recorded over the span of 20 min at 5 min intervals.

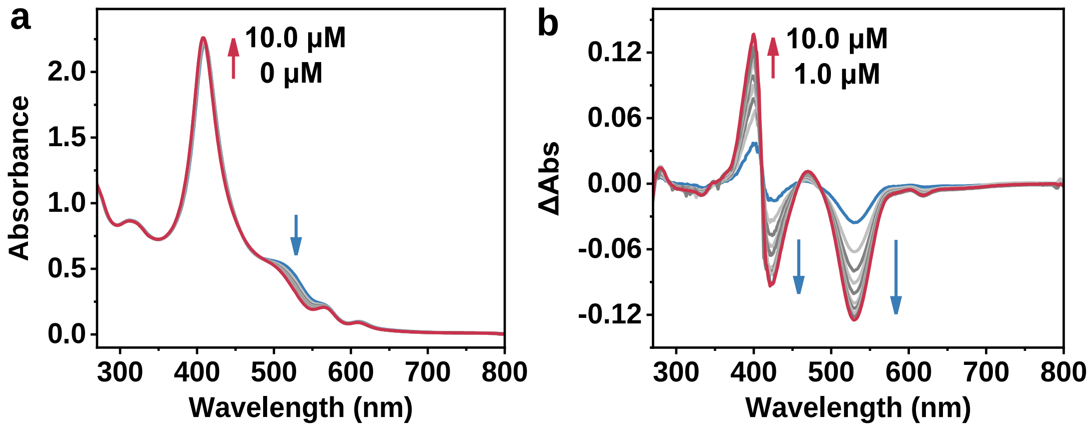

Figure S24. (a) Family of UV-vis absorption spectra of **H1** (10.0 μM) in MeOH/H_2O (1:1, v/v) solution upon the addition of **1a** (total 10.0 μM) with various concentrations. (b) The differential spectra as a function of the concentration of **1a**. Spectra were recorded throughout 10.0 μM at 1.0 μM intervals.

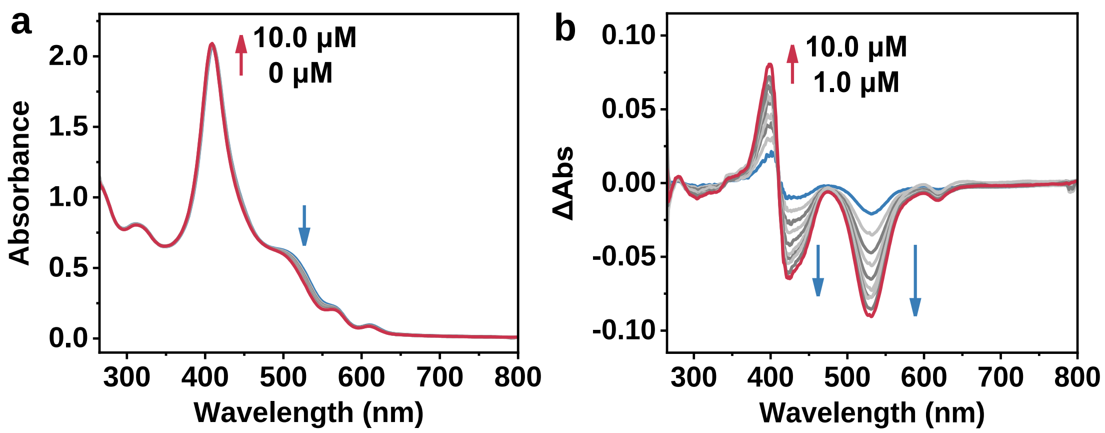

Figure S25. (a) Family of UV-vis absorption spectra of **H1** (10.0 μM) in MeOH/H_2O (1:1, v/v) solution upon the addition of Met (total 10.0 μM) with various concentrations. (b) The differential spectra as a function of the concentration of Met. Spectra were recorded throughout 10.0 μM at 1.0 μM intervals.

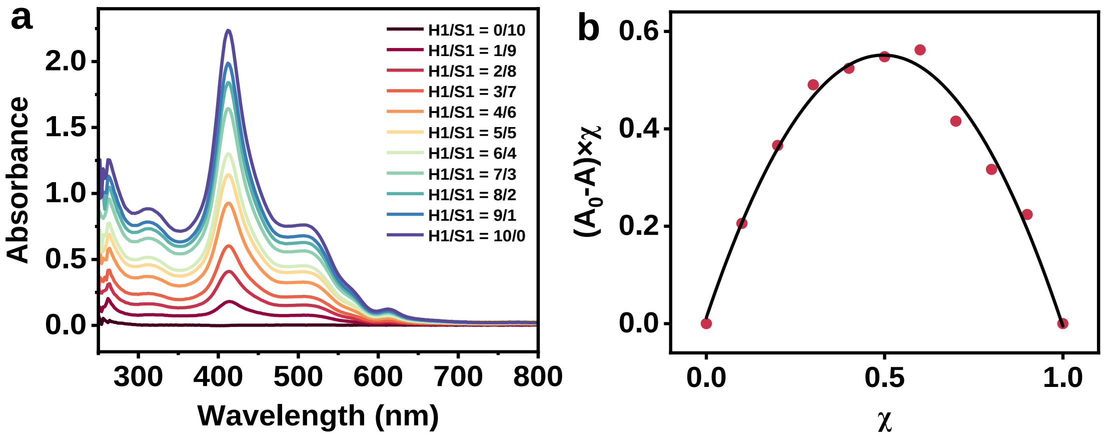

Figure S26. (a) UV-vis difference spectrometry of **1a** inclusion in MeOH/H_2O (1:1, v/v). (b) Job's plot of absorbance changes at 412 nm.

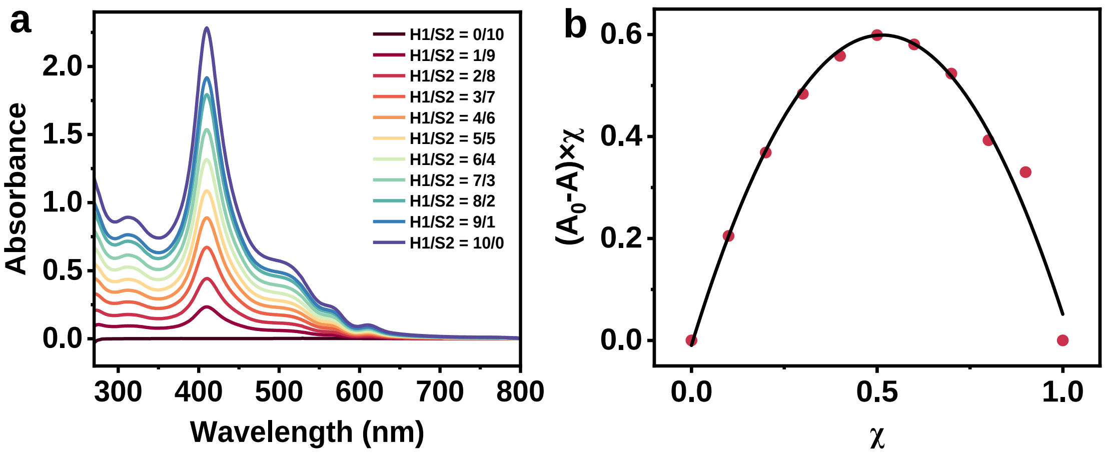

Figure S27. (a) UV-vis difference spectrometry of Met inclusion in MeOH/H_2O (1:1, v/v). (b) Job's plot of absorbance changes at 412 nm.

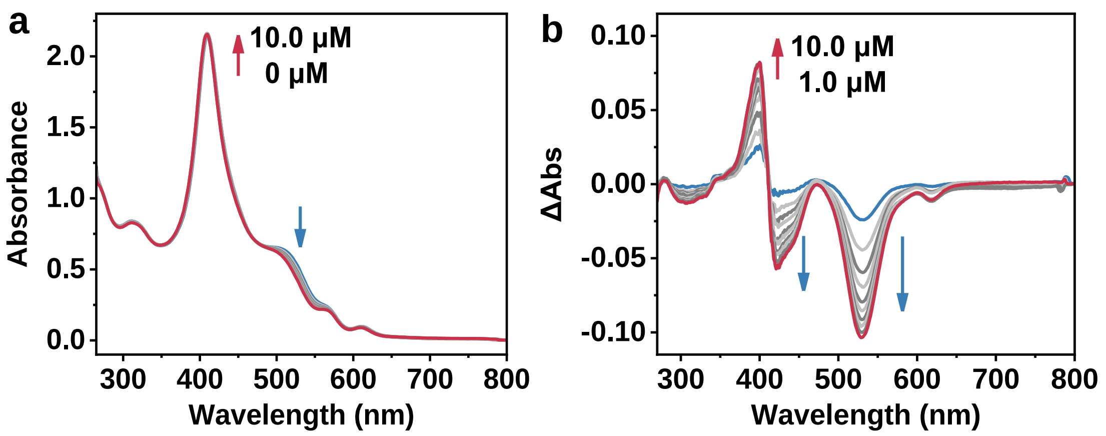

Figure S28. (a) Family of UV-vis absorption spectra of **H1** (10.0 μM) in MeOH/H_2O (1:1, v/v) solution upon the addition of **1b** (total 10.0 μM) with various concentrations. (b) The differential spectra as a function of the concentration of **1b**. Spectra were recorded throughout 10.0 μM at 1.0 μM intervals.

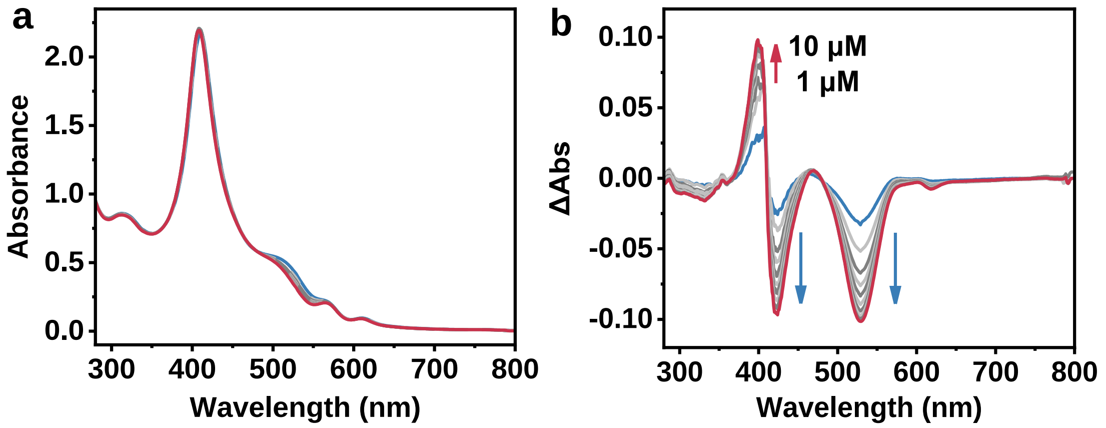

Figure S29. (a) Family of UV-vis absorption spectra of **H1** (10.0 μM) in MeOH/H_2O (1:1, v/v) solution upon the addition of MetO (total 10.0 μM) with various concentrations. (b) The differential spectra as a function of the concentration of MetO. Spectra were recorded throughout 10.0 μM at 1.0 μM intervals.

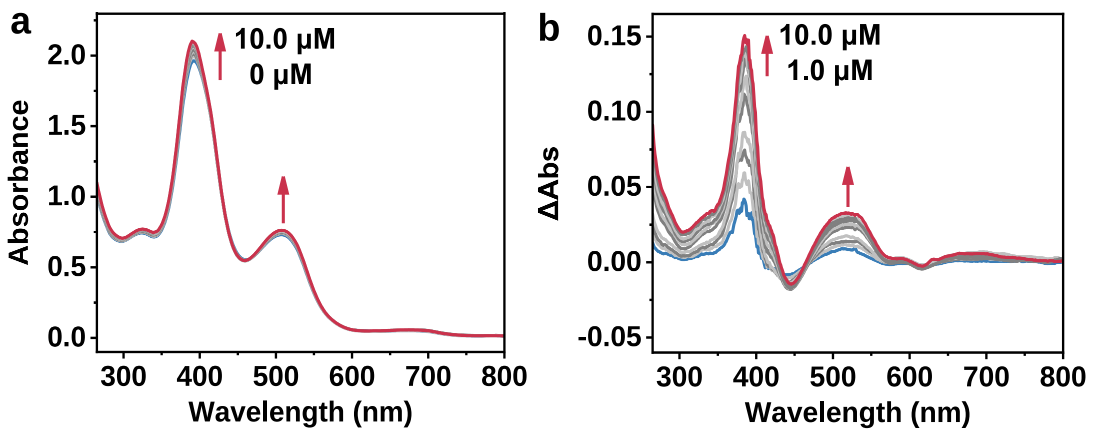

Figure S30. (a) Family of UV-vis absorption spectra of **H1** (10.0 μM) with HBF_4 (30.0 μM) in MeOH/H_2O (1:1, v/v) solution upon the addition of **1a** (total 10.0 μM) with various concentrations. (b) The differential spectra as a function of the concentration of **1a**. Spectra were recorded throughout 10.0 μM at 1.0 μM intervals.

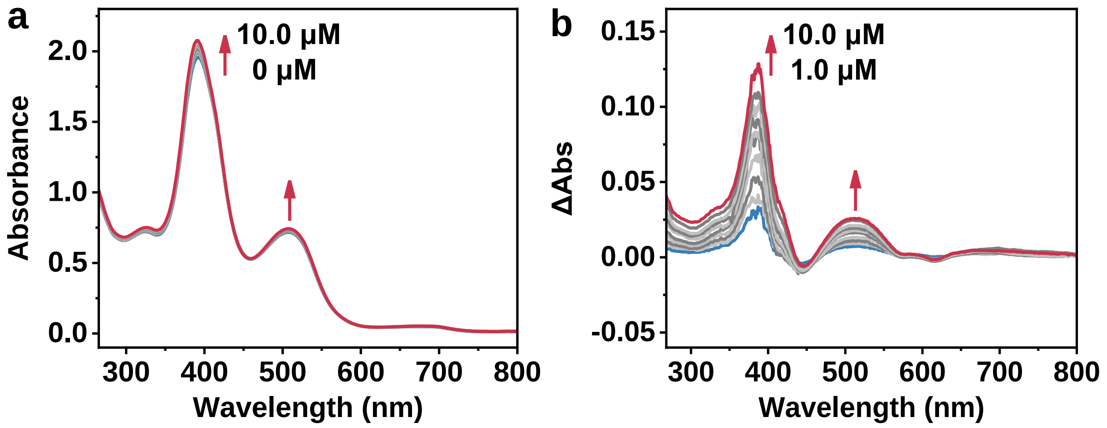

Figure S31. (a) Family of UV-vis absorption spectra of **H1** (10.0 μM) with HBF_4 (30.0 μM) in MeOH/H_2O (1:1, v/v) solution upon the addition of Met (total 10.0 μM) with various concentrations. (b) The differential spectra as a function of the concentration of Met. Spectra were recorded throughout 10.0 μM at 1.0 μM intervals.

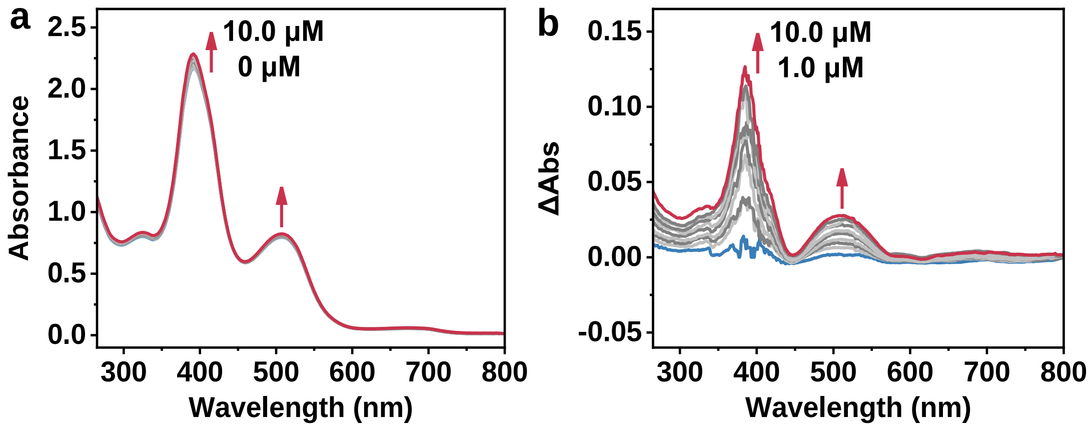

Figure S32. (a) Family of UV-vis absorption spectra of **H1** (10.0 µM) with HBF_4 (30.0 µM) in MeOH/H_2O (1:1, v/v) solution upon the addition of **1b** (total 10.0 µM) with various concentrations. (b) The differential spectra as a function of the concentration of **1b**. Spectra were recorded throughout 10.0 µM at 1.0 µM intervals.

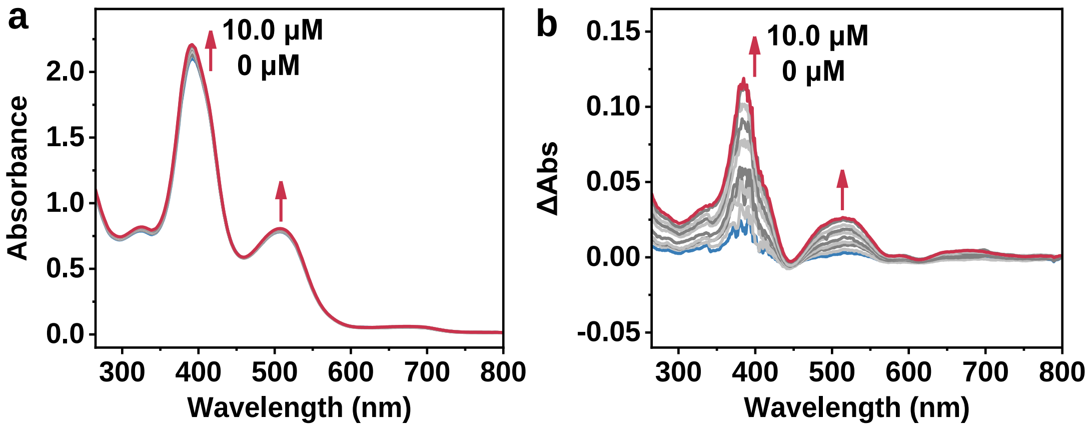

Figure S33. (a) Family of UV-vis absorption spectra of **H1** (10.0 μM) with HBF_4 (30.0 μM) in MeOH/H_2O (1:1, v/v) solution upon the addition of MetO (total 10.0 μM) with various concentrations. (b) The differential spectra as a function of the concentration of MetO. Spectra were recorded throughout 10.0 μM at 1.0 μM intervals.

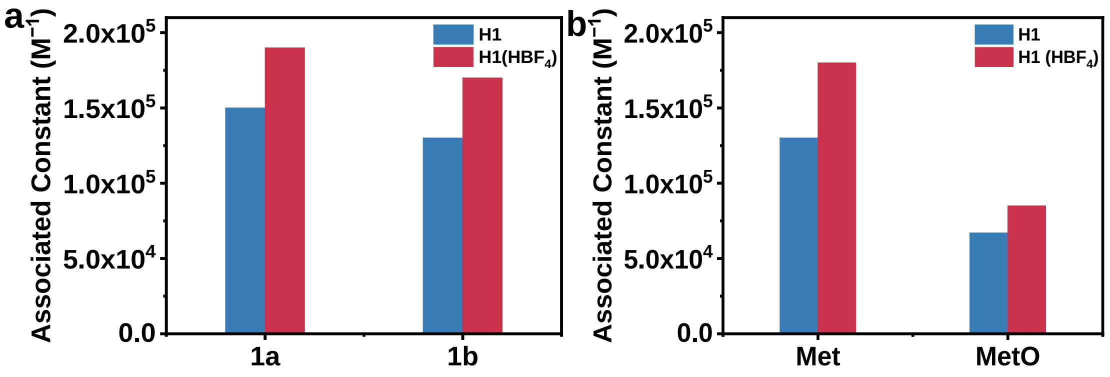

Figure S34. Associated constants obtained from Hill plot fitting of titration of **1a**/**1b** (a), Met/MetO (b), and **H1** in MeOH/H_2O (1:1, v/v) solution, regardless of the presence of HBF_4.

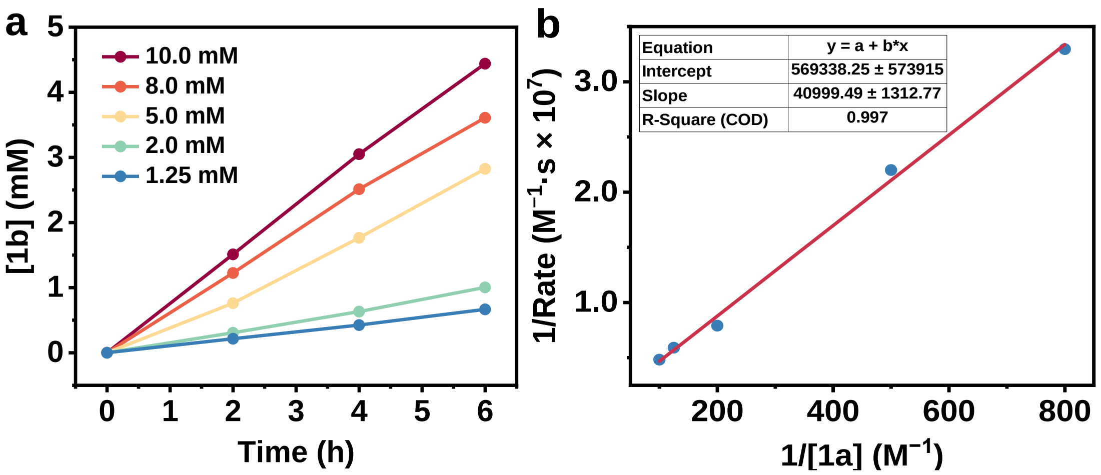

Figure S35. (a) Kinetics experiments of the oxidation of **1a** by **H1** with different concentrations of **1a** ([**1b**] = the concentration of methyl phenyl sulfoxide). (b) The reciprocal (Lineweaver-Burk) plot of the initial rate of **1a** oxidation by **H1** and the concentrations of **1a** suggested a Michaelis-Menten mechanism involving the catalytic process ([**1a**] = the concentration of thioanisole).

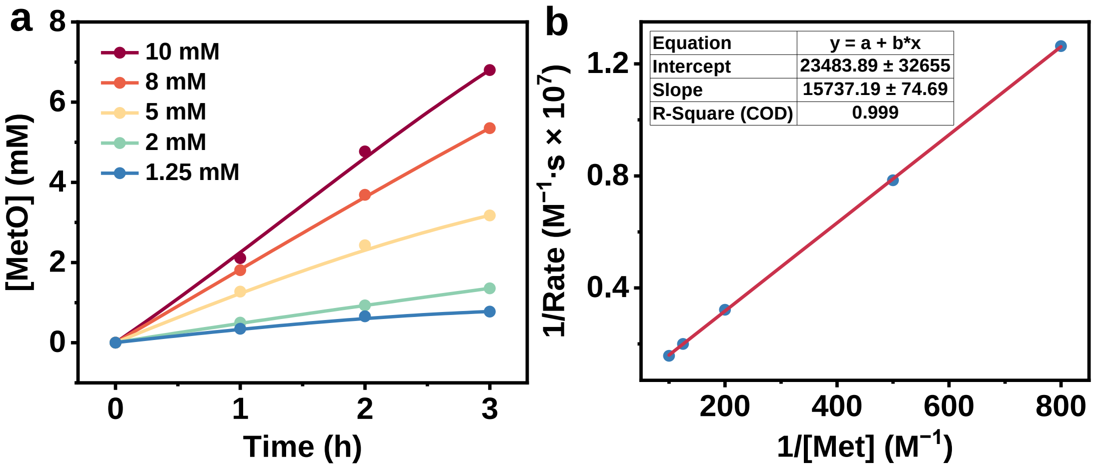

Figure S36. (a) Kinetics experiments of the oxidation of Met by **H1** with different concentrations of Met ([MetO] = the concentration of methionine sulfoxide). (b) The reciprocal (Lineweaver-Burk) plot of the initial rate of Met oxidation by **H1** and the concentrations of Met suggested a Michaelis-Menten mechanism involving the catalytic process ([Met] = the concentration of methionine).

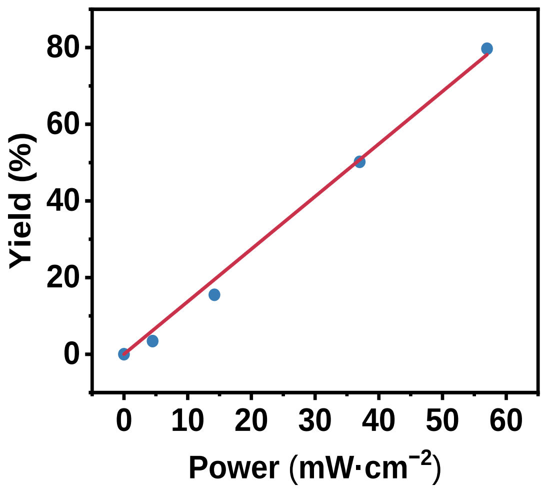

Figure S37. The yields of the oxidation reaction of **1a** as a function of irradiation powers under standard conditions upon 420 nm LED irradiation within 6 h showed a linear relationship between the yields of photocatalysis and the photon powers.

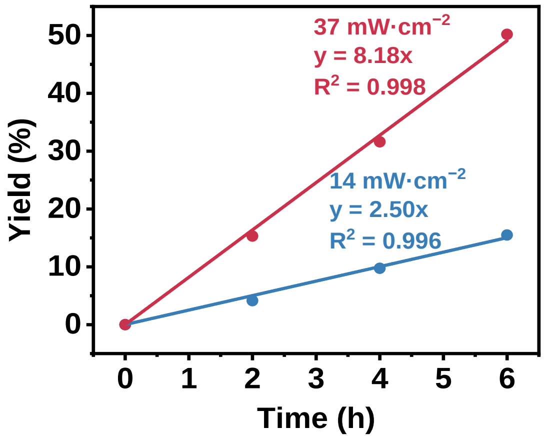

Figure S38. The yields of the oxidation reaction of **1a** as a function of time under standard conditions upon 420 nm LED irradiation of 14 and 37 ($mW \cdot cm^{-2}$) within 6 h, respectively.

Table S6. Oxidation of Met catalyzed by **H1** under different conditions.

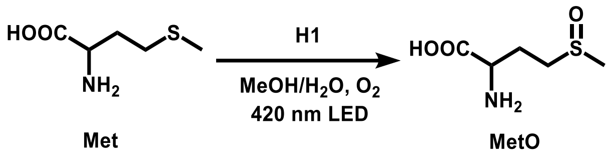

Entry	Conditions[a)]	Yield[c)] [%]
1	Standard	67.3
2	No **H1**	8.1
3	No light	trace
4[b)]	Addition of HBF_4	27.3
5	No **H1** and light	trace
6[b)]	No light	trace

[a)]Standard conditions: **H1** (0.1 mM) and Met (2.0 mM) in MeOH/H_2O (1:1, v/v, 2.0 mL) with irradiation upon 420 nm LED under an O_2 atmosphere at 25°C for 6 h; [b)]2.0 mM HBF_4; [c)]Yields of MetO were determined by HPLC.

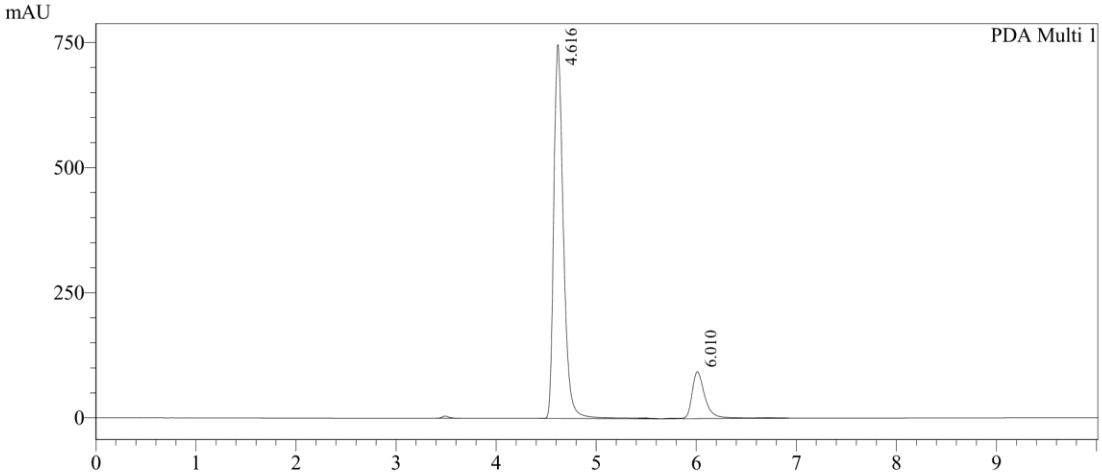

Figure S39. The HPLC spectrum of MetO (4.62 min) and Met (6.01 min) in the mixture.

5. UV-vis titration experiments data of the transformation between *μ*-O bridged and separated iron(III) porphyrins on H1

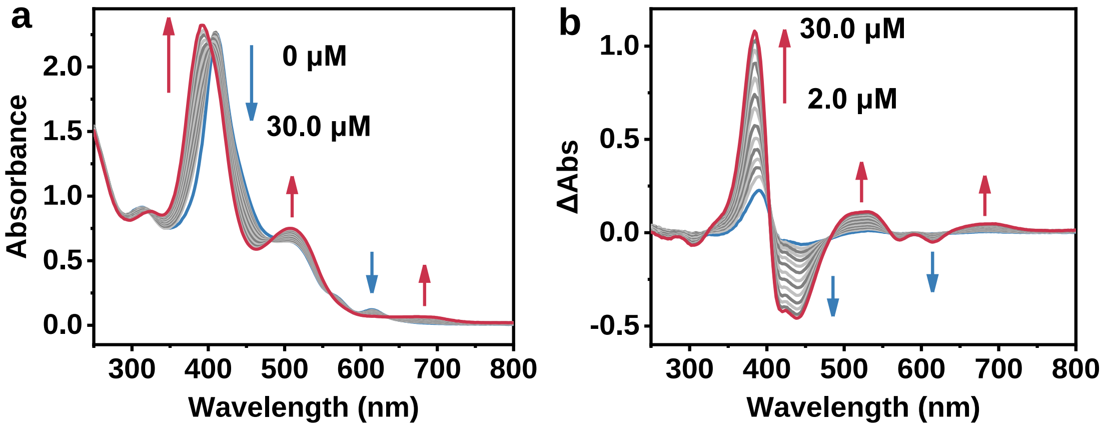

Figure S40. (a) Family of UV-vis absorption spectra of **H1** (10.0 μM) in methanol solution upon the addition of HBF_4 (total 30.0 μM). (b) The differential spectra as a function of the concentration of HBF_4.

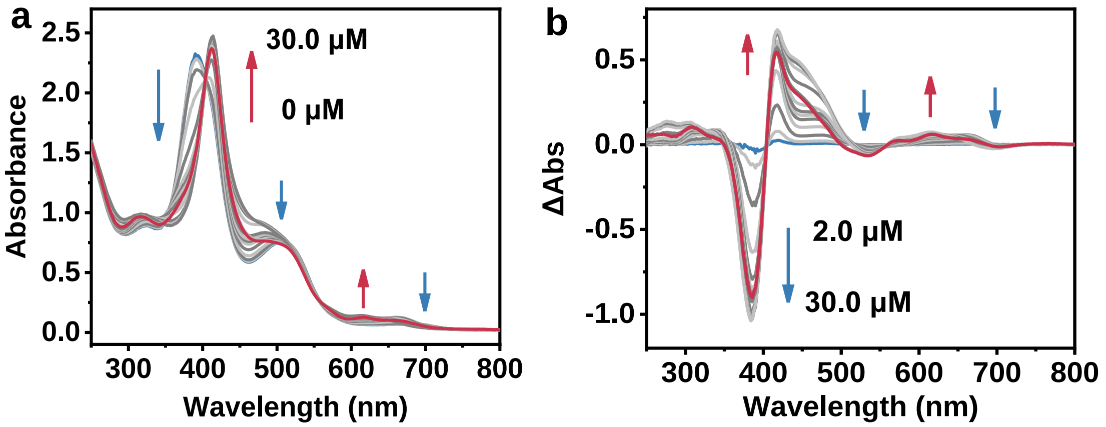

Figure S41. (a) Family of UV-vis absorption spectra of **H1** (10.0 μM) in methanol solution upon the addition of NaOH (total 30.0 μM) after the addition of HBF_4 (total 30.0 μM). (b) The differential spectra as a function of the concentration of NaOH.

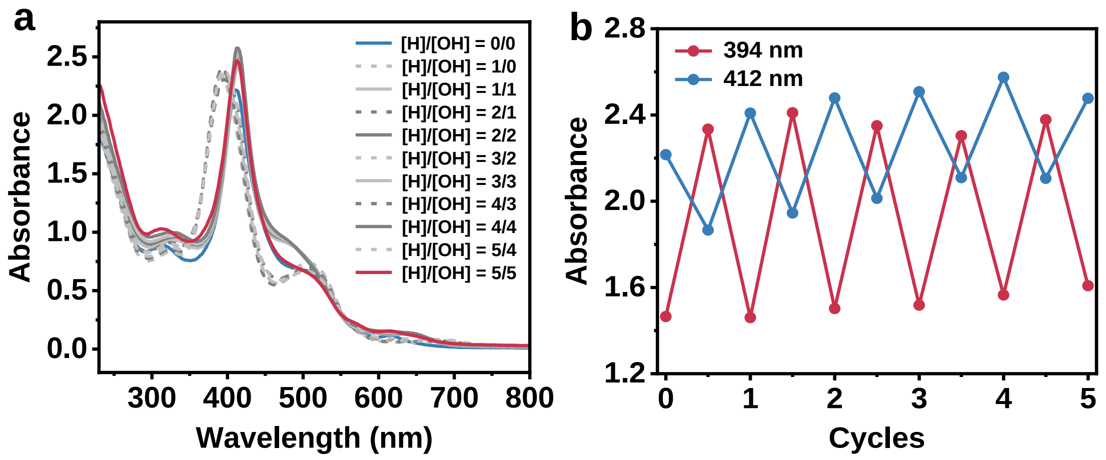

Figure S42. (a) UV-vis absorption spectra of capsule **H1** containing the μ-O bridged iron(III) porphyrins (dashed lines) and capsule containing separated iron(III) porphyrins (solid lines) in methanol solution after five cycles of sequential addition of HBF_4 (30.0 μM) and NaOH (30.0 μM) (initial [**H1**] = 10.0 μM). (b) Absorbance at 394 nm (red) and 412 nm (blue) of **H1** (10.0 μM) was monitored in five cycles of the transformation between μ-O bridged and separated iron(III) porphyrins on **H1** ([H] = [HBF_4], [OH] = [NaOH]).

6. Proposed mechanism of the oxidation reactions

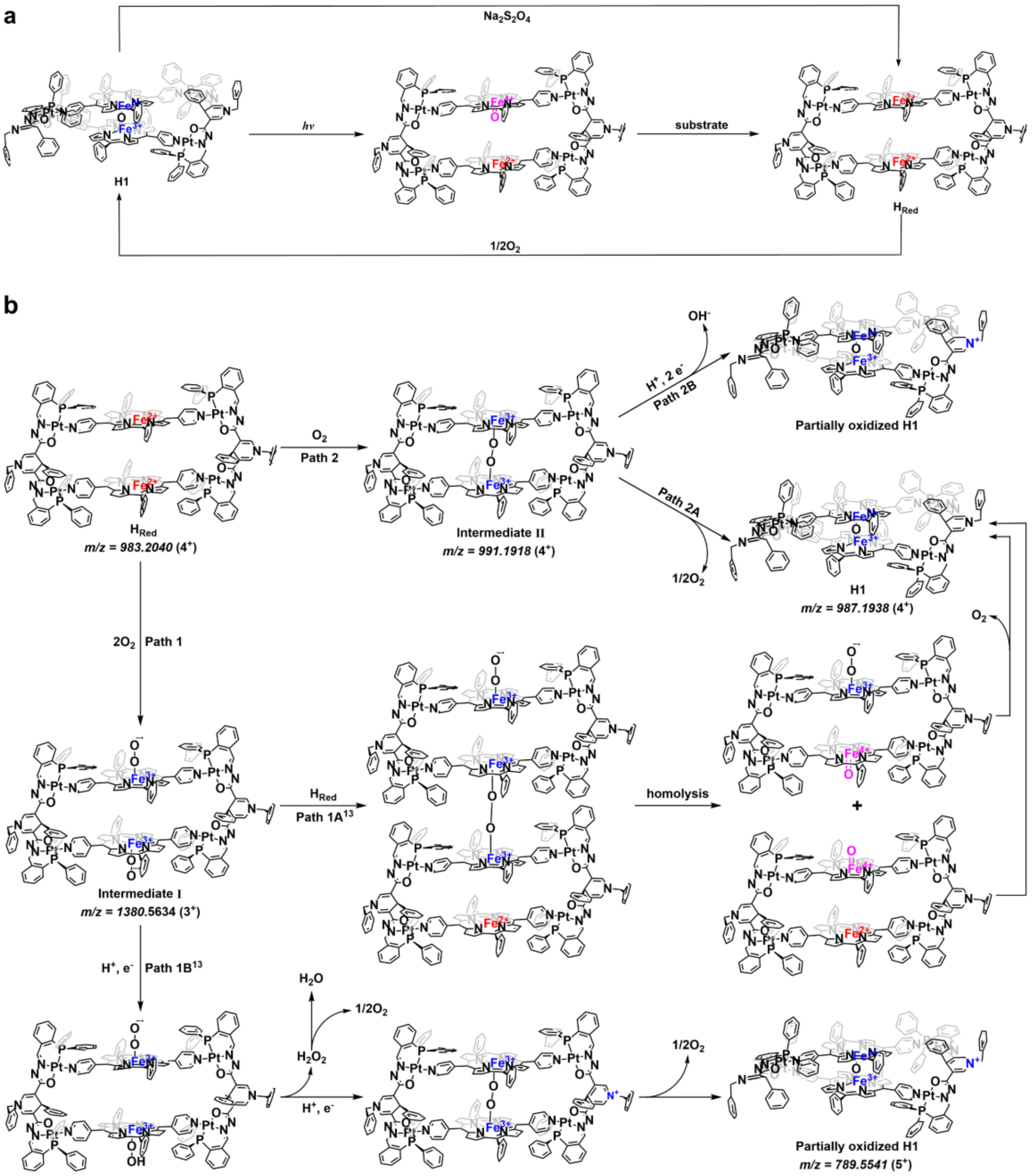

Figure S43. (a) The substrate oxidation mechanism of **H1** upon light irradiation. (b) Proposed mechanism for the conversion of the reduced capsule $\mathbf{H_{Red}}$ to the initial capsule **H1** in the presence of oxygen.[10]

7. In vitro and in vivo experiments

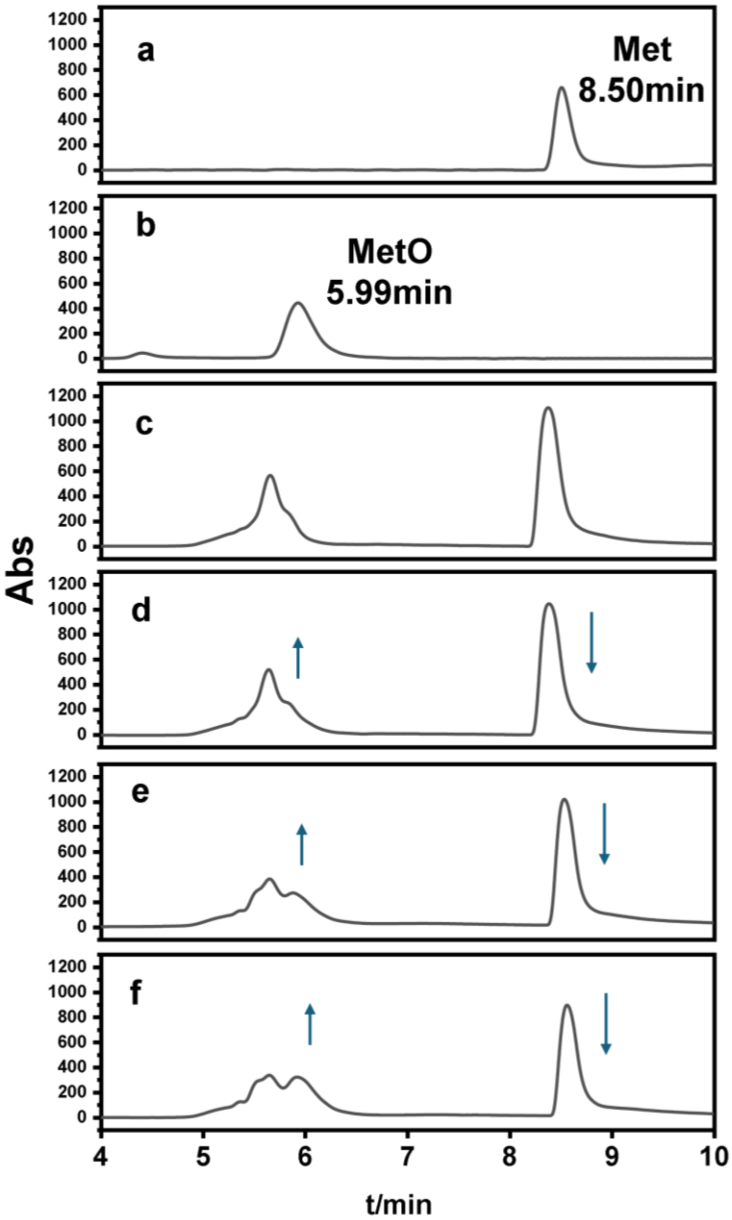

Figure S44. The catalytic oxidation behavior of intracellular **H1** toward methionine substrate. (a) Met standard sample, (b) MetO standard sample, (c) **H1**, (d) **H1**&irradiation for 90 min, (e) **H1**&$NaHCO_3$&irradiation for 45 min, (f) **H1**&$NaHCO_3$&irradiation for 90 min.

Table S7. Pt concentrations and CPS.

Pt concentrations [ppb]	CPS	CPS RSD
0	2102.516667	8.690675
10	75121.15	0.775585
20	152043.5233	0.603523
50	385227.9067	1.543532
100	795176.37	0.463056
200	1346302.35	0.976986
500	3388764.87	0.338812
1000	6565742.437	0.648068
sample	878684.4733	0.587011

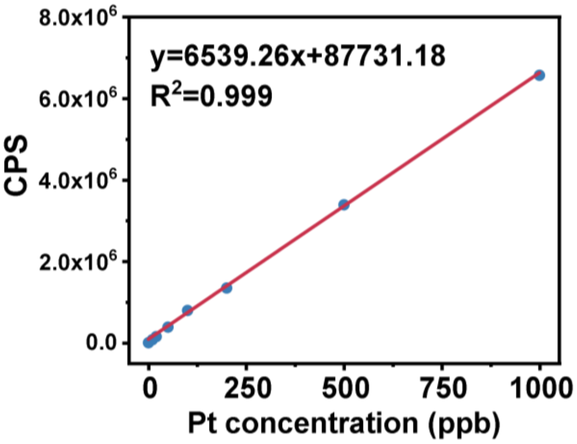

Figure S45. Standard curve of Pt concentration.

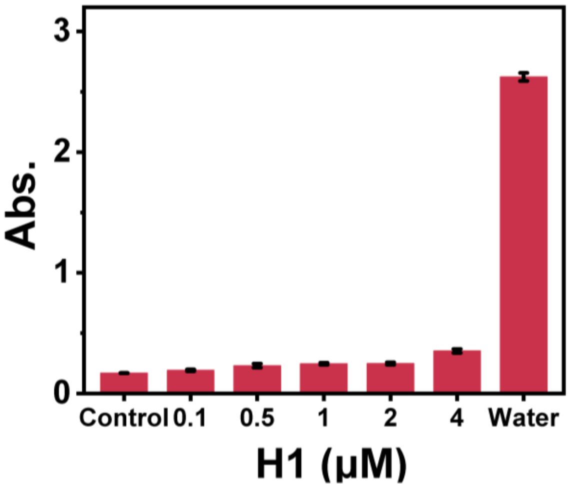

Figure S46. Hemolysis after incubation with different concentrations of **H1**.

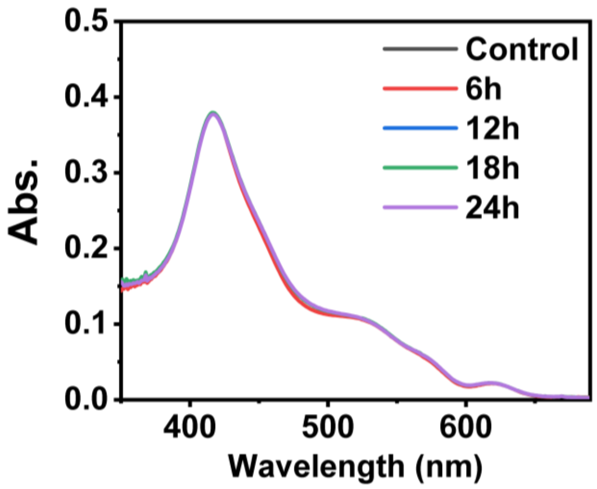

Figure S47. Stability of **H1** within 24 h in PBS containing 10% FBS.

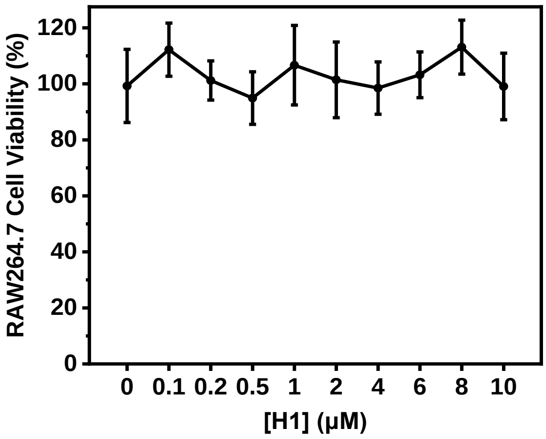

Figure S48. Cell viability of RAW264.7 cells under different concentrations of capsule **H1** under dark conditions.

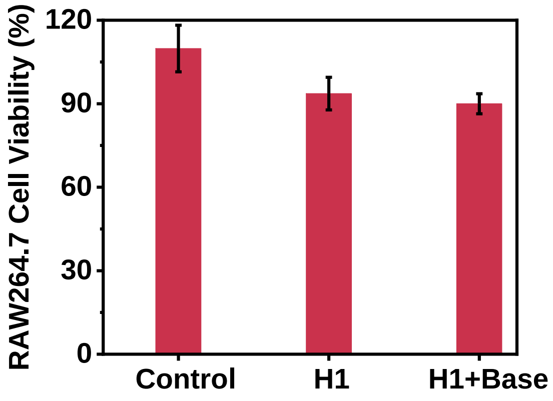

Figure S49. Cell activity of RAW264.7 cells under different conditions (400 to 830 nm LED light irradiation).

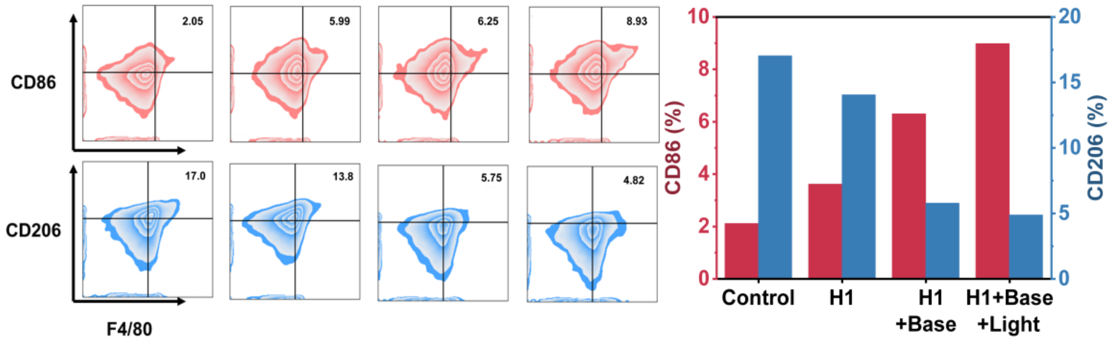

Figure S50. Polarization of RAW264.7 cells under different incubation conditions in conditioned media (from left to right: blank control, 2.0 μM **H1**, 2.0 μM **H1**&$NaHCO_3$, 2.0 μM **H1**&$NaHCO_3$&light) and the difference in the visualization of polarization of RAW264.7 cells under different incubation conditions. All flow cytometry experiments were repeated three times independently with similar results.

8. NMR spectra data

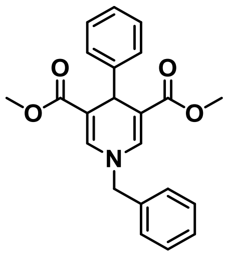

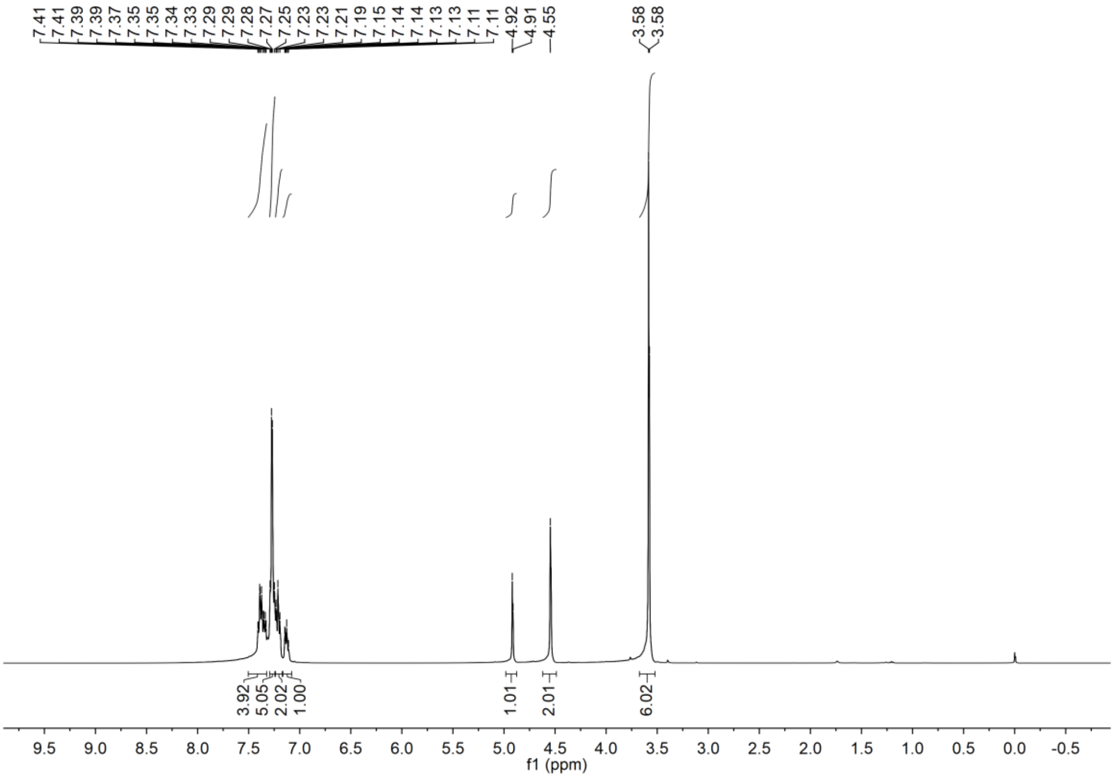

Figure S51. ^{1}H NMR spectrum of compound **1** in $CDCl_3$ at 298 K.

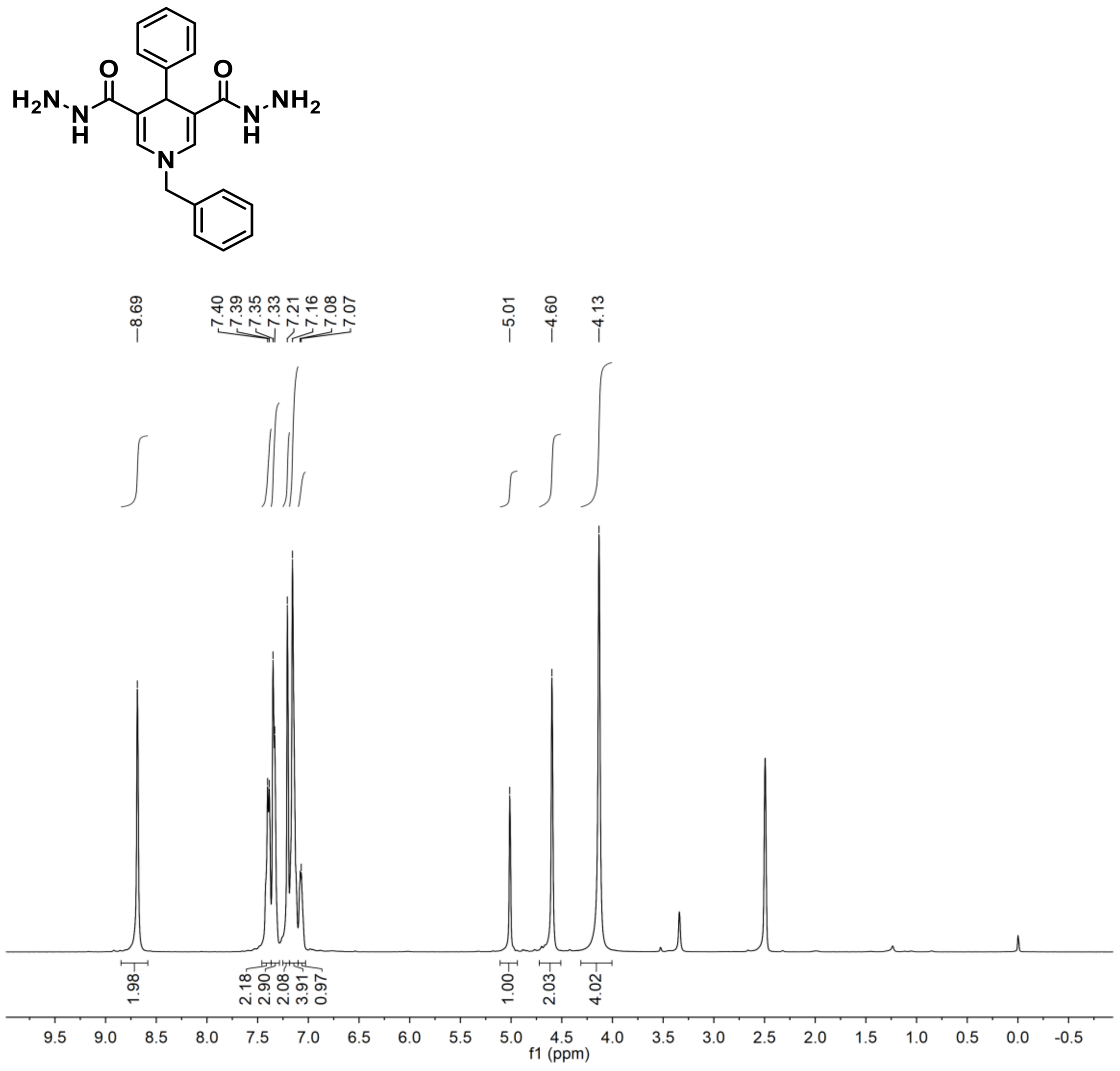

Figure S52. ^{1}H NMR spectrum of compound **2** in DMSO-d_6 at 298 K.

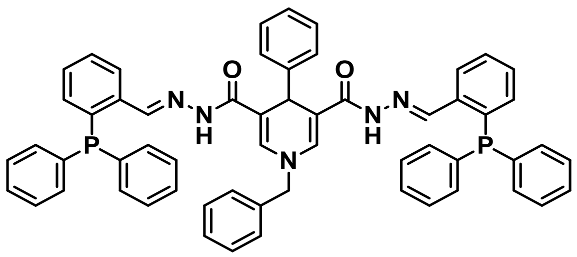

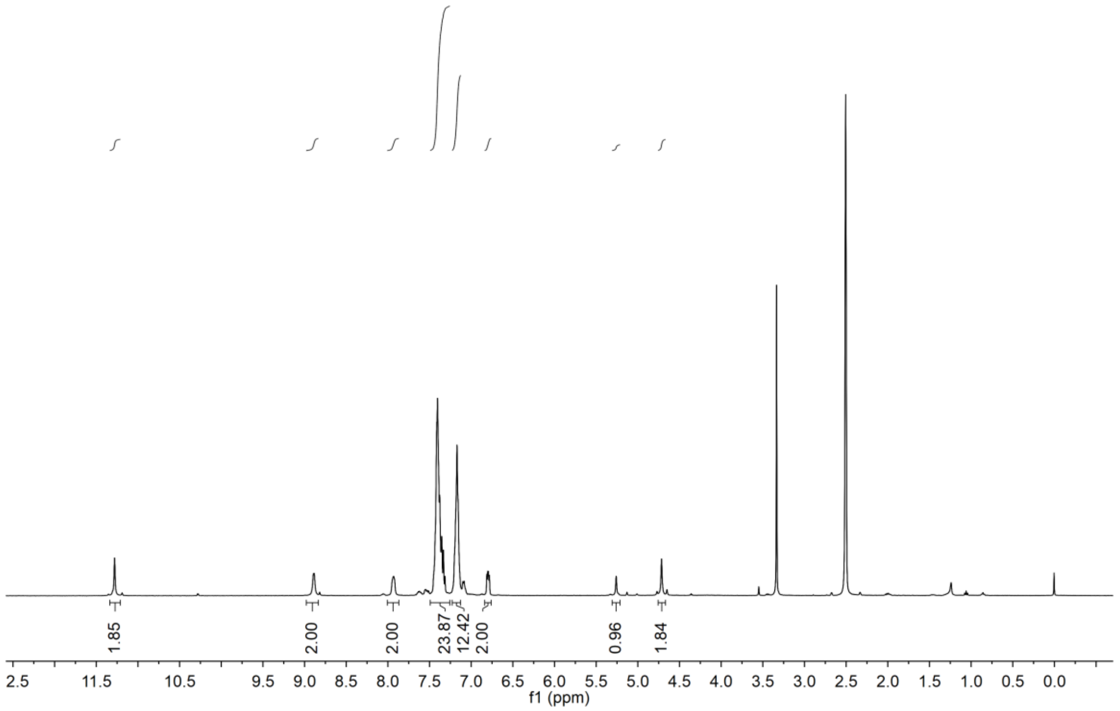

Figure S53. ^{1}H NMR spectrum of ligand $H_2\mathbf{L}^{\mathbf{P}}$ in DMSO-d_6 at 298 K.

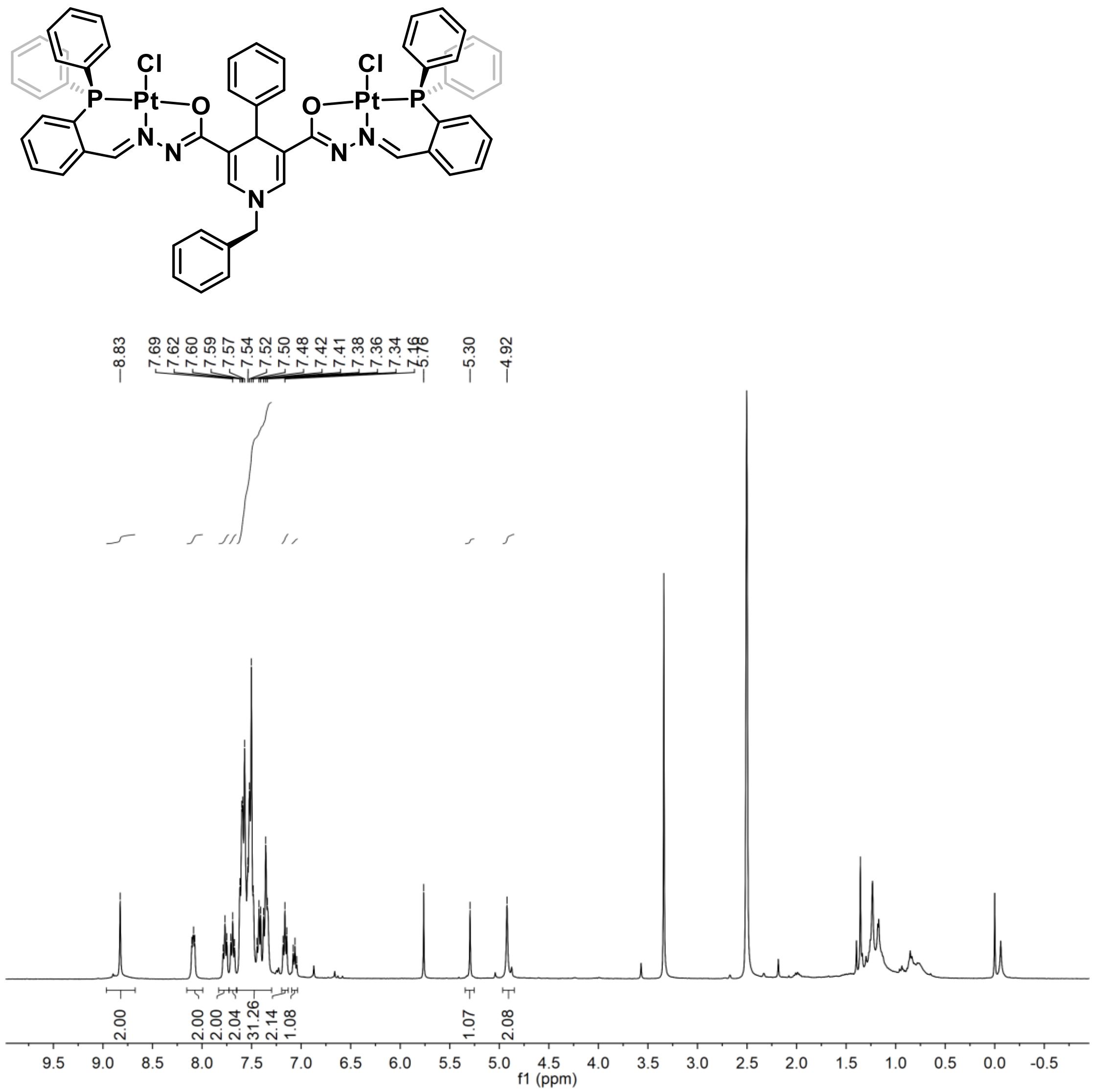

Figure S54. ^{1}H NMR spectrum of compound $Pt_2\mathbf{L^P}Cl_2$ in DMSO-d_6 at 298 K.

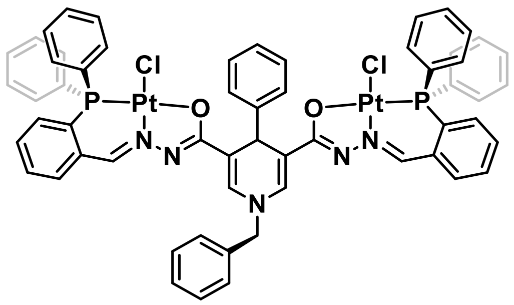

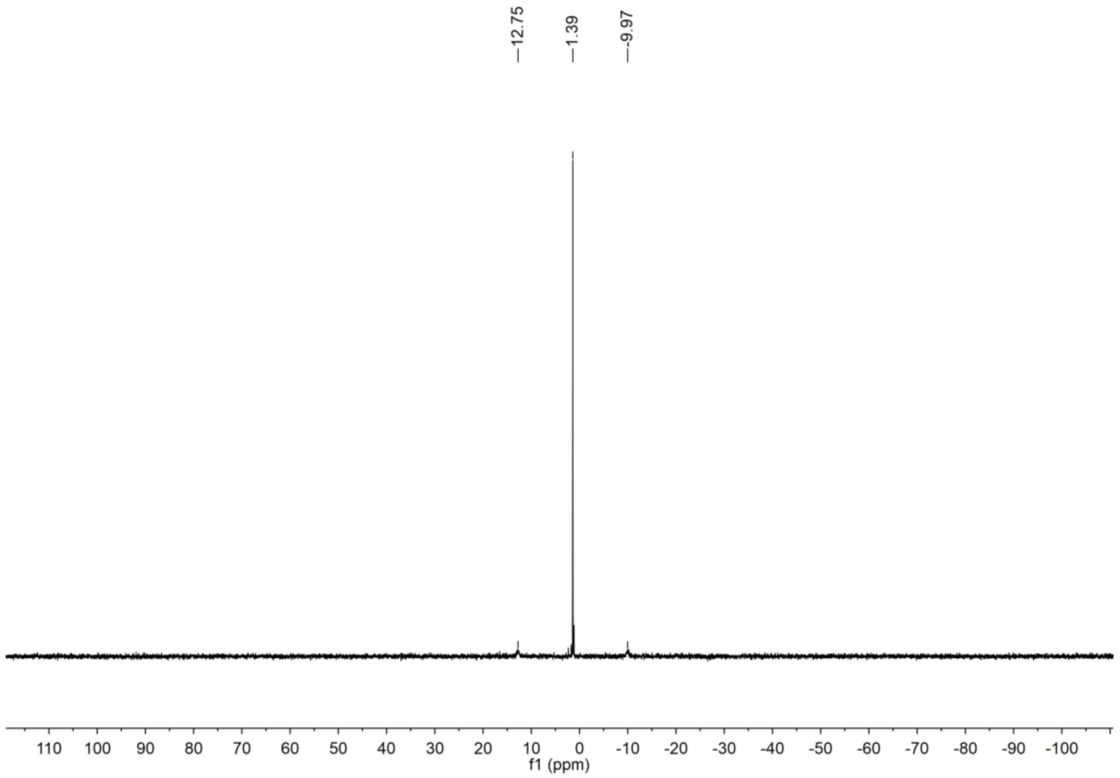

Figure S55. ^{31}P NMR spectrum of compound Pt_2**L**^{P}Cl_2 in DMSO-d_6 at 298 K.

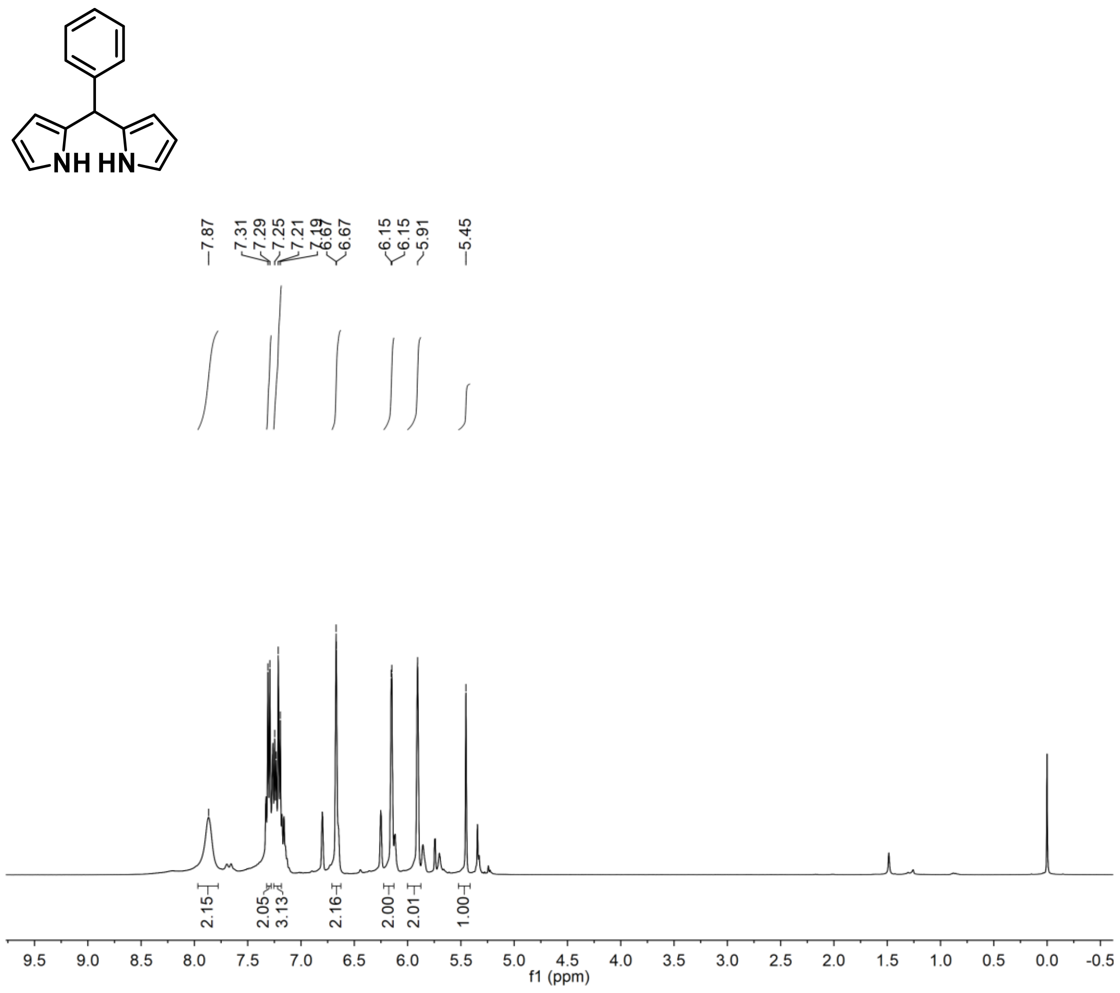

Figure S56. ^{1}H NMR spectrum of compound **3** in $CDCl_3$ at 298 K.

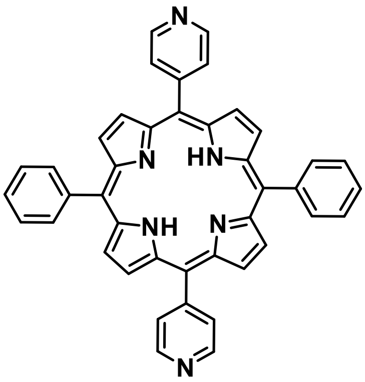

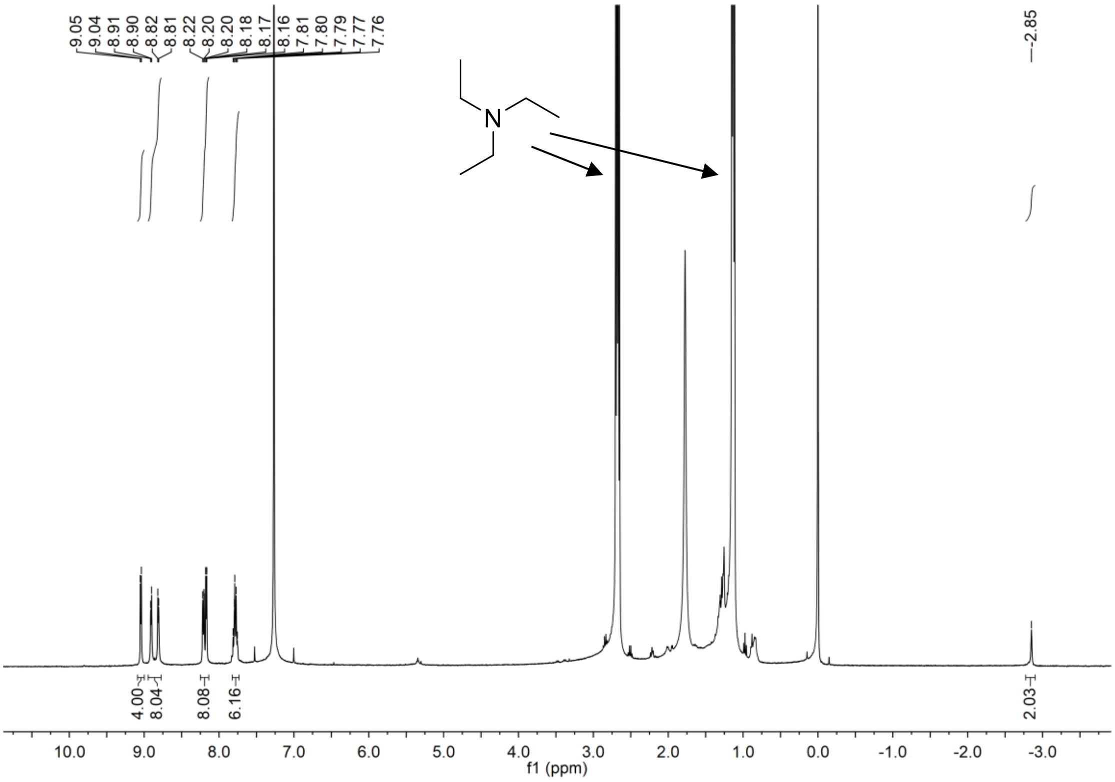

Figure S57. ^{1}H NMR spectrum of ligand $\mathbf{L^H}$ in $CDCl_3$ at 298 K.

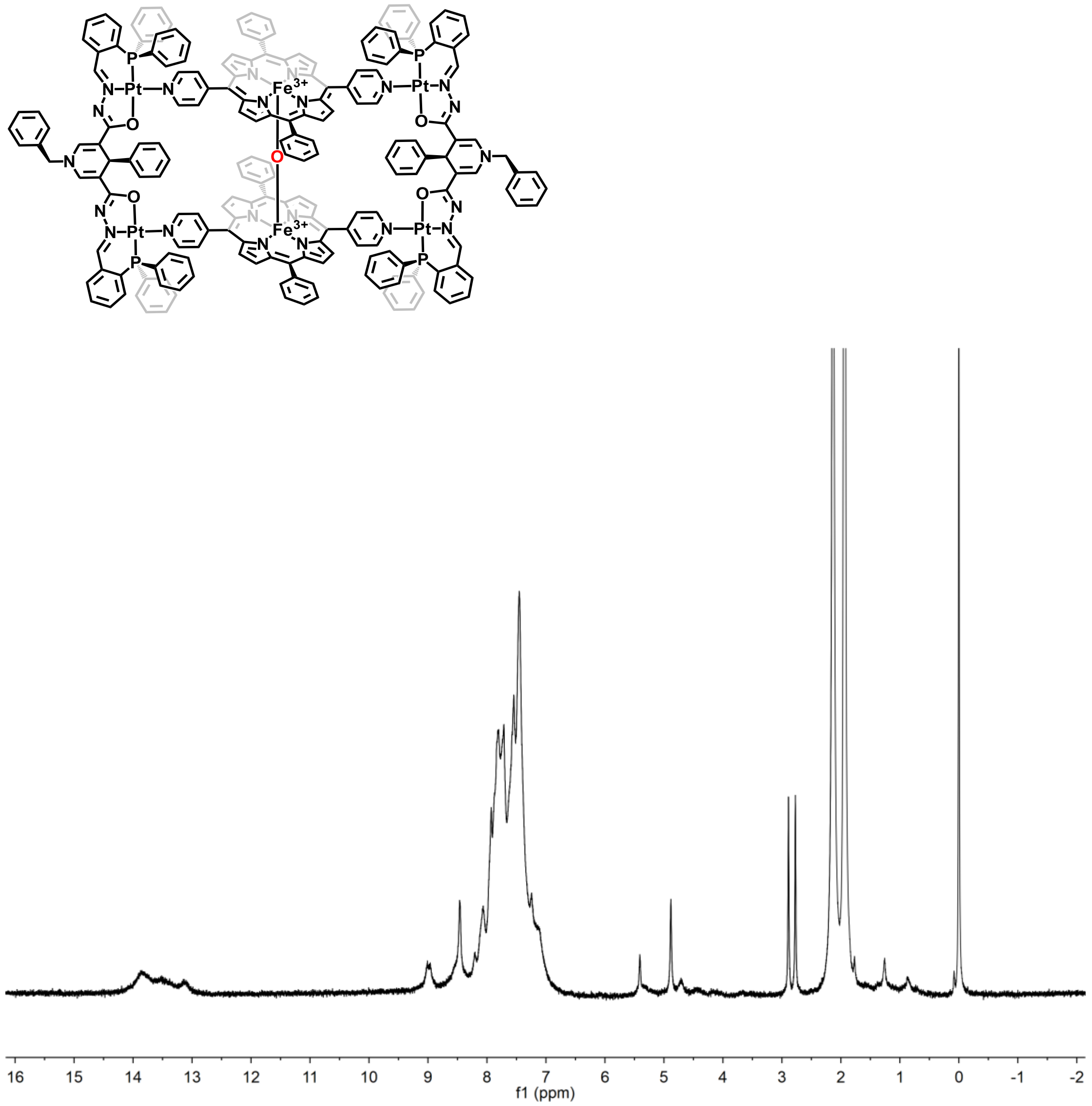

Figure S58. ^{1}H NMR spectrum of capsule **H1** (2.0 mM) in CD_3CN-d_3 at 298 K.

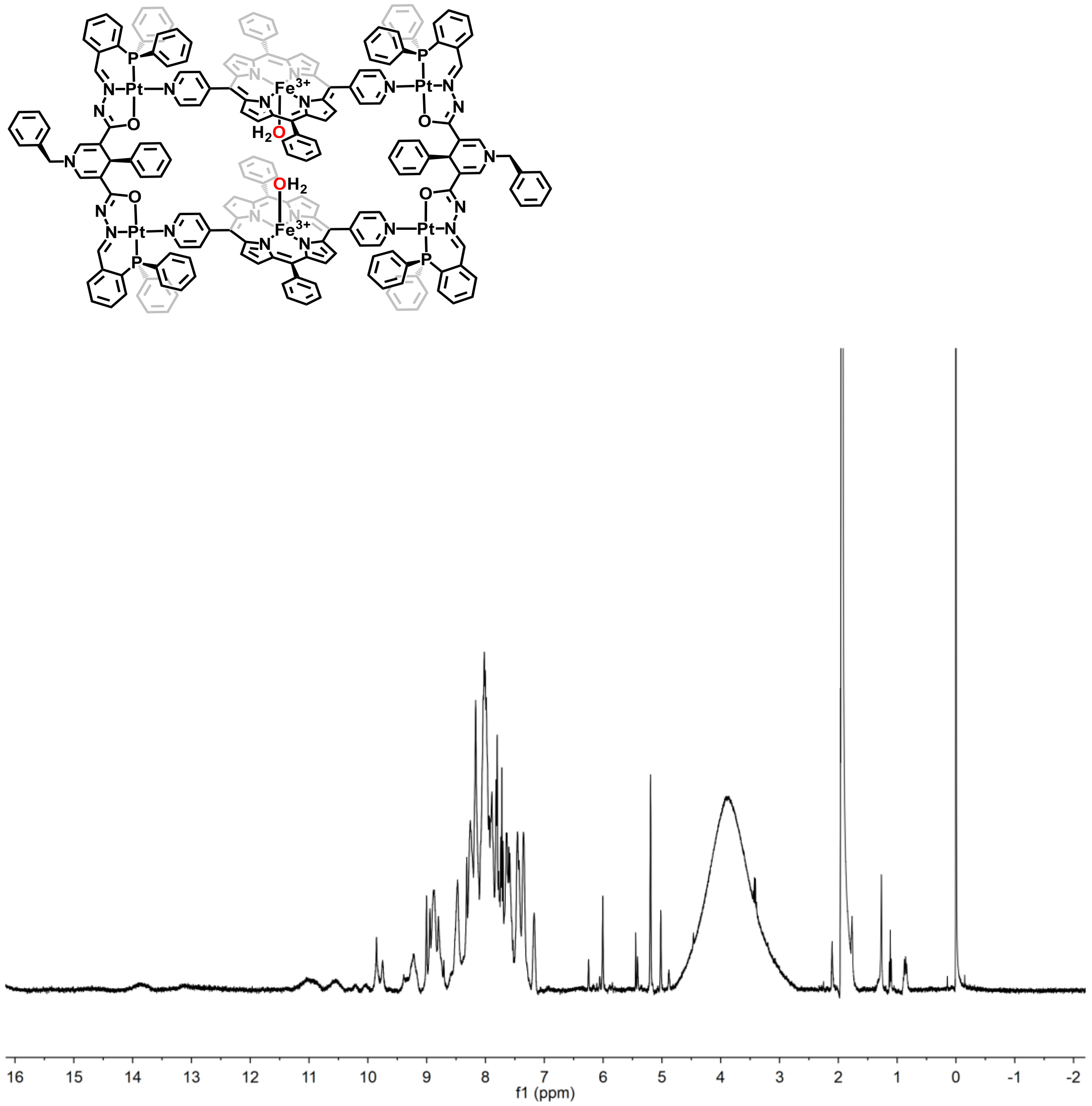

Figure S59. ^{1}H NMR spectrum of capsule **H1** (2.0 mM) upon the addition of HBF_4 (20.0 mM) in CD_3CN-d_3 at 298 K.

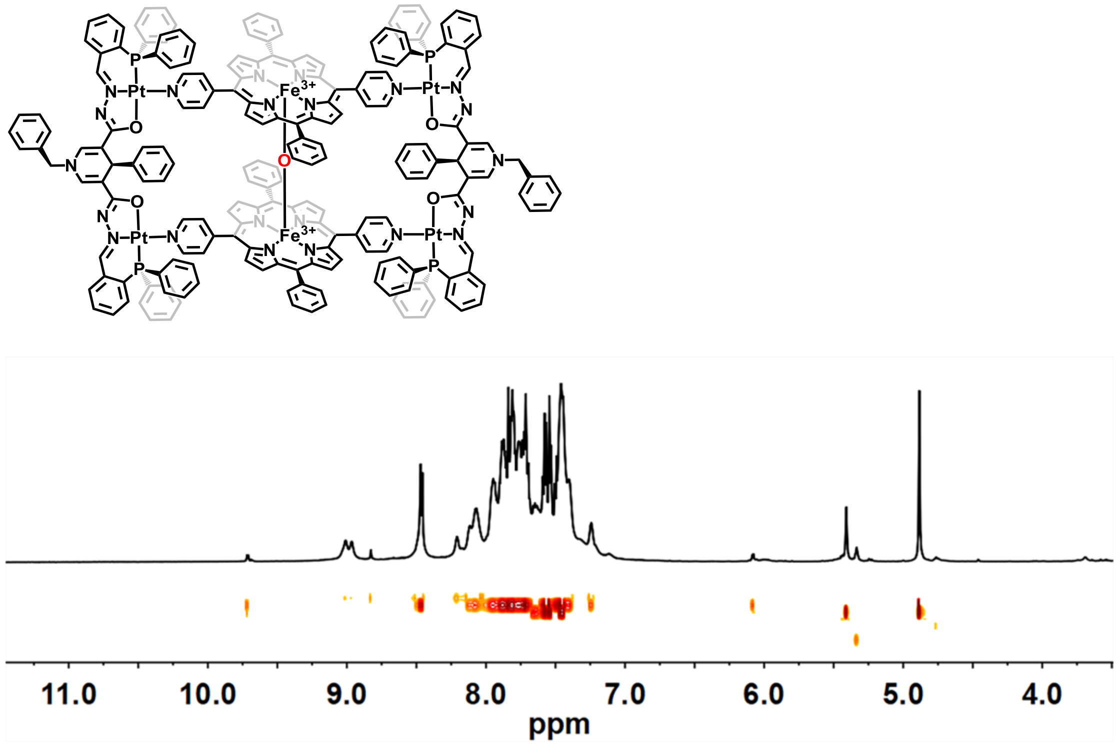

Figure S60. DOSY spectrum of capsule **H1** (2.0 mM) in CD_3CN-d_3 at 298 K.

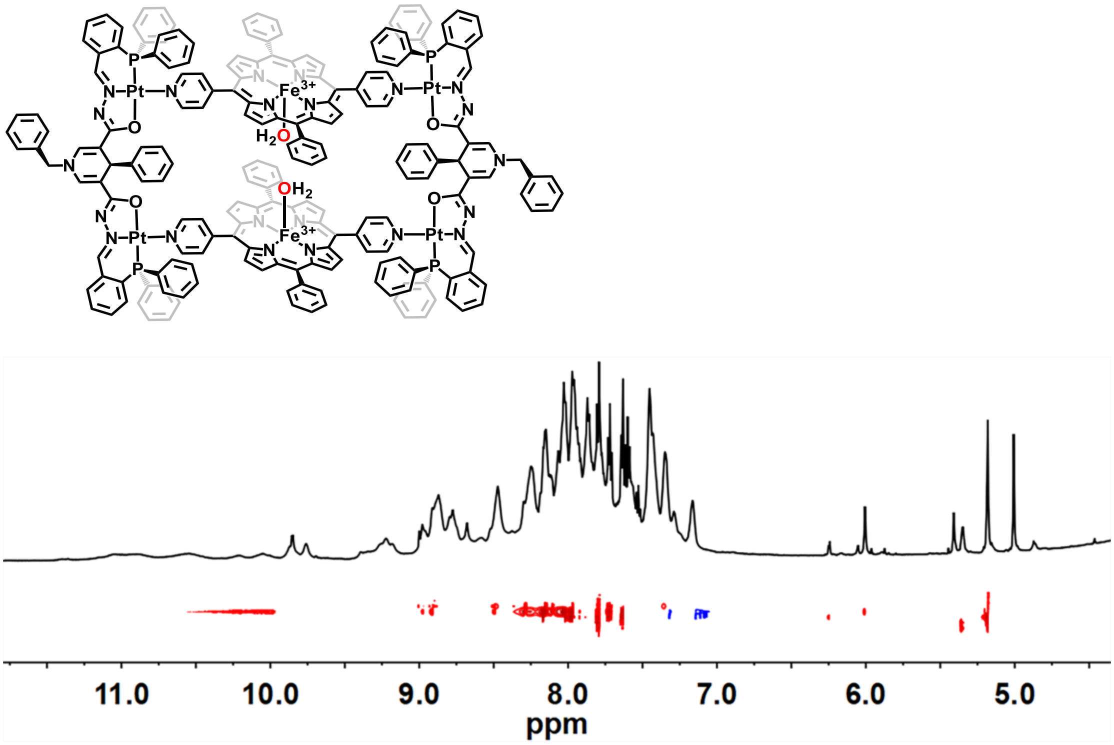

Figure S61. DOSY spectrum of capsule **H1** (2.0 mM) upon the addition of HBF_4 (20.0 mM) in CD_3CN-d_3 at 298 K.

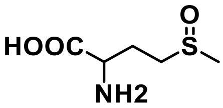

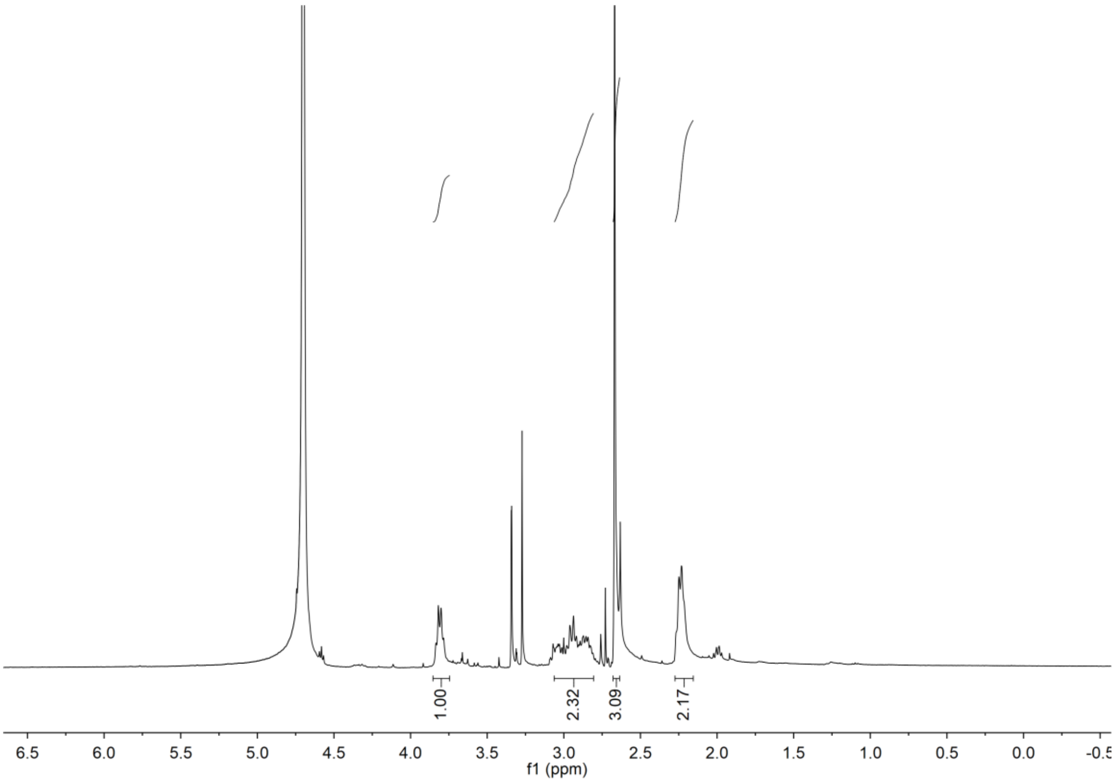

Figure S62. ^{1}H NMR spectrum of MetO generated in D_2O at 298 K.

9. References

[1] Cai, J. K., Zhao, L., Li, Y. N., He, C., Wang, C., & Duan, C. Y., Binding of Dual-Function Hybridized Metal–Organic Capsules to Enzymes for Cascade Catalysis, *JACS Au* **2022**, *2*, 1736–1746.

[2] Đorđević, M. M., Jeremić, D. A., Rodić, M. V., Simić, V. S., Brčeski, I. D., & Leovac, V. M., Synthesis, Structure and Biological Activities of Pd(II) and Pt(II) Complexes with 2-(Diphenylphosphino)Benzaldehyde 1-Adamantoylhydrazone, *Polyhedron* **2014**, *68*, 234–240.

[3] Lou, Z. R., Hou, Y. Q., Chen, K. P., Zhao, J. Z., Ji, S. M., Zhong, F. F., Ded, Y., et al., Different Quenching Effect of Intramolecular Rotation on the Singlet and Triplet Excited States of Bodipy, *J. Phys. Chem. C* **2018**, *122*, 185–193.

[4] SMART Data collection software (version 5.629); Bruker AXS Inc., Madison, WI, **2003**.

[5] SAINT, Data reduction software (version 6.45); Bruker AXS Inc., Madison, WI, **2003**.

[6] Sheldrick, G. M., Crystal Structure Refinement with SHELXL, *Acta Crystallogr. C Struct. Chem.* **2015**, *71*, 3–8.

[7] Dolomanov, O. V., Bourhis, L. J., Gildea, R. J., Howard, J. A. K., & Puschmann, H., OLEX2: A Complete Structure Solution, Refinement and Analysis Program, *J. Appl. Cryst.* **2009**, *42*, 339–341.

[8] Lozada, J., Lin, W. X., Cao-Shen, R. M., Tai, R. A., & Perrin, D. M., Salt Metathesis: Tetrafluoroborate Anion Rapidly Fluoridates Organoboronic Acids to give Organotrifluoroborates, *Angew. Chem. Int. Ed.* **2023**, *62*, e202215371.

[9] Mishra, S. S., Krishnaswamy, S., & Chand, D. K., Neighboring Cage Participation for Assisted Construction of Self-Assembled Multicavity Conjoined Cages and Augmented Guest Binding, *J. Am. Chem. Soc.* **2024**, *146*, 4473–4488.

[10] Wasser, I. M., Huang, H. W., Moënne-Loccoz, P., & Karlin, K. D., Heme/Non-Heme Diiron(II) Complexes and O_2, CO, and NO Adducts as Reduced and Substrate-Bound Models for the Active Site of Bacterial Nitric Oxide Reductase, *J. Am. Chem. Soc.* **2005**, *127*, 3310–3320.
